# Global, regional, and national burden of soft tissue and other extraosseous sarcomas, 1990–2021: A Systematic analysis for the global burden of disease study 2021

**DOI:** 10.1371/journal.pone.0342986

**Published:** 2026-03-09

**Authors:** Xingyue Yuan, Jianhong He, Ruibo Li

**Affiliations:** 1 Department of Pathology, Deyang People’s Hospital, Deyang, Sichuan Province, China; 2 Department of Orthopedics, The First Affiliated Hospital of Chongqing Medical University, Chongqing, China; 3 Department of Orthopaedics, Deyang People’s Hospital, Deyang, Sichuan Province, China; Gent University, BELGIUM

## Abstract

**Background:**

Soft tissue and other extraosseous sarcomas (STOES) are rare malignancies originating from mesenchymal tissues, posing a substantial health burden due to their aggressiveness and complex treatment. Understanding the global, regional, and national burden of STOES is crucial for effective prevention, screening, treatment, and resource allocation.

**Methods:**

Using the standardized Global Burden of Disease (GBD) methodology, we calculated STOES incidence, prevalence, mortality, and disability-adjusted life years (DALYs) to derive the burden of disease caused by STOES. Results were presented in numerical counts and age-standardized rates per 100,000 population, with an uncertainty interval (UI) to highlight potential statistical variability. The Joinpoint regression analysis was used to analyze the time trend from 1990 to 2021. The method facilitates the calculation of annual percentage change (APC) and average annual percentage change (AAPC) and their corresponding 95% confidence intervals (CI).

**Results:**

In 2021, the global burden of STOES remained substantial with 96,201 incident cases (ASIR: 1.16 per 100,000), 480,473 prevalent cases, 50,203 deaths (ASMR: 0.6 per 100,000), and 1,677,891 DALYs. Males had higher incidence, prevalence, mortality, and DALY rates than females. Regional disparities were evident, with high-income regions exhibiting higher incidence and prevalence but lower mortality rates compared to low-income regions. Notably, East Asia and Oceania had the lowest incidence rates, while Eastern Sub-Saharan Africa had the highest mortality and DALY rates. A mild downward trend was observed in incidence and prevalence, with more pronounced declines in mortality and DALY rates.

**Conclusion:**

Despite declining trends, the global burden of STOES remains significant, with notable regional differences. Tailored prevention, early detection, and treatment strategies, along with targeted resource allocation, are crucial. Further research is needed to understand the underlying factors driving these trends and develop effective interventions.

## Background

Soft tissue and other extraosseous sarcomas (STOES) are a group of malignant tumors originating from mesenchymal tissues, encompassing a wide range of histological subtypes, including but not limited to liposarcomas, leiomyosarcomas, synovial sarcomas, etc. [1,2]. STOES display a wide range of clinical behavior from low grade to high grade tumors that are characterized by an increased risk of metastatic spread. Although these tumors are not as prevalent as common cancers such as lung cancer and breast cancer globally, their high degree of heterogeneity, aggressiveness, and treatment complexity pose a significant burden on patients and society. A study by the French Sarcoma Group based on pathology reports and tumor blocks of all respective pathologists in the Rhone-Alpes region estimated a European age-standardized incidence of 4.9 per 100,000 persons per year for soft-tissue and visceral sarcoma [3]. In 2015, Asian-Pacific region (STAR) study reported the median overall survival in adult patients with metastatic STS was 11.7months and the 5-year survival rate was less than 10% worldwide [4]. With the intensification of global population aging, advancements in medical detection technologies, and increasing public attention to health, the incidence and diagnosis rates of soft tissue sarcomas and other extraosseous sarcomas have been rising annually, becoming a noteworthy issue in the field of public health [5,6].

Understanding the global, regional, and national burden of soft tissue sarcomas and other extraosseous sarcomas is crucial for formulating effective strategies for prevention, screening, treatment, and rehabilitation. This not only helps improve patients’ prognoses and quality of life but also provides a scientific basis for health policymakers to allocate medical resources reasonably and optimize cancer prevention and control strategies.

The Global Burden of Disease (GBD) study is a reliable tool for understanding the current assessment of the incidence, prevalence, mortality, and disability adjusted life years (DALYs) due to STOES. In previous assessments, STOES was often discussed as part of the broader disease burden of malignant neoplasms [7,8]. For the first time, the Global Burden of Diseases (GBD), Injuries, and Risk Factors Study 2021 (GBD 2021) database provides estimates for STOES as a separate disease entry. This study aims to comprehensively assess the global, regional, and national burden of soft tissue sarcomas and other extraosseous sarcomas, including incidence, prevalence, mortality, and disability-adjusted life years (DALYs), through a systematic analysis of data from GBD 2021 from 1990 to 2021. The GBD study is a large-scale epidemiological research project conducted by international organizations such as the World Health Organization (WHO) and the World Bank, aiming to provide comprehensive and comparable health estimation data for global health policy formulation [9].

This study will reveal the current status and trends of the global, regional, and national burden of soft tissue sarcomas and other extraosseous sarcomas through a comprehensive and systematic analysis, providing important references for the formulation of public health policies and the optimization of cancer prevention and control strategies. We anticipate that through this study, we can further enhance global awareness and attention to soft tissue sarcomas and other extraosseous sarcomas, promoting the further development of related research and prevention and control efforts.

## Methods

### Data sources

The study draws on data from the GBD 2021, which provides a comprehensive estimate of health losses from 371 diseases and injuries across 204 countries and territories from 1990 to 2021 [10], including STOES. The introduction and estimation methods of GBD 2021 are described in detail in the previous systematic analysis study for the GBD [10–12]. For nonfatal estimates, data were derived from scientific literature, household survey data, epidemiological surveillance data, disease registries, clinical informatics, and other sources, as well as searches of online research databases, government and international organization websites, published reports, and datasets provided by key data providers and GBD collaborators. Data for mortality estimates were primarily derived from vital registration, autopsy, survey, police, or surveillance data from all countries and territories. Consistent disease estimates were produced using epidemiological state-transition disease modeling software, DisMod-MR (Institute for Health Metrics and Evaluation), and Bayesian meta-regression software, MR-BRT (Institute for Health Metrics and Evaluation), which were adjusted for study-level differences in measurement methods and case definitions. When it was impossible to directly obtain data for certain regions, regional priors and national-level covariates were used to generate regional estimates. At the same time, uncertainty was estimated through 100 iterations of Bayesian models, and the 95% uncertainty intervals (UI) for each indicator are presented to assess the impact of data quality on the stability of the estimation results [10]. In this investigation, the estimates and their 95% UI for incidence, prevalence, mortality, and DALYs related to STOES were drawn from the GBD 2021 data.

Additionally, the study employed the sociodemographic index (SDI), a measure that quantifies a region’s sociodemographic progression based on income, education, and fertility circumstances. The SDI ranges from 0 to 1, with higher values representing higher levels of socioeconomic development. SDI is divided into five quintiles: low, low-middle, middle, high-middle, and high [8]. The relationship between the SDI and the incidence in different countries and regions was analyzed in this study.

### Joinpoint regression analysis

In this study, the Joinpoint regression analysis model, a statistical method commonly used in epidemiological studies, was used to assess the time trend of incidence [13]. The model facilitates the calculation of the annual percentage change (APC) and its accompanying 95% confidence interval (CI) to depict trends in incidence over a defined time frame. In addition, for a comprehensive assessment of observed trends, the average annual percentage change (AAPC), which contains aggregated trend data for the study period 1990–2021, was calculated. AAPC is a summary measure of the trend over a pre-specified fixed interval, calculated as the weighted average of the APC, allowing us to describe the average APC over the study period with a single number. The value of AAPC represents the annual percentage change (increase, decrease, or no change). For example, if the AAPC is 0.1, it means there is a 0.1% increase in the annual growth rate. When the estimated APC or AAPC value is above 0, it indicates an upward trend within the specified interval. By contrast, when the estimated APC or AAPC value is below zero, it suggests a downward trend. When the estimated APC or AAPC value is zero, it means that the trend remains stable [14,15].

All data used in this study wereobtained from publicly available databases; further ethical approval was not required.

### Statistics analysis

The incidence rate, prevalence rate, and mortality rate are expressed as predictions per 100,000 population, while DALYs rate is expressed per 100,000 person-years, including their 95% UI. All analyses and graphical representations were conducted using RStudio software (version 4.3.1) and the Joinpoint regression program (version 5.0.2).

## Results

### Overview of the global burden

The global number of incident cases increased from 54,630 (95% UI: 46,757–63,999) in 1990–96,201 in 2021 (95%UI: 83,423–116,185), while the age-standard incidence rate (ASIR) decreased from 1.21 per 100,000 population (95%UI: 1.04 to 1.39) in 1990 to 1.16 per 100,000 population (95%UI: 1.0 to 1.41) in 2021. In 2021, there were 52,347 male incident cases (95%UI: 43,223–68,477), with an ASIR of 1.34 per 100,000 population (95%UI: 1.11 to 1.74), and 43,854 female incident cases (95%UI: 38,483–51,362), with an ASIR of 1.01 per 100,000 population (95%UI: 0.88 to 1.19) (Table 1).

**Table 1:** The incidence and AAPCs of soft tissue and other extraosseous sarcomas from 1990 to 2021.

Location	Incidence in 1990		Incidence in 2019		AAPCs (95% CI), 1990 to 2021
	Counts (95% UI)	ASIR per 100,000 (95% UI)	Counts (95% UI)	ASIR per 100,000 (95% UI)	
Global	8,657 (22718 to 36835)	1.21 (1.04 to 1.39)	96,201 (83,424 to 116,185)	1.16 (1 to 1.41)	-0.25 (-0.32 to -0.18)
Sex					
Male	25,974 (20,633 to 30,560)	1.33 (1.07 to 1.68)	52,348 (43,223 to 68,478)	1.34 (1.11 to 1.74)	-0.06 (-0.12 to 0)
Female	361 (249 to 460)	1.11 (0.89 to 1.28)	43,853 (38,483 to 51,362)	1.01 (0.88 to 1.19)	-0.46 (-0.55 to -0.37)
Andean Latin America	472 (435 to 508)	1.2 (0.84 to 1.51)	647 (499 to 824)	1.04 (0.81 to 1.33)	-0.24 (-0.55 to 0.07)
Australasia	480 (408 to 572)	2.11 (1.94 to 2.27)	1,127 (981 to 1,283)	2.52 (2.18 to 2.88)	0.23 (-0.26 to 0.73)
Caribbean	347 (292 to 416)	1.54 (1.33 to 1.81)	691 (552 to 836)	1.39 (1.1 to 1.71)	0.11 (-0.09 to 0.32)
Central Asia	1,564 (1,421 to 1,712)	0.61 (0.52 to 0.74)	798 (663 to 943)	0.91 (0.76 to 1.08)	1.67 (1.3 to 2.04)
Central Europe	1,221 (1,130 to 1,325)	1.15 (1.05 to 1.26)	2,980 (2,666 to 3,282)	1.68 (1.51 to 1.86)	1.46 (1.25 to 1.68)
Central Latin America	493 (310 to 790)	0.97 (0.9 to 1.05)	3,441 (3,097 to 3,803)	1.34 (1.21 to 1.49)	0.81 (0.68 to 0.95)
Central Sub-Saharan Africa	6,169 (4,412 to 7,945)	1.08 (0.7 to 1.69)	913 (602 to 1,445)	1 (0.66 to 1.6)	-0.22 (-0.29 to -0.14)
East Asia	2,836 (2,359 to 3,144)	0.65 (0.46 to 0.83)	9,865 (6,953 to 13,828)	0.5 (0.35 to 0.7)	-1.03 (-1.14 to -0.91)
Eastern Europe	3,653 (2,682 to 5,651)	1.11 (0.93 to 1.24)	4,195 (3,758 to 4,626)	1.44 (1.28 to 1.58)	0.34 (0 to 0.68)
Eastern Sub-Saharan Africa	1,984 (1,845 to 2,198)	2.53 (1.93 to 3.82)	5,557 (3,916 to 8,748)	2.04 (1.48 to 3.2)	-0.95 (-1.04 to -0.86)
High-income Asia Pacific	8,590 (8,185 to 8,941)	1.07 (0.99 to 1.19)	3,884 (3,368 to 4,346)	1.17 (1.03 to 1.29)	0.21 (0.05 to 0.37)
High-income North America	10,195 (8,744 to 11,367)	2.68 (2.57 to 2.78)	14,069 (13,059 to 14,825)	2.63 (2.48 to 2.77)	-0.19 (-0.36 to -0.02)
High-middle SDI	18,831 (17,963 to 19,524)	1.01 (0.87 to 1.12)	17,875 (15,748 to 20,645)	1.02 (0.9 to 1.18)	-0.09 (-0.19 to 0.01)
High SDI	8,738 (6,305 to 11,300)	1.86 (1.78 to 1.93)	34,515 (31,573 to 36,742)	2.05 (1.9 to 2.16)	0.17 (0.01 to 0.33)
Low-middle SDI	6,528 (5,000 to 9,604)	0.96 (0.68 to 1.22)	14,332 (11,177 to 20,217)	0.88 (0.69 to 1.24)	-0.34 (-0.39 to -0.3)
Low SDI	10,281 (7,831 to 12,758)	1.58 (1.22 to 2.26)	9,870 (7,513 to 14,891)	1.25 (0.97 to 1.87)	-0.93 (-1.03 to -0.83)
Middle SDI	2,943 (2,200 to 4,071)	0.77 (0.58 to 0.94)	19,510 (15,577 to 24,983)	0.75 (0.6 to 0.97)	-0.16 (-0.21 to -0.12)
North Africa and Middle East	11 (8 to 16)	1.14 (0.81 to 1.51)	4,366 (3,269 to 6,211)	0.84 (0.63 to 1.18)	-1.01 (-1.05 to -0.96)
Oceania	8,533 (5,501 to 10,592)	0.26 (0.19 to 0.35)	21 (14 to 30)	0.2 (0.14 to 0.28)	-0.93 (-1 to -0.85)
South Asia	2,702 (2,000 to 3,692)	1.02 (0.65 to 1.26)	13,552 (10,001 to 19,135)	0.85 (0.63 to 1.21)	-0.75 (-0.85 to -0.66)
Southeast Asia	615 (542 to 694)	0.75 (0.55 to 1.02)	4,787 (3,710 to 6,797)	0.71 (0.55 to 1.01)	-0.46 (-0.57 to -0.34)
Southern Latin America	364 (257 to 475)	1.3 (1.14 to 1.46)	1,097 (948 to 1,234)	1.39 (1.19 to 1.57)	0.42 (0.21 to 0.63)
Southern Sub-Saharan Africa	1,080 (987 to 1,189)	0.9 (0.64 to 1.17)	847 (560 to 1,083)	1.22 (0.8 to 1.55)	1.23 (0.9 to 1.56)
Tropical Latin America	8,637 (8,158 to 9,052)	0.9 (0.82 to 0.98)	3,070 (2,842 to 3,308)	1.24 (1.14 to 1.34)	0.99 (0.86 to 1.13)
Western Europe	1,577 (1,100 to 2,623)	1.78 (1.68 to 1.86)	17,398 (15,679 to 18,764)	2.41 (2.21 to 2.59)	0.82 (0.63 to 1)
Western Sub-Saharan Africa	8,657 (22,718 to 36,835)	0.86 (0.61 to 1.44)	2,896 (1,995 to 4,519)	0.79 (0.57 to 1.2)	-0.2 (-0.26 to -0.13)

AAPCs, annual mean percentage changes. ASIR, age-standardized incidence rate. CI, confidence interval. UI, uncertainty interval.

The number of prevalent cases of STOES globally increased from 279,444 (95%UI: 238,598–327,229) in 1990–480,473 in 2021 (95%UI: 416,398–581,648), while the age-standard prevalence rate (ASPR) showed a slight decrease, from 5.95 per 100,000 population (95%UI: 5.14 to 6.86) in 1990 to 5.78 per 100,000 population in 2021 (95%UI: 5.01 to 7.02). In 2021, there were 258,405 prevalent cases in males (95% UI: 214,180–338,367) and 222,068 prevalent cases in females (95%UI: 195,203–260,703) (Table 2).

**Table 2:** The prevalence and AAPCs of soft tissue and other extraosseous sarcomas from 1990 to 2021.

Location	Prevalence in 1990		Prevalence in 2019		AAPCs (95% CI), 1990 to 2021
	Counts (95% UI)	ASPR per 100,000 (95% UI)	Counts (95% UI)	ASPR per 100,000 (95% UI)	
Global	279,444 (238,598 to 327,229)	5.95 (5.14 to 6.86)	480,473 (416,398 to 581,648)	5.78 (5.01 to 7.02)	-0.2 (-0.27 to -0.13)
Sex					
Male	145,618 (115,570 to 187,819)	6.37 (5.12 to 8.05)	258,405 (214,181 to 338,367)	6.47 (5.37 to 8.46)	-0.01 (-0.07 to 0.05)
Female	133,826 (106,157 to 158,262)	5.57 (4.48 to 6.5)	222,069 (195,204 to 260,703)	5.16 (4.53 to 6.11)	-0.4 (-0.49 to -0.31)
Andean Latin America	1,897 (1,308 to 2,413)	5.99 (4.18 to 7.55)	3,276 (2,511 to 4,184)	5.22 (4.02 to 6.67)	-0.21 (-0.52 to 0.09)
Australasia	2,371 (2,190 to 2,547)	10.63 (9.82 to 11.44)	5,633 (4,890 to 6,452)	13.11 (11.3 to 15.02)	0.33 (-0.17 to 0.83)
Caribbean	2,493 (2,094 to 2,983)	7.77 (6.63 to 9.18)	3,552 (2,813 to 4,351)	7.21 (5.66 to 9.01)	0.18 (-0.01 to 0.37)
Central Asia	1,773 (1,491 to 2,137)	3.03 (2.56 to 3.64)	4,009 (3,318 to 4,733)	4.45 (3.69 to 5.24)	1.64 (1.26 to 2.02)
Central Europe	7,705 (6,997 to 8,451)	5.67 (5.16 to 6.23)	14,328 (12,843 to 15,818)	8.48 (7.61 to 9.37)	1.56 (1.35 to 1.77)
Central Latin America	6,473 (5,972 to 7,046)	4.86 (4.52 to 5.27)	17,722 (15,937 to 19,576)	6.88 (6.19 to 7.61)	0.9 (0.77 to 1.04)
Central Sub-Saharan Africa	2,632 (1,634 to 4,248)	5.21 (3.36 to 8.27)	4,803 (3,163 to 7,598)	4.85 (3.19 to 7.78)	-0.18 (-0.26 to -0.1)
East Asia	31,193 (22,250 to 40,037)	3.06 (2.17 to 3.9)	47,560 (33,695 to 66,385)	2.39 (1.7 to 3.34)	-1 (-1.11 to -0.88)
Eastern Europe	14,139 (11,710 to 15,733)	5.57 (4.65 to 6.2)	20,570 (18,413 to 22,691)	7.24 (6.47 to 8.01)	0.39 (0.05 to 0.74)
Eastern Sub-Saharan Africa	19,069 (13,948 to 29,469)	11.79 (8.97 to 17.77)	29,023 (20,333 to 45,111)	9.69 (7.02 to 14.96)	-0.85 (-0.93 to -0.77)
High-income Asia Pacific	10,357 (9,654 to 11,482)	5.58 (5.19 to 6.21)	18,705 (16,265 to 20,825)	6.19 (5.39 to 6.81)	0.25 (0.1 to 0.4)
High-income North America	43,535 (41,792 to 45,106)	13.79 (13.3 to 14.29)	70,177 (65,534 to 73,751)	13.61 (12.86 to 14.3)	-0.16 (-0.33 to 0.01)
High-middle SDI	51,475 (44,205 to 57,381)	4.99 (4.29 to 5.56)	87,963 (77,475 to 101,631)	5.11 (4.5 to 5.88)	-0.04 (-0.14 to 0.06)
High SDI	94,732 (90,784 to 98,151)	9.5 (9.11 to 9.83)	170,221 (156,573 to 179,834)	10.61 (9.82 to 11.17)	0.23 (0.07 to 0.39)
Low-middle SDI	45,345 (32,884 to 58,699)	4.61 (3.27 to 5.8)	72,432 (56,937 to 101,994)	4.3 (3.37 to 6.01)	-0.3 (-0.34 to -0.26)
Low SDI	34,241 (25,811 to 50,317)	7.53 (5.78 to 10.77)	51,679 (39,230 to 77,446)	6.03 (4.66 to 9.06)	-0.87 (-0.96 to -0.78)
Middle SDI	53,361 (40,516 to 66,292)	3.74 (2.82 to 4.58)	97,689 (78,187 to 124,358)	3.72 (2.99 to 4.74)	-0.12 (-0.16 to -0.08)
North Africa and Middle East	15,590 (11,669 to 21,681)	5.62 (4.08 to 7.5)	22,712 (17,155 to 32,272)	4.18 (3.13 to 5.89)	-0.97 (-1.03 to -0.92)
Oceania	59 (43 to 81)	1.24 (0.9 to 1.72)	108 (75 to 156)	0.98 (0.68 to 1.38)	-0.84 (-0.92 to -0.77)
South Asia	43,928 (28,334 to 54,616)	4.85 (3.1 to 6.01)	67,641 (50,222 to 95,520)	4.09 (3.03 to 5.77)	-0.7 (-0.79 to -0.61)
Southeast Asia	14,147 (10,547 to 19,394)	3.71 (2.71 to 5.06)	24,276 (18,836 to 34,453)	3.5 (2.72 to 4.99)	-0.46 (-0.57 to -0.35)
Southern Latin America	3,111 (2,730 to 3,531)	6.48 (5.69 to 7.36)	5,513 (4,772 to 6,216)	7.1 (6.12 to 8.05)	0.49 (0.27 to 0.7)
Southern Sub-Saharan Africa	1,911 (1,347 to 2,482)	4.48 (3.18 to 5.81)	4,268 (2,833 to 5,490)	5.89 (3.9 to 7.52)	1.15 (0.87 to 1.43)
Tropical Latin America	5,632 (5,144 to 6,224)	4.45 (4.06 to 4.89)	15,721 (14,508 to 16,992)	6.36 (5.86 to 6.9)	1.09 (0.95 to 1.23)
Western Europe	42,924 (40,696 to 44,966)	9.09 (8.64 to 9.53)	85,343 (77,276 to 91,923)	12.56 (11.51 to 13.45)	0.88 (0.69 to 1.06)
Western Sub-Saharan Africa	8,508 (5,896 to 14,045)	4.24 (3 to 7.01)	15,533 (10,606 to 24,185)	3.85 (2.74 to 5.87)	-0.22 (-0.29 to -0.15)

AAPCs, annual mean percentage changes. ASPR, age-standardized prevalence rate. CI, confidence interval. UI, uncertainty interval.

In 2021, the global number of deaths due to STOES was 50,203 (95%UI: 43,232–61,280), with 27,163 male deaths (95%UI: 22,113–36,530) and 23,040 female deaths (95%UI: 19,9367–27,498). The age-standardized mortality rate (ASMR) was 0.6 per 100,000 population (95%UI: 0.52 to 0.74), showing a slight decrease compared to 0.74 per 100,000 population (95%UI: 0.62 to 0.86) in 1990, with an AAPC of −0.81 (95%CI: −0.88 to −0.74). However, the number of deaths increased compared to 1990, when the death toll was 31,878 (95%UI: 26,445–37,708) (Table 3).

**Table 3:** The mortality and AAPCs of soft tissue and other extraosseous sarcomas from 1990 to 2021.

Location	Deaths in 1990		Deaths in 2019		AAPCs (95% CI), 1990 to 2021
	Counts (95% UI)	ASMR per 100,000 (95% UI)	Counts (95% UI)	ASMR per 100,000 (95% UI)	
Global	31,878 (26,445 to 37,708)	0.74 (0.62 to 0.86)	50,203 (43,232 to 61,280)	0.6 (0.52 to 0.74)	-0.81 (-0.88 to -0.74)
Sex					
Male	16,624 (12,823 to 22,305)	0.83 (0.65 to 1.08)	27,162 (22,113 to 36,530)	0.7 (0.57 to 0.94)	-0.65 (-0.71 to -0.59)
Female	15,253 (12,009 to 18,025)	0.67 (0.54 to 0.78)	23,040 (19,936 to 27,497)	0.52 (0.45 to 0.63)	-1 (-1.09 to -0.91)
Andean Latin America	235 (167 to 292)	0.9 (0.64 to 1.12)	371 (286 to 472)	0.61 (0.48 to 0.78)	-1.06 (-1.38 to -0.75)
Australasia	215 (200 to 231)	0.95 (0.88 to 1.01)	459 (396 to 521)	0.94 (0.82 to 1.08)	-0.33 (-0.81 to 0.15)
Caribbean	290 (249 to 338)	0.98 (0.85 to 1.13)	393 (312 to 476)	0.78 (0.61 to 0.96)	-0.24 (-0.47 to -0.01)
Central Asia	209 (177 to 252)	0.4 (0.34 to 0.48)	441 (367 to 519)	0.53 (0.44 to 0.62)	1.17 (0.86 to 1.47)
Central Europe	894 (818 to 970)	0.65 (0.6 to 0.71)	1,480 (1,338 to 1,629)	0.76 (0.69 to 0.84)	0.7 (0.46 to 0.94)
Central Latin America	722 (689 to 752)	0.66 (0.63 to 0.69)	1,875 (1,686 to 2,068)	0.74 (0.67 to 0.82)	0.13 (0 to 0.26)
Central Sub-Saharan Africa	336 (225 to 534)	0.89 (0.59 to 1.4)	595 (389 to 940)	0.77 (0.49 to 1.26)	-0.45 (-0.49 to -0.42)
East Asia	3,980 (2,798 to 5,098)	0.46 (0.32 to 0.58)	4,963 (3,519 to 6,882)	0.25 (0.18 to 0.34)	-2.25 (-2.41 to -2.1)
Eastern Europe	1,592 (1,340 to 1,749)	0.61 (0.52 to 0.67)	2,144 (1,919 to 2,356)	0.68 (0.61 to 0.75)	-0.2 (-0.55 to 0.15)
Eastern Sub-Saharan Africa	2,576 (1,955 to 3,900)	2.17 (1.7 to 3.23)	3,579 (2,583 to 5,629)	1.58 (1.18 to 2.45)	-1.3 (-1.39 to -1.2)
High-income Asia Pacific	887 (824 to 987)	0.47 (0.44 to 0.53)	1,710 (1,473 to 1,894)	0.43 (0.38 to 0.47)	-0.36 (-0.55 to -0.17)
High-income North America	3,906 (3,734 to 4,017)	1.17 (1.13 to 1.21)	6,195 (5,731 to 6,469)	1.07 (1 to 1.11)	-0.48 (-0.65 to -0.3)
High-middle SDI	5,755 (4,890 to 6,435)	0.58 (0.5 to 0.65)	8,569 (7,524 to 9,839)	0.47 (0.41 to 0.54)	-0.91 (-1.03 to -0.79)
High SDI	8,945 (8,575 to 9,230)	0.86 (0.82 to 0.88)	15,148 (13,950 to 15,972)	0.81 (0.76 to 0.85)	-0.3 (-0.48 to -0.12)
Low-middle SDI	6,068 (4,313 to 7,677)	0.77 (0.54 to 0.96)	9,244 (7,217 to 12,794)	0.61 (0.48 to 0.84)	-0.88 (-0.94 to -0.82)
Low SDI	4,571 (3,585 to 6,635)	1.33 (1.02 to 1.87)	6,322 (4,881 to 9,481)	0.95 (0.75 to 1.42)	-1.29 (-1.38 to -1.19)
Middle SDI	6,504 (4,863 to 8,043)	0.55 (0.41 to 0.67)	10,866 (8,705 to 13,645)	0.42 (0.34 to 0.53)	-0.98 (-1.04 to -0.93)
North Africa and Middle East	1,752 (1,285 to 2,321)	0.79 (0.55 to 1)	2,234 (1,643 to 3,108)	0.46 (0.34 to 0.63)	-1.82 (-1.87 to -1.78)
Oceania	7 (5 to 10)	0.2 (0.14 to 0.27)	13 (9 to 18)	0.14 (0.1 to 0.2)	-1.11 (-1.19 to -1.03)
South Asia	6,072 (3,875 to 7,391)	0.84 (0.53 to 1.02)	8,932 (6,603 to 12,593)	0.59 (0.44 to 0.83)	-1.34 (-1.44 to -1.24)
Southeast Asia	1,744 (1,262 to 2,376)	0.55 (0.4 to 0.75)	2,872 (2,252 to 4,046)	0.44 (0.35 to 0.62)	-1 (-1.13 to -0.87)
Southern Latin America	373 (331 to 418)	0.8 (0.71 to 0.9)	571 (499 to 641)	0.69 (0.6 to 0.78)	-0.25 (-0.43 to -0.06)
Southern Sub-Saharan Africa	223 (158 to 288)	0.62 (0.44 to 0.81)	523 (355 to 661)	0.8 (0.54 to 1.01)	1.06 (0.68 to 1.44)
Tropical Latin America	666 (627 to 706)	0.61 (0.57 to 0.65)	1,720 (1,592 to 1,830)	0.69 (0.63 to 0.73)	0.34 (0.21 to 0.47)
Western Europe	4,184 (3,948 to 4,376)	0.81 (0.76 to 0.84)	7,437 (6,708 to 7,968)	0.91 (0.83 to 0.97)	0.25 (0.06 to 0.44)
Western Sub-Saharan Africa	1,004 (724 to 1,653)	0.67 (0.49 to 1.08)	1,687 (1190 to 2,534)	0.57 (0.42 to 0.86)	-0.49 (-0.56 to -0.42)

AAPCs, annual mean percentage changes. ASMR, age-standardized mortality rate. CI, confidence interval. UI, uncertainty interval.

The global DALYs due to STOES in 2021 were 1,677,891 person-years (95% UI: 1,428,208–2,115,701), with 916,038 person-years (95% UI: 731,625–1,309,813) for males and 761,853 person-years (95% UI: 654,091–946,756) for females. Overall, this represented an increase from 1990, when the global DALYs due to STOES were 1,355,265 person-years (95% UI: 1,117,320–16,708,06). The age-standardized DALY rate (ASDR) was 18.3 per 100,000 person-years (95% UI: 15.7 to 23.0), showing a decreasing trend compared to 1990, with an AAPC of −1.2 (95%CI: −1.28 to −1.12) (Table 4).

**Table 4:** The DALYs and AAPCs of soft tissue and other extraosseous sarcomas from 1990 to 2021.

Location	DALYs in 1990		DALYs in 2019		AAPCs (95% CI), 1990 to 2021
	Counts (95% UI)	ASDR per 100,000 (95% UI)	Counts (95% UI)	ASDR per 100,000 (95% UI)	
Global	1,355,268 (1,117,320 to 1,670,806)	27.4 (22.61 to 33.29)	1,677,891 (1,428,208-2,115,701)	20.54 (17.46 to 26.09)	-1.07 (-1.13 to -1)
Sex					
Male	725,914 (548,425 to 1,021,494)	29.87 (22.71 to 41.11)	916,038 (731,625 to 1,309,813)	22.99 (18.31 to 33.04)	-0.95 (-1 to -0.9)
Female	629,353 (467,615 to 783,060)	25.17 (18.74 to 30.84)	761,853 (654,091 to 946,756)	18.31 (15.68 to 23.04)	-1.2 (-1.28 to -1.12)
Andean Latin America	11,334 (7,934 to 14,357)	33.65 (23.86 to 41.8)	12,639 (9,627 to 16,279)	19.89 (15.18 to 25.59)	-1.51 (-1.83 to -1.19)
Australasia	6,788 (6,285 to 7,281)	30.96 (28.58 to 33.21)	12,418 (10,812 to 14,175)	30.81 (26.44 to 35.31)	-0.41 (-0.97 to 0.15)
Caribbean	13,633 (10,863 to 16,876)	40.19 (32.89 to 48.48)	16,015 (12,029 to 20,781)	33.74 (24.83 to 44.29)	-0.2 (-0.39 to -0.02)
Central Asia	8,655 (7,236 to 10,507)	14.3 (12.09 to 17.25)	15,806 (13,044 to 18,738)	17.18 (14.21 to 20.34)	0.79 (0.51 to 1.08)
Central Europe	30,745 (28,304 to 33,647)	23.11 (21.26 to 25.33)	39,744 (35,838 to 43,807)	25.08 (22.55 to 27.66)	0.46 (0.22 to 0.69)
Central Latin America	34,831 (33,345 to 36,424)	24.52 (23.44 to 25.54)	67,918 (60,879 to 75,100)	26.25 (23.53 to 29.07)	0.03 (-0.1 to 0.16)
Central Sub-Saharan Africa	19,908 (12,777 to 31,779)	36.66 (24.64 to 57.87)	29,078 (18,912 to 45,073)	27.6 (18.14 to 43.63)	-0.88 (-0.92 to -0.83)
East Asia	158,636 (113,891 to 205,561)	15.18 (10.81 to 19.61)	133,308 (94,782 to 187,768)	6.93 (4.93 to 9.81)	-2.88 (-3.03 to -2.72)
Eastern Europe	55,230 (46,085 to 60,858)	22.22 (18.7 to 24.42)	63,705 (57,291 to 70,235)	23.34 (20.87 to 25.78)	-0.46 (-0.84 to -0.09)
Eastern Sub-Saharan Africa	144,946 (106,858 to 221,262)	83.02 (63.67 to 124.2)	171,954 (121,266 to 272,847)	53.14 (38.49 to 83.55)	-1.69 (-1.78 to -1.61)
High-income Asia Pacific	33,552 (30,987 to 38,073)	18.48 (16.98 to 21.05)	40,354 (35,481 to 44,358)	15.2 (13.37 to 16.55)	-0.73 (-0.92 to -0.53)
High-income North America	127,490 (123,681 to 130,700)	41.34 (40.25 to 42.34)	173,362 (164,934 to 180,052)	35.69 (34.1 to 37)	-0.59 (-0.77 to -0.4)
High-middle SDI	212,759 (181,387 to 240,752)	20.56 (17.54 to 23.25)	244,695 (215,777 to 282,509)	14.86 (13.1 to 17.14)	-1.28 (-1.4 to -1.16)
High SDI	289,920 (280,449 to 298,394)	29.85 (28.95 to 30.76)	402,671 (378,751 to 420,655)	27.03 (25.54 to 28.19)	-0.45 (-0.64 to -0.26)
Low-middle SDI	301,445 (224,549 to 389,330)	28.98 (20.77 to 36.7)	357,408 (280,075 to 502,829)	20.64 (16.15 to 28.91)	-1.19 (-1.24 to -1.14)
Low SDI	257,544 (200,595 to 379,546)	52.72 (41.34 to 76.24)	308,353 (232,677 to 465,078)	33.41 (25.63 to 50.17)	-1.66 (-1.75 to -1.58)
Middle SDI	292,240 (222,251 to 364,035)	19.6 (14.77 to 24.28)	363,070 (292,599 to 457,257)	13.93 (11.21 to 17.61)	-1.24 (-1.29 to -1.19)
North Africa and Middle East	87,426 (67,030 to 121,517)	29.53 (21.89 to 39.54)	87,245 (66,215 to 123,402)	15.42 (11.57 to 21.7)	-2.13 (-2.18 to -2.08)
Oceania	352 (255 to 480)	7 (5.15 to 9.56)	596 (416 to 868)	5.11 (3.55 to 7.24)	-1.07 (-1.15 to -0.98)
South Asia	293,412 (197,558 to 358,162)	30.71 (19.81 to 37.5)	326,477 (245,106 to 462,756)	19.33 (14.53 to 27.35)	-1.68 (-1.77 to -1.6)
Southeast Asia	82,666 (61,429 to 113,149)	20.53 (14.96 to 28.14)	103,176 (80,571 to 146,440)	14.69 (11.5 to 20.97)	-1.36 (-1.47 to -1.24)
Southern Latin America	13,671 (12,021 to 15,427)	28.36 (24.94 to 31.98)	18,244 (15,817 to 20,676)	24.11 (20.82 to 27.52)	-0.33 (-0.51 to -0.14)
Southern Sub-Saharan Africa	10,913 (7,619 to 14,045)	24.44 (17.25 to 31.6)	21,909 (14,853 to 28,122)	29.53 (20.09 to 37.68)	0.9 (0.51 to 1.29)
Tropical Latin America	29,791 (28,060 to 31,772)	22.64 (21.31 to 24.05)	60,952 (56,778 to 64,973)	24.85 (23.08 to 26.59)	0.26 (0.13 to 0.4)
Western Europe	128,506 (122,200 to 134,345)	28.44 (27.13 to 29.7)	188,794 (174,125 to 201,377)	30.34 (28.15 to 32.31)	0.01 (-0.21 to 0.23)
Western Sub-Saharan Africa	62,771 (43,766 to 103,395)	28.58 (20.76 to 46.91)	94,187 (64,642 to 147,590)	20.92 (14.83 to 31.34)	-0.91 (-1 to -0.82)

AAPCs, annual mean percentage changes. CI, confidence interval. UI, uncertainty interval. DALYs, disability-adjusted life years.

### Soft tissue sarcomas and other extraosseous sarcomas burden by region

In 2021, research conducted at different regional levels around the world found that the ASIR of STOES was highest in regions with High SDI, with 2.05 per 100,000 population (95% UI: 1.9 to 2.16). The lowest is in the Middle SDI region, at 0.75 per 100,000 population (95% UI: 0.6 to 0.97). From 1990 to 2021, only High SDI regions showed an overall increase ASIR, with AAPC of 0.17 (95%CI: 0.01 to 0.33), while all other regions showed a decrease. Among them, the most decreased ASIR was in the Low SDI region with an AAPC of −0.93 (95%CI: −1.03 to −0.83). Geographically, High-income North America has the highest ASIR at 2.63 per 100,000 population (95% UI: 2.48 to 2.77), followed by Australasia at 2.52 per 100,000 population (95% UI: 2.18 to 2.88), and in Western Europe, 2.41 per 100,000 population (95% UI: 2.21 to 2.59). The regions with the lowest ASIR are Oceania, at 0.2 per 100,000 population (95% UI: 0.14 to 0.28), and East Asia, at 0.5 per 100,000 population (95% UI: 0.35 to 0.7). From 1990 to 2021, the regions with the largest increase in ASIR were Central Asia [AAPC: 1.67 (95% CI: 1.3 to 2.04)] and Central Europe [AAPC: 1.46 (95% CI: 1.25 to 1.68)], while the regions with the largest decrease were East Asia with an AAPC of −1.03 (95% CI: −1.14 to −0.91), and North Africa and Middle East with an AAPC of −1.01 (95% CI: −1.05 to −0.96) (Table 1).

The region with the highest ASPR in 2021 was High SDI, with an ASPR of 10.61 per 100,000 population (95% UI: 9.82 to 11.17). In contrast, the region with the lowest ASPR was Middle SDI, with a value of 3.72 per 100,000 population (95% UI: 2.99 to 4.74). From 1990 to 2021, the ASPR in the High SDI region showed an upward trend, with an AAPC of 0.23 (95% CI: 0.07 to 0.39), while the other regions exhibited a downward trend, with the most significant decline observed in the Low SDI region, with an AAPC of −0.87 (95% CI: −0.96 to −0.78). Geographically, in 2021, the region with the highest ASPR was High-income North America, with a value of 13.61 per 100,000 population (95% UI: 12.86 to 14.3), followed by Australasia with 13.11 per 100,000 population (95% UI: 11.3 to 15.02), and Western Europe with 12.56 per 100,000 population (95% UI: 11.51 to 13.45). On the other hand, the regions with the lowest ASPR were Oceania, with 0.98 per 100,000 population (95% UI: 0.68 to 1.38), East Asia, with 2.39 per 100,000 population (95% UI: 1.7 to 3.34), and Southeast Asia, with 3.5 per 100,000 population (95% UI: 2.72 to 4.99). Notably, from 1990 to 2021, Central Asia exhibited the most significant upward trend in ASPR, with an AAPC of 1.64 per 100,000 population (95% UI: 1.26 to 2.02), while East Asia showed the most pronounced downward trend, with an AAPC of −1 (95%CI: −1.11 to −0.88) (Table 2).

Across different SDI regions, the region with the highest ASMR in 2021 was the Low SDI, with a value of 0.95 per 100,000 population (95% UI: 0.75 to 1.42), while the region with the lowest ASMR was Middle SDI, at 0.42 per 100,000 population (95% UI: 0.34 to 0.53). From 1990 to 2021, all regions showed a downward trend in ASMR, with the most significant decline observed in Low SDI regions, with an AAPC of −1.29 (95%CI: −1.38 to −1.19). In terms of geographical regions, the area with the highest ASMR in 2021 was Eastern Sub-Saharan Africa, with 1.58 per 100,000 population (95% UI: 1.18 to 2.45), followed by High-income North America with 1.07 per 100,000 population (95% UI: 1 to 1.11). In contrast, the regions with the lowest ASMR were Oceania and East Asia, with 0.14 per 100,000 population (95% UI: 0.1 to 0.2) and 0.25 per 100,000 population (95% UI: 0.18 to 0.34), respectively. From 1990 to 2021, the region with the most significant increase in ASMR was Central Asia, with an AAPC of 1.17 (95%CI: 0.86 to 1.47), while the region with the most pronounced decrease was East Asia, with an AAPC of −2.25 (95%CI: −2.41 to −2.1) (Table 3).

The region with the highest ASDR in 2021 was Low SDI, with a value of 33.41 per 100,000 person-years (95% UI: 25.63 to 50.17), while the region with the lowest ASDR was Middle SDI, at 13.93 per 100,000 person-years (95% UI: 11.21 to 17.61). From 1990 to 2021, all regions showed a downward trend in ASDR, with the most significant decline observed in Low SDI regions, with an AAPC of −1.66 (95%CI: −1.75 to −1.58). Geographically, the region with the highest ASDR was Eastern Sub-Saharan Africa, with an ASDR of 53.14 per 100,000 person-years (95% UI: 38.49 to 83.55), followed by High-income North America with an ASDR of 35.69 per 100,000 person-years (95% UI: 34.1 to 37). The regions with the lowest ASDR were Oceania with 5.11 per 100,000 person-years (95% UI: 3.55 to 7.24) and East Asia with 6.93 per 100,000 person-years (95% UI: 4.93 to 9.81). From 1990 to 2021, the region with the most significant increase in ASDR was Southern Sub-Saharan Africa, with an AAPC of 0.9 (95%CI: 0.51 to 1.29), while the region with the most pronounced decrease in ASDR was East Asia, with an AAPC of −2.88 (95%CI: −3.03 to −2.72) (Table 4).

### Soft tissue sarcomas and other extraosseous sarcomas burden by country

**Fig 1:**
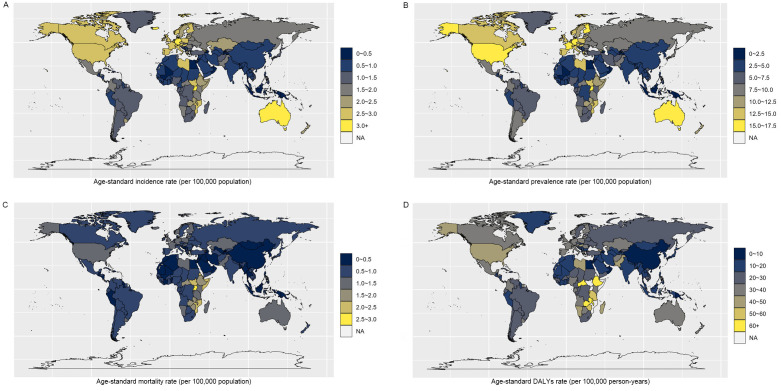
Geographical distribution of age-standardized rates of soft tissue and other extraosseous sarcomas in 2021. The map was created using the rmaps package in R (available at https://cran.r–project.org/web/packages/maps/index.html), which is an open – source software. The data used for the map comes from the 2021 incidence of Soft Tissue and Other Extraosseous Sarcomas, sourced from https://vizhub.healthdata.org/gbd-results/. (A) Age-standardized incidence rate. (B) Age-standardized prevalence rate. (C) Age-standardized mortality rate. (D) Age-standardized DALYs rate. Data source: Global Burden of Diseases, Injuries, and Risk Factors Study 2021. DALYs, disability-adjusted life-years.

At the national level, the countries with the highest ASIR in 2021 were Germany, with an ASIR of 4.78 per 100,000 population (95% UI: 4.16 to 5.32), Bermuda, with an ASIR of 4.43 per 100,000 population (95% UI: 3.55 to 5.76), and Sweden, with an ASIR of 4.38 per 100,000 population (95% UI: 3.67 to 5.15). On the other hand, the countries with the lowest ASIR were Northern Mariana Islands, with an ASIR of 0.03 per 100,000 population (95% UI: 0.01 to 0.04), Palau, with an ASIR of 0.05 per 100,000 population (95% UI: 0.02 to 0.08), and Tonga, with an ASIR of 0.11 per 100,000 population (95% UI: 0.07 to 0.18) (S1 Appendix and Fig 1A).

In 2021, the countries with the highest ASPR were Germany, with an ASPR of 23.40 per 100,000 population (95% UI: 20.48 to 26.02), Malta, with an ASPR of 21.86 per 100,000 population (95% UI: 17.91 to 26.51), and Bermuda, with an ASPR of 21.70 per 100,000 population (95% UI: 17.32 to 28.27). On the other hand, the countries with the lowest ASPR were Northern Mariana Islands, with an ASPR of 0.14 per 100,000 population (95% UI: 0.06 to 0.22), Palau, with an ASPR of 0.26 per 100,000 population (95% UI: 0.12 to 0.42), and Tonga, with an ASPR of 0.57 per 100,000 population (95% UI: 0.33 to 0.91) (S2 Appendix and Fig 1B).

In 2021, the countries with the highest ASMR were Bermuda [2.11 per 100,000 population (95% UI: 1.68 to 2.72)], Germany [2.07 per 100,000 population (95% UI: 1.80 to 2.29)], and Malta [1.87 per 100,000 population (95% UI: 1.53 to 2.25)], the same as ASIR and ASPR. The countries with the lowest ASMR remained the Northern Mariana Islands at 0.01 per 100,000 population (95% UI: 0.01 to 0.02), Palau at 0.03 per 100,000 population (95% UI: 0.01 to 0.05), and Tonga at 0.57 per 100,000 population (95% UI: 0.33 to 0.91) (S3 Appendix and Fig 1C).

From a national perspective, the countries with the highest ASDR in 2021 were Haiti, with an ASDR of 57.75 per 100,000 person-years (95% UI: 34.18 to 84.96), South Sudan, with an ASDR reported as 57.06 per 100,000 person-years (95% UI: 34.82 to 95.80), and Barbados, with an ASDR of 54.50 per 100,000 person-years (95% UI: 42.75 to 68.38). Meanwhile, the countries with the lowest ASDR remained Northern Mariana Islands, with an ASDR of 0.54 per 100,000 person-years (95% UI: 0.23 to 0.84), Palau, with an ASDR of 1.01 per 100,000 person-years 95% UI: (0.47 to 1.63), and Tonga, with an ASDR of 2.47 per 100,000 person-years (95% UI: 1.46 to 3.99) (S4 Appendix and Fig 1D).

### Effects of different SDI on Incidence rate

**Fig 2:**
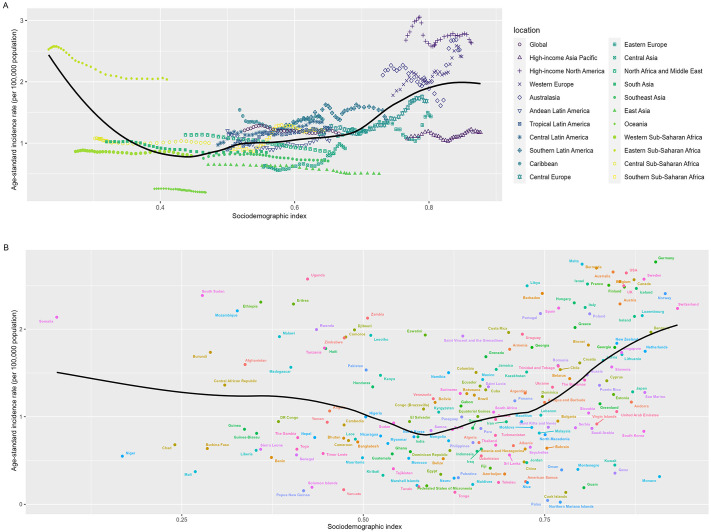
Effects of different sociodemographic index on Incidence rate at the regional level from 1990 to 2021 (A) and national level at 2021 (B). Data source: Global Burden of Diseases, Injuries, and Risk Factors Study 2021.

Between 1990 and 2021, at the regional level, there was a nonlinear relationship between ASIR and the SDI. As the SDI increased, the ASIR exhibited intermittent declines or rises. From 1990 to 2021, Oceania and East Asia regions experienced a mild downward trend in incidence rates, which were lower than expected. Across all regions within the Sub-Saharan Africa super-region, the ASIR remained relatively stable with no significant changes. In high SDI regions such as High-income Asia Pacific, Western Europe, and High-income North America, the ASIR showed intermittent increases or decreases between 1990 and 2021. However, Western Europe and High-income North America had ASIRs that were higher than expected, while the ASIR in High-income Asia Pacific was significantly lower than expected (Fig 2A).

At the national level, the analysis revealed that a nonlinear relationship also existed between ASIR and SDI from 1990 to 2021. The high burden of STOES exists not only in high SDI countries but also in low SDI countries. High SDI countries such as Germany, Malta, Bermuda, and Australia, as well as low SDI countries like South Sudan, Somalia, Mozambique, and Ethiopia, had ASIRs that were much higher than expected. Conversely, countries like Monaco, Northern Mariana Islands, Mali, and Niger had ASIRs that were far lower than anticipated (Fig 2B).

### Joinpoint regression analysis

**Fig 3:**
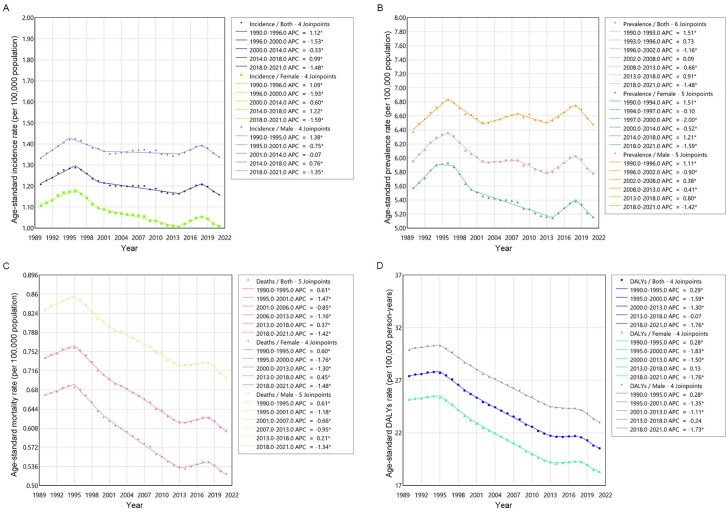
Joinpoint regression analysis of incidence rate (A), prevalence rate (B), mortality rate (C) and DALYs rate (D) of soft tissue and other extraosseous sarcomas from 1990 to 2021. * Indicates that the APC is significantly different from zero at the alpha = 0.05 level. DALYs, disability-adjusted life-years. APC = annual percentage change. Data source: Global Burden of Diseases, Injuries, and Risk Factors Study 2021.

The Joinpoint regression analysis showed that from 1990 to 2021, both ASIR and ASPR exhibited an overall mild downward trend, while ASMR and ASDR showed more significant declines. Regardless of whether it was ASIR, ASPR, ASMR, or ASDR, at the same time point, the rates were consistently higher in males than in females. The ASIR increased during the periods from 1990 to 1996 and 2014–2018, while it decreased during the remaining time periods, with the most significant decline occurring after 2018. Similarly, the most pronounced downward trends in ASPR, ASMR, and ASDR were also observed after 2018 (Fig 3).

### Soft tissue sarcomas and other extraosseous sarcomas burden by age group

**Fig 4:**
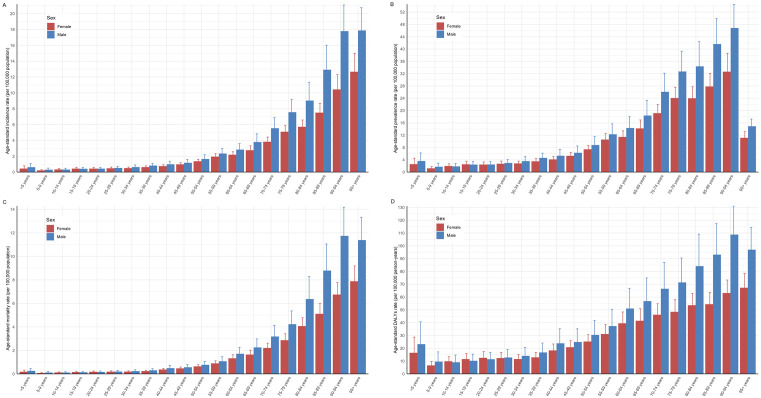
Age-specific burden of soft tissue and other extraosseous sarcomas in 2021. (A) Age-specific incidence rate (per 100,000 population). (B) Age-specific prevalence rate (per 100,000 population). (C) Age-specific morality rate (per 100,000 population). (D) Age-specific DALYs rate (per 100000 person-years). DALYs, disability-adjusted life-years. Error bars denote 95% uncertainty intervals. Data source: Global Burden of Diseases, Injuries, and Risk Factors Study 2021.

The incidence of STOES gradually increased with age, especially after the age of 60. Men generally had a higher incidence than women across all age groups, and the difference was particularly pronounced in middle age and beyond. Children and adolescents (<5 years, 5–9 years, 10–14 years, 15–19 years): The incidence was relatively low and the differences between males and females were not significant. Early adulthood (ages 20–29): Incidence begins to increase, but remains low. Middle age (30–49 years): The incidence gradually increased, and the incidence began to be significantly higher in men than in women. Middle-aged and elderly (50–69 years): Incidence increased rapidly, especially among men, reaching a peak in the 65–69 age group. Old age (70 years and older): Incidence continued to rise, and the trend was more pronounced, especially in the age group 75 years and older (Fig 4A). ASPR, ASMR and ASDR showed similar trends to ASIR (Fig 4B, Fig 4C, Fig 4D). The rapid increase in ASMR occurred after the age of 55–59 years.

## Discussion

This study comprehensively assessed the burden of STOES globally from 1990 to 2021, revealing trends at different time points. Although the global number of incident cases increased from 54,630 in 1990–96,201 in 2021, the ASIR decreased, indicating that despite the rise in absolute case numbers, the relative incidence of STOES, taking into account population growth and aging, is actually declining. However, it is noteworthy that while the ASMR and ASDR decreased, their absolute values still increased, potentially reflecting the high lethality of STOES and its severe impact on patients’ quality of life. A study from Germany found that one- and five-year relative survival rates were 87.8% and 66.4% for soft tissue sarcoma [6]. Another retrospective case review involving the United Kingdom, France, Germany, and Spain showed that adult patients with advanced sarcoma had a median survival of just 17.6 months after beginning treatment [16].

The study results demonstrated significant differences in the burden of STOES across regions with varying SDI levels. High-income regions (such as high-income North America, Australasia, and Western Europe) generally had higher ASIR and ASPR, which could be attributed to their superior medical detection capabilities and more comprehensive disease registration systems. However, from 1990 to 2021, only high-income regions showed an overall increase in ASIR, while all other SDI regions experienced a decline, suggesting that high-income regions may have an advantage in early detection and diagnosis of STOES. Additionally, low-SDI regions had higher ASMR and ASDR, especially the Southern Sub-Saharan Africa, indicating greater challenges in STOES management and treatment in these areas. The complex health care facilities and measures needed to treat cancer are often lacking in these areas [17]. A study from the United Kingdom showed that patients with soft tissue sarcoma in deprived areas had a 23% higher risk of dying within five years than those in non-deprived areas [18]. Therefore, it is crucial to develop differentiated prevention and control strategies tailored to the specific situations of different SDI regions.

When further exploring the underlying reasons for these regional disparities, occupational or environmental exposure factors cannot be overlooked. They are likely to play a significant role in explaining the differences in incidence rates between high – and low – income regions. Previous studies have demonstrated that occupational exposure to certain chemicals is associated with an increased risk of developing soft tissue sarcomas [19,20]. High – income regions may experience relatively higher incidence rates due to factors such as industrial activities, medical radiation, and occupational chemical exposure. In contrast, although the forms of industrial pollution and chemical exposure in low – income regions may differ, the incidence data may not truly reflect the actual situation because of limited medical resources and an imperfect monitoring system.

Therefore, in global cancer prevention strategies, it is essential to fully consider occupational or environmental exposure factors and formulate targeted prevention and control measures according to the exposure characteristics of different regions. For high – income regions, it is necessary to strengthen industrial pollution control, standardize the use of medical radiation, and enhance the level of occupational health protection. For low – income regions, efforts should be made to increase the intensity of environmental pollution control, establish a comprehensive monitoring system for radiation and chemical exposure, and raise residents’ awareness of environmental hazards and their self – protection capabilities. Through the implementation of these measures, it is expected to narrow the gap in cancer incidence rates between different regions and improve the overall effectiveness of global cancer prevention and control.

This study also emphasized the gender differences in STOES. Males consistently had higher ASIR, ASPR, ASMR, and ASDR than females, both globally and regionally. A population-based epidemiological analysis conducted in Europe from 1996 to 2015 found slightly higher age-standardized incidence of soft tissue sarcoma in men than in women [21]. Another study from Germany also confirmed sex differences in the incidence of different subtypes of soft tissue sarcoma [22]. This disparity may be related to sex hormone levels, genetic susceptibility, and lifestyle choices (such as smoking and alcohol consumption). Another possible explanation may be related to the exposure to potential mutagens in occupations that are predominantly male-dominated [2]. Previous studies on the relationship between occupational exposure and sarcomas have found higher incidence rates among gardeners, railway workers, farmers, farm managers, workers in the pulp and paper industry, construction sites, chemical plants, meat processing and woodworking facilities, as well as nuclear facilities [23]. Some environmental genotoxic substances, such as vinyl chloride, dioxins, and chlorophenols, have been studied for their potential contribution to the pathogenesis of soft tissue sarcoma [24]. As such, when formulating prevention and control strategies, special attention should be given to the male population, with enhanced health education and early screening efforts to improve early detection and treatment success rates.

Joinpoint regression analysis revealed an overall mild downward trend in ASIR and ASPR of STOES from 1990 to 2021, while ASMR and ASDR showed more pronounced declines. This trend may be associated with advancements in medical technology, heightened public health awareness, and the implementation of disease prevention and control measures globally. These include efforts made by global health organizations such as the 2011 United Nations Political Declaration on the Prevention and Control of Non-communicable Diseases [25] and initiatives aimed at Preventing Noncommunicable Diseases (NCDs) by Reducing Environmental Risk Factors [26]. However, it is worth noting that the ASIR increased during specific time periods, such as from 1990 to 1996 and from 2014 to 2018. Unfortunately, we are unable to pinpoint the exact reasons for these increases during these particular time periods. We speculate that they may be linked to changes in disease monitoring systems. Future studies should delve deeper into these potential influencing factors to facilitate the development of more effective prevention and control strategies.

The incidence of STOES gradually increased with age, particularly after 60 years, mirroring the trend observed in most malignancies [17] and is consistent with previous research findings on the clinical characteristics of soft tissue sarcomas [27–29]. The study also confirms that compared to younger individuals, elderly patients with soft tissue sarcomas are more susceptible to tumor recurrence, and the incidence of tumor-related comorbidities is significantly higher in elderly patients. Furthermore, the more comorbidities a patient has, the worse the prognosis for tumor treatment tends to be [28,30]. Therefore, prevention and control strategies should prioritize the older population, with intensified health education and early screening initiatives.

In subsequent tumor prevention and control efforts, it is crucial to establish a regular occupational health monitoring system for high-risk professions to promptly identify and address potential health issues. Additionally, strengthening health education to raise public awareness and importance of STOES, as well as encouraging healthy lifestyles to reduce the risk of developing the disease, is imperative. Furthermore, an effective early screening mechanism should be established to conduct periodic screenings among high-risk populations. Simultaneously, advancing research and application of STOES treatment technologies tailored to the characteristics of different age groups, genders, and geographical regions is necessary to develop personalized prevention and intervention strategies. Ultimately, these measures aim to improve the survival rates and quality of life for STOES patients.

The findings of this study on STOES not only provide crucial evidence for the prevention and control of this specific type of tumor but also offer universal insights for the formulation and optimization of global cancer prevention strategies, extending beyond the scope of soft tissue sarcomas alone. By emphasizing measures such as early screening and detection, paying attention to regional disparities, recognizing gender differences, focusing on the elderly population, and strengthening occupational health monitoring, a more comprehensive and effective global cancer prevention and control system can be established. This will contribute to reducing the incidence and mortality rates of cancer, improving patients’ quality of life, and making positive contributions to global public health endeavors.

This study has significant strengths. The data is sourced from the authoritative and comprehensive Global Burden of Disease (GBD) 2021 database, with a wide and representative sample. The use of rigorous methodologies enhances the reliability and validity of the results. The analysis is thorough and in-depth, covering multiple dimensions of the burden of soft tissue and other extraosseous sarcomas (STOES). By employing regression analysis, it provides insights into disease dynamics, facilitating an understanding of the burden and trends at the global, regional, and national levels. The research findings can serve as a basis for formulating public health policies and cancer prevention strategies, while the gender- and age-specific analyses are conducive to the development of personalized plans.

However, the study also has limitations. The availability and quality of data are affected by some literature reports and model estimates. Differences in data collection and reporting practices across countries impact the comparability and accuracy of the results. There is a time lag between data collection and inclusion in the database, which may obscure recent trends. The analysis may not fully reflect the impact of advancements in medical technology. Moreover, due to regional differences, the generalizability of the results is limited. Future research needs to incorporate more variables to improve specificity and applicability.

Overall, although the study offers robust and comprehensive insights, accurate interpretation requires acknowledging its limitations. Addressing these challenges will deepen understanding and contribute to the development of more effective prevention and control strategies.

## Conclusion

In conclusion, despite the downward trends observed in the ASIR, ASPR, ASMR, and ASDR of STOES over the past three decades, the disease burden remains significant, with notable differences between regions and countries. To effectively address this challenge, it is essential to strengthen international cooperation and information sharing to collaboratively research and develop prevention and control strategies. Efforts should also be intensified in the research and development of new technologies and medications for the treatment of STOES. Furthermore, it is crucial to devise specific intervention measures tailored to the characteristics of different regions, countries, and age groups.

## Supporting information

S1 Appendix Table 1. Age-standardized incidence rate (per 100,000 population) and incident cases in 204 countries in 1990 and 2021.
(DOCX)

S2 Appendix Table 2. Age-standardized prevalence (per 100,000 population) and prevalent cases in 204 countries in 1990 and 2021.
(DOCX)

S3 Appendix Table 3. Age-standardized mortality rate (per 100,000 population) and death cases in 204 countries in 1990 and 2021.
(DOCX)

S4 Appendix Table 4. Age-standardized DALYs rate (per 100,000 person-years) and DALYs counts in 204 countries in 1990 and 2021.
(DOCX)

## Abbreviations

AAPC: average annual percentage change
APC: annual percentage change
ASDR: age-standardized DALY rate
ASIR: age-standard incidence rate
ASMR: age-standardized mortality rate
ASPR: age-standard prevalence rate
CI: confidence intervals
DALYs: disability adjusted life years
GBD: Global Burden of Disease
SDI: sociodemographic index
STOES: soft tissue sarcomas and other extraosseous sarcomas
UI: uncertainty interval.
