# Supplementary material for: Global, regional, and national burden of soft tissue and other extraosseous sarcomas, 1990–2021: A Systematic analysis for the global burden of disease study 2021
Source: PLoS One. 2026 Mar 9;21(3):e0342986. doi: 10.1371/journal.pone.0342986 (PMC12970919; doi:10.1371/journal.pone.0342986)
Supplement: S1 Appendix — Age-standardized incidence rate (per 100,000 population) and incident cases in 204 countries in 1990 and 2021. (DOCX) [file pone.0342986.s001.docx]

Appendix Table 1. Age-standardized incidence rate (per 100,000 population) and incident cases in 204 countries in 1990 and 2021

| Location | Incidence in 1990 | |  | Incidence in 2021 | |
| --- | --- | --- | --- | --- | --- |
|  | Counts (95% UI) | ASIR (per 100,000) |  | Counts (95% UI) | ASIR (per 100,000) |
| Afghanistan | 171.56 (98.48 to 248.86) | 1.73 (0.99 to 2.5) |  | 301.3 (174.28 to 450.67) | 0.97 (0.56 to 1.44) |
| Albania | 23.31 (16.42 to 31.52) | 0.71 (0.5 to 0.95) |  | 23.35 (14.56 to 34.63) | 0.88 (0.55 to 1.3) |
| Algeria | 184.51 (132.36 to 253.42) | 0.73 (0.52 to 1) |  | 262.79 (183.32 to 400.31) | 0.59 (0.41 to 0.91) |
| American Samoa | 0.06 (0.03 to 0.12) | 0.12 (0.06 to 0.25) |  | 0.13 (0.07 to 0.24) | 0.26 (0.15 to 0.49) |
| Andorra | 0.94 (0.6 to 1.39) | 1.73 (1.1 to 2.56) |  | 1.55 (0.89 to 2.33) | 1.81 (1.04 to 2.72) |
| Angola | 113.52 (65.07 to 197) | 1.1 (0.63 to 1.92) |  | 231.83 (139.86 to 369.17) | 0.71 (0.43 to 1.13) |
| Antigua and Barbuda | 0.6 (0.53 to 0.67) | 0.99 (0.88 to 1.11) |  | 1.14 (1.06 to 1.25) | 1.28 (1.19 to 1.4) |
| Argentina | 413.26 (355.45 to 477.35) | 1.25 (1.07 to 1.44) |  | 652.74 (564.2 to 744.23) | 1.43 (1.24 to 1.64) |
| Armenia | 18.21 (12.27 to 25.6) | 0.53 (0.36 to 0.75) |  | 70.19 (49.87 to 97.57) | 2.34 (1.66 to 3.26) |
| Australia | 402.15 (372.74 to 431.25) | 2.39 (2.21 to 2.56) |  | 996.05 (862.64 to 1136.17) | 3.86 (3.34 to 4.41) |
| Austria | 198.6 (183.61 to 213.9) | 2.56 (2.36 to 2.75) |  | 332.59 (285.86 to 382.9) | 3.7 (3.18 to 4.26) |
| Azerbaijan | 22 (13.24 to 33.59) | 0.3 (0.18 to 0.46) |  | 36.02 (19.88 to 59.48) | 0.34 (0.19 to 0.57) |
| Bahamas | 2.9 (2.54 to 3.29) | 1.13 (0.99 to 1.28) |  | 5.85 (4.64 to 7.45) | 1.51 (1.19 to 1.92) |
| Bahrain | 2.47 (1.67 to 3.45) | 0.49 (0.33 to 0.68) |  | 7.43 (4.48 to 10.44) | 0.49 (0.29 to 0.68) |
| Bangladesh | 859.24 (571.74 to 1309.08) | 0.79 (0.52 to 1.2) |  | 1039.7 (601.5 to 1910.5) | 0.63 (0.37 to 1.16) |
| Barbados | 5.73 (5.14 to 6.37) | 2.26 (2.03 to 2.51) |  | 9.77 (7.78 to 12.16) | 3.27 (2.6 to 4.07) |
| Belarus | 185.58 (160.72 to 213.66) | 1.78 (1.54 to 2.05) |  | 199.58 (156.79 to 246.5) | 2.14 (1.68 to 2.64) |
| Belgium | 216.96 (189.11 to 248.74) | 2.17 (1.9 to 2.49) |  | 460.72 (392.03 to 527.37) | 4.02 (3.42 to 4.6) |
| Belize | 0.84 (0.74 to 0.94) | 0.45 (0.4 to 0.5) |  | 1.97 (1.73 to 2.24) | 0.46 (0.4 to 0.52) |
| Benin | 26.29 (14.78 to 43.01) | 0.54 (0.3 to 0.89) |  | 54.64 (33.98 to 94.41) | 0.4 (0.25 to 0.7) |
| Bermuda | 1.61 (1.32 to 2) | 2.71 (2.23 to 3.37) |  | 2.81 (2.25 to 3.66) | 4.43 (3.55 to 5.76) |
| Bhutan | 4.53 (3.02 to 6.69) | 0.72 (0.48 to 1.06) |  | 4.89 (2.52 to 9.51) | 0.65 (0.33 to 1.26) |
| Bolivia (Plurinational State of) | 75.28 (51.63 to 104.36) | 1.18 (0.81 to 1.64) |  | 115.27 (77.67 to 164.01) | 0.98 (0.66 to 1.39) |
| Bosnia and Herzegovina | 28.38 (18.26 to 41.35) | 0.63 (0.41 to 0.92) |  | 33.02 (21.35 to 48.54) | 1 (0.65 to 1.47) |
| Botswana | 10.38 (6.73 to 14.8) | 0.79 (0.51 to 1.12) |  | 23.91 (15.48 to 35.52) | 1 (0.65 to 1.48) |
| Brazil | 1055.25 (963.45 to 1164) | 0.71 (0.65 to 0.78) |  | 3005.86 (2779.5 to 3242.98) | 1.36 (1.26 to 1.47) |
| Brunei Darussalam | 4.64 (3.11 to 7.24) | 1.79 (1.2 to 2.79) |  | 7.6 (5.47 to 10.88) | 1.69 (1.21 to 2.41) |
| Bulgaria | 57.84 (42.96 to 74.02) | 0.67 (0.49 to 0.85) |  | 102.35 (75.37 to 135.34) | 1.51 (1.11 to 1.99) |
| Burkina Faso | 63.86 (39.76 to 101.58) | 0.67 (0.42 to 1.07) |  | 112.11 (72.36 to 179.06) | 0.49 (0.32 to 0.79) |
| Burundi | 108.61 (70.64 to 171.63) | 1.96 (1.27 to 3.09) |  | 141.31 (93.74 to 221.51) | 1.07 (0.71 to 1.68) |
| Cabo Verde | 0.96 (0.6 to 1.68) | 0.27 (0.17 to 0.47) |  | 2.11 (1.35 to 3.37) | 0.38 (0.24 to 0.6) |
| Cambodia | 73.3 (51.11 to 103.48) | 0.71 (0.5 to 1.01) |  | 127.8 (85.81 to 190.14) | 0.75 (0.5 to 1.12) |
| Cameroon | 54.38 (34.48 to 85.53) | 0.52 (0.33 to 0.82) |  | 158.94 (96.45 to 248.9) | 0.5 (0.3 to 0.78) |
| Canada | 704.44 (643.29 to 766.46) | 2.58 (2.36 to 2.81) |  | 1436.7 (1250.1 to 1672.47) | 3.83 (3.34 to 4.46) |
| Central African Republic | 28.18 (18.39 to 45.95) | 1.03 (0.67 to 1.68) |  | 53.94 (33.72 to 87.54) | 0.98 (0.61 to 1.6) |
| Chad | 32.07 (19.1 to 54.37) | 0.53 (0.32 to 0.9) |  | 91.96 (56.76 to 149.38) | 0.52 (0.32 to 0.84) |
| Chile | 143.12 (121.72 to 167.72) | 1.08 (0.92 to 1.26) |  | 355.26 (294.49 to 419.79) | 1.89 (1.57 to 2.23) |
| China | 5803.83 (4084.97 to 7529.94) | 0.49 (0.35 to 0.64) |  | 9226.99 (6351.77 to 13045.46) | 0.65 (0.45 to 0.92) |
| Colombia | 264.24 (231.73 to 298.68) | 0.81 (0.71 to 0.92) |  | 801.56 (660.05 to 972.68) | 1.63 (1.35 to 1.98) |
| Comoros | 7.75 (4.83 to 12.61) | 1.67 (1.04 to 2.73) |  | 11.32 (6.69 to 18.87) | 1.52 (0.9 to 2.53) |
| Congo | 20.51 (13.32 to 33.32) | 0.85 (0.55 to 1.39) |  | 42.63 (27.37 to 67.29) | 0.79 (0.51 to 1.25) |
| Cook Islands | 0.02 (0.01 to 0.04) | 0.13 (0.07 to 0.21) |  | 0.03 (0.02 to 0.05) | 0.17 (0.1 to 0.27) |
| Costa Rica | 41.19 (34.89 to 48.48) | 1.35 (1.15 to 1.59) |  | 104.04 (85.61 to 124.17) | 2.19 (1.8 to 2.62) |
| Croatia | 74.28 (60.15 to 89.94) | 1.53 (1.24 to 1.85) |  | 117.27 (92 to 143.78) | 2.79 (2.19 to 3.42) |
| Cuba | 168.39 (142.26 to 202.75) | 1.55 (1.31 to 1.87) |  | 210.47 (171.18 to 255.9) | 1.87 (1.52 to 2.27) |
| Cyprus | 15.63 (11.32 to 21.51) | 2.01 (1.45 to 2.76) |  | 26.01 (16.14 to 34.6) | 1.92 (1.19 to 2.55) |
| Czechia | 198.42 (171.13 to 233.68) | 1.93 (1.66 to 2.27) |  | 327.82 (247.43 to 419.39) | 3.08 (2.33 to 3.94) |
| Saint Helena | 46.16 (29.57 to 67.68) | 0.38 (0.24 to 0.55) |  | 108.89 (59.37 to 170.41) | 0.39 (0.21 to 0.61) |
| Democratic People's Republic of Korea | 132.11 (83.42 to 203.83) | 0.64 (0.41 to 0.99) |  | 244.54 (161.8 to 377.37) | 0.93 (0.61 to 1.43) |
| Democratic Republic of the Congo | 318.88 (194.05 to 507.77) | 0.84 (0.51 to 1.33) |  | 560.08 (359.91 to 894.18) | 0.62 (0.4 to 0.99) |
| Denmark | 129.44 (113.45 to 146.63) | 2.52 (2.21 to 2.85) |  | 190.76 (160.61 to 221.49) | 3.26 (2.74 to 3.79) |
| Djibouti | 4.09 (2.37 to 6.94) | 0.99 (0.57 to 1.67) |  | 17.09 (10.05 to 30.59) | 1.36 (0.8 to 2.43) |
| Dominica | 0.7 (0.5 to 1.02) | 0.97 (0.69 to 1.41) |  | 0.9 (0.62 to 1.24) | 1.33 (0.92 to 1.85) |
| Dominican Republic | 36.52 (24.53 to 50.31) | 0.51 (0.34 to 0.7) |  | 65.77 (34.98 to 100.43) | 0.6 (0.32 to 0.91) |
| Ecuador | 51.84 (43.99 to 60.44) | 0.52 (0.44 to 0.61) |  | 229.34 (180.24 to 290.04) | 1.27 (1 to 1.61) |
| Egypt | 224.96 (148.05 to 299.93) | 0.41 (0.27 to 0.54) |  | 258.73 (168.64 to 431.55) | 0.24 (0.16 to 0.41) |
| El Salvador | 35.04 (24.79 to 46.55) | 0.66 (0.47 to 0.88) |  | 59.77 (35.32 to 78.52) | 0.93 (0.55 to 1.22) |
| Equatorial Guinea | 3.99 (2.44 to 6.58) | 0.94 (0.58 to 1.56) |  | 9.29 (5.3 to 15.9) | 0.61 (0.35 to 1.05) |
| Eritrea | 52.32 (33.57 to 88.4) | 1.54 (0.99 to 2.6) |  | 99.39 (58.76 to 168.8) | 1.51 (0.89 to 2.56) |
| Estonia | 16.32 (12.99 to 20.61) | 1.04 (0.83 to 1.31) |  | 29.05 (22.1 to 37.28) | 2.22 (1.69 to 2.84) |
| Eswatini | 6.78 (4.85 to 9.95) | 0.84 (0.6 to 1.23) |  | 15.93 (9.06 to 23.74) | 1.38 (0.78 to 2.05) |
| Ethiopia | 1483.04 (1012.72 to 2295.1) | 2.93 (2 to 4.54) |  | 1604.2 (1113.83 to 2549.72) | 1.47 (1.02 to 2.34) |
| Fiji | 2.6 (1.29 to 5.11) | 0.34 (0.17 to 0.67) |  | 3.52 (1.59 to 7.44) | 0.38 (0.17 to 0.8) |
| Finland | 129.37 (113.43 to 145.34) | 2.58 (2.26 to 2.9) |  | 233.81 (195.48 to 274.03) | 4.22 (3.53 to 4.95) |
| France | 1331.55 (1216.08 to 1428.57) | 2.3 (2.1 to 2.47) |  | 2684.41 (2207.26 to 3169.61) | 4.04 (3.32 to 4.77) |
| Gabon | 8.41 (5.62 to 13.32) | 0.86 (0.57 to 1.35) |  | 15.21 (8.86 to 25.27) | 0.84 (0.49 to 1.39) |
| Gambia | 5.02 (3.2 to 7.61) | 0.51 (0.33 to 0.78) |  | 11.66 (7.22 to 17.78) | 0.49 (0.3 to 0.74) |
| Georgia | 2.61 (1.88 to 3.65) | 0.05 (0.03 to 0.07) |  | 92.56 (65.71 to 124.07) | 2.57 (1.82 to 3.44) |
| Germany | 1873.31 (1642.8 to 2083.94) | 2.34 (2.06 to 2.61) |  | 4078.1 (3554.56 to 4540.24) | 4.78 (4.16 to 5.32) |
| Ghana | 59.36 (38.24 to 92.21) | 0.4 (0.26 to 0.62) |  | 142.07 (93.6 to 223.66) | 0.41 (0.27 to 0.65) |
| Greece | 153.09 (143.31 to 163.45) | 1.47 (1.38 to 1.57) |  | 364.6 (330.49 to 400.01) | 3.58 (3.25 to 3.93) |
| Greenland | 1.06 (0.62 to 1.41) | 1.92 (1.12 to 2.53) |  | 0.76 (0.45 to 1.02) | 1.36 (0.8 to 1.82) |
| Grenada | 1.08 (0.84 to 1.33) | 1.24 (0.96 to 1.52) |  | 1.84 (1.52 to 2.23) | 1.79 (1.48 to 2.18) |
| Guam | 0.32 (0.21 to 0.43) | 0.24 (0.15 to 0.32) |  | 0.35 (0.25 to 0.59) | 0.22 (0.16 to 0.37) |
| Guatemala | 29.03 (23.08 to 41.97) | 0.35 (0.28 to 0.5) |  | 76.49 (64.4 to 90.51) | 0.49 (0.41 to 0.57) |
| Guinea | 57.95 (33.99 to 95.27) | 0.97 (0.57 to 1.59) |  | 84.58 (51.4 to 138.05) | 0.63 (0.38 to 1.03) |
| Guinea-Bissau | 7.36 (4.52 to 11.9) | 0.73 (0.45 to 1.18) |  | 10.37 (6.72 to 15.59) | 0.5 (0.33 to 0.76) |
| Guyana | 0.3 (0.24 to 0.37) | 0.04 (0.03 to 0.05) |  | 6.3 (4.52 to 8.54) | 0.82 (0.59 to 1.12) |
| Haiti | 135.01 (82 to 199.21) | 2.12 (1.29 to 3.12) |  | 205.34 (126.29 to 302.38) | 1.6 (0.98 to 2.35) |
| Honduras | 37.7 (26.64 to 54.52) | 0.8 (0.57 to 1.16) |  | 98.15 (64.15 to 142.65) | 0.97 (0.63 to 1.41) |
| Hungary | 222.06 (194.8 to 248.75) | 2.14 (1.87 to 2.39) |  | 347.64 (275.15 to 443.9) | 3.62 (2.87 to 4.63) |
| Iceland | 5.03 (4.52 to 5.56) | 1.98 (1.78 to 2.19) |  | 11.84 (10.14 to 13.83) | 3.38 (2.9 to 3.95) |
| India | 6220 (3590.55 to 7864.94) | 0.73 (0.42 to 0.92) |  | 9673.02 (6652.52 to 12829.38) | 0.68 (0.47 to 0.91) |
| Indonesia | 877.87 (630.96 to 1237.28) | 0.47 (0.34 to 0.67) |  | 1541.81 (1130.14 to 2375.06) | 0.55 (0.41 to 0.85) |
| Iran (Islamic Republic of) | 599.32 (459.75 to 918.76) | 1.05 (0.81 to 1.61) |  | 763.44 (597.43 to 1135.69) | 0.89 (0.7 to 1.33) |
| Iraq | 105.9 (72.58 to 153.92) | 0.58 (0.39 to 0.84) |  | 197.5 (128.8 to 280.13) | 0.48 (0.31 to 0.68) |
| Ireland | 80.33 (72.59 to 89.32) | 2.23 (2.02 to 2.48) |  | 145.09 (120.65 to 169.21) | 2.94 (2.44 to 3.42) |
| Israel | 109.32 (93.13 to 127.11) | 2.2 (1.88 to 2.56) |  | 281.78 (239.95 to 329.58) | 2.94 (2.5 to 3.44) |
| Italy | 980.85 (921 to 1037.1) | 1.73 (1.62 to 1.83) |  | 2372.56 (2081.09 to 2627.75) | 3.97 (3.48 to 4.39) |
| Jamaica | 26.22 (21.99 to 31.67) | 1.11 (0.93 to 1.34) |  | 45.76 (33.92 to 60.32) | 1.63 (1.21 to 2.15) |
| Japan | 1543.24 (1478.91 to 1596.57) | 1.23 (1.18 to 1.27) |  | 3132.1 (2771.29 to 3412.58) | 2.45 (2.17 to 2.67) |
| Jordan | 19.45 (13.13 to 26.8) | 0.52 (0.35 to 0.72) |  | 46.09 (31.09 to 69.79) | 0.37 (0.25 to 0.57) |
| Kazakhstan | 193.76 (150.32 to 252.82) | 1.18 (0.92 to 1.54) |  | 281.23 (206.63 to 375.28) | 1.48 (1.09 to 1.98) |
| Kenya | 190.64 (132.47 to 251.23) | 0.82 (0.57 to 1.09) |  | 454.75 (327.43 to 619.12) | 0.91 (0.65 to 1.24) |
| Kiribati | 0.24 (0.15 to 0.35) | 0.32 (0.2 to 0.47) |  | 0.32 (0.2 to 0.5) | 0.26 (0.16 to 0.41) |
| Kuwait | 12.29 (10.29 to 14.56) | 0.72 (0.6 to 0.85) |  | 19.01 (15.19 to 23.75) | 0.41 (0.33 to 0.51) |
| Kyrgyzstan | 37.37 (28.42 to 49.65) | 0.84 (0.64 to 1.11) |  | 60.63 (45.24 to 80.79) | 0.88 (0.66 to 1.18) |
| Lao People's Democratic Republic | 29.47 (18.31 to 44.73) | 0.71 (0.44 to 1.07) |  | 43.3 (28.71 to 67.55) | 0.59 (0.39 to 0.92) |
| Latvia | 49.51 (38.2 to 63.51) | 1.86 (1.44 to 2.39) |  | 54.37 (41.02 to 71.12) | 2.91 (2.19 to 3.8) |
| Lebanon | 40.96 (28.55 to 60.95) | 1.37 (0.95 to 2.04) |  | 60.88 (41.67 to 94.86) | 1.1 (0.75 to 1.71) |
| Lesotho | 10.75 (7.23 to 15.77) | 0.7 (0.47 to 1.03) |  | 27.14 (17.96 to 38.38) | 1.45 (0.96 to 2.05) |
| Liberia | 17.02 (10.38 to 28.06) | 0.69 (0.42 to 1.14) |  | 22.32 (14 to 35.02) | 0.41 (0.26 to 0.64) |
| Libya | 61.33 (36.6 to 105.21) | 1.46 (0.87 to 2.5) |  | 155.2 (93.08 to 259.79) | 2.26 (1.35 to 3.78) |
| Lithuania | 45.94 (36.14 to 58.33) | 1.25 (0.98 to 1.59) |  | 81.37 (62.03 to 102.42) | 2.98 (2.27 to 3.75) |
| Luxembourg | 9.19 (8.63 to 9.8) | 2.41 (2.26 to 2.57) |  | 20.14 (17.5 to 22.88) | 3.13 (2.72 to 3.55) |
| Madagascar | 149.02 (95.53 to 248.87) | 1.25 (0.8 to 2.09) |  | 297.93 (185.32 to 484.67) | 1.04 (0.65 to 1.7) |
| Malawi | 183.04 (120.92 to 260.7) | 1.87 (1.23 to 2.66) |  | 234.08 (149.59 to 345.57) | 1.2 (0.77 to 1.78) |
| Malaysia | 106.67 (78.18 to 158.7) | 0.6 (0.44 to 0.9) |  | 242.26 (178.6 to 355.13) | 0.76 (0.56 to 1.12) |
| Maldives | 0.83 (0.5 to 1.16) | 0.37 (0.22 to 0.52) |  | 0.93 (0.6 to 1.31) | 0.18 (0.12 to 0.25) |
| Mali | 41.6 (22.27 to 68.38) | 0.48 (0.26 to 0.79) |  | 64.72 (31.54 to 106.41) | 0.27 (0.13 to 0.44) |
| Malta | 7.82 (6.98 to 8.76) | 2.11 (1.88 to 2.36) |  | 19.27 (15.8 to 23.23) | 4.36 (3.57 to 5.25) |
| Marshall Islands | 0.05 (0.03 to 0.07) | 0.11 (0.06 to 0.16) |  | 0.1 (0.06 to 0.15) | 0.18 (0.1 to 0.27) |
| Mauritania | 9.05 (5.76 to 14.77) | 0.44 (0.28 to 0.72) |  | 15.58 (9.71 to 24.64) | 0.35 (0.22 to 0.56) |
| Mauritius | 3.51 (3.19 to 3.83) | 0.32 (0.29 to 0.35) |  | 15.39 (13.8 to 16.81) | 1.21 (1.08 to 1.32) |
| Mexico | 648.93 (604.6 to 701.23) | 0.76 (0.71 to 0.82) |  | 1861.95 (1651.71 to 2073.84) | 1.44 (1.28 to 1.6) |
| Micronesia (Federated States of) | 0.14 (0.07 to 0.21) | 0.13 (0.07 to 0.2) |  | 0.18 (0.11 to 0.28) | 0.18 (0.1 to 0.27) |
| Monaco | 0.14 (0.05 to 0.26) | 0.47 (0.15 to 0.85) |  | 0.21 (0.07 to 0.36) | 0.56 (0.18 to 0.94) |
| Mongolia | 9.11 (5.1 to 16.73) | 0.42 (0.24 to 0.78) |  | 20.29 (13.25 to 28.19) | 0.61 (0.4 to 0.84) |
| Montenegro | 2.59 (1.34 to 3.49) | 0.41 (0.21 to 0.56) |  | 3.3 (1.43 to 4.8) | 0.53 (0.23 to 0.78) |
| Morocco | 118.67 (78.45 to 166.91) | 0.47 (0.31 to 0.66) |  | 185.85 (111.41 to 265.54) | 0.5 (0.3 to 0.71) |
| Mozambique | 238.07 (152.64 to 385.2) | 1.78 (1.14 to 2.88) |  | 421.4 (250.56 to 670.91) | 1.36 (0.81 to 2.16) |
| Myanmar | 331.76 (216 to 496.13) | 0.82 (0.53 to 1.23) |  | 362.1 (245.33 to 553.2) | 0.64 (0.43 to 0.98) |
| Namibia | 11.24 (8.11 to 16.58) | 0.8 (0.58 to 1.18) |  | 26.57 (16.77 to 43.81) | 1.09 (0.69 to 1.8) |
| Nauru | 0.02 (0.01 to 0.03) | 0.19 (0.1 to 0.29) |  | 0.02 (0.01 to 0.03) | 0.22 (0.13 to 0.32) |
| Nepal | 134.57 (86.68 to 216.42) | 0.69 (0.45 to 1.11) |  | 193.02 (106.66 to 361.18) | 0.62 (0.34 to 1.16) |
| Netherlands | 342.05 (307.75 to 379.37) | 2.29 (2.06 to 2.54) |  | 528.55 (438.61 to 619.09) | 3.07 (2.55 to 3.6) |
| New Zealand | 69.91 (60.24 to 79.88) | 2.05 (1.76 to 2.34) |  | 130.81 (110.8 to 152.47) | 2.53 (2.14 to 2.95) |
| Nicaragua | 23.63 (18.29 to 32.99) | 0.61 (0.47 to 0.85) |  | 44.45 (29.16 to 59.24) | 0.67 (0.44 to 0.89) |
| Niger | 68.57 (37.75 to 111.5) | 0.85 (0.47 to 1.39) |  | 99.97 (60.07 to 163.33) | 0.4 (0.24 to 0.65) |
| Nigeria | 997.88 (652.91 to 1759.6) | 1.11 (0.73 to 1.95) |  | 1776.43 (1194.26 to 2797.78) | 0.77 (0.52 to 1.21) |
| Niue | 0 (0 to 0.01) | 0.18 (0.1 to 0.29) |  | 0 (0 to 0.01) | 0.27 (0.17 to 0.42) |
| North Macedonia | 17.64 (13.47 to 23.99) | 0.89 (0.68 to 1.2) |  | 21.91 (13.45 to 28.74) | 1.01 (0.62 to 1.32) |
| Northern Mariana Islands | 0.01 (0 to 0.02) | 0.02 (0.01 to 0.04) |  | 0.01 (0.01 to 0.02) | 0.03 (0.01 to 0.04) |
| Norway | 85.01 (79.21 to 90.54) | 2 (1.87 to 2.13) |  | 198.45 (177.97 to 220.88) | 3.66 (3.28 to 4.08) |
| Oman | 6.71 (4.4 to 10.04) | 0.34 (0.22 to 0.51) |  | 11.86 (7.92 to 17.62) | 0.25 (0.17 to 0.37) |
| Pakistan | 1314.42 (955.89 to 1990.54) | 1.18 (0.86 to 1.79) |  | 2641.34 (1734.85 to 4610.05) | 1.12 (0.74 to 1.96) |
| Palau | 0.01 (0 to 0.01) | 0.04 (0.02 to 0.08) |  | 0.01 (0 to 0.01) | 0.05 (0.02 to 0.08) |
| Palestine | 5.66 (3.36 to 8.26) | 0.28 (0.16 to 0.4) |  | 10.93 (6.07 to 15.44) | 0.21 (0.12 to 0.3) |
| Panama | 17.18 (15.46 to 18.94) | 0.72 (0.65 to 0.79) |  | 51.7 (41.96 to 62) | 1.2 (0.98 to 1.44) |
| Papua New Guinea | 5.45 (2.68 to 8.53) | 0.13 (0.07 to 0.21) |  | 12.24 (7.47 to 19.1) | 0.12 (0.07 to 0.18) |
| Paraguay | 24.51 (16.75 to 34.66) | 0.61 (0.41 to 0.86) |  | 63.76 (40.78 to 90.99) | 0.89 (0.57 to 1.27) |
| Peru | 234.05 (142.22 to 325.5) | 1.08 (0.66 to 1.5) |  | 302.07 (200.19 to 442.12) | 0.83 (0.55 to 1.22) |
| Philippines | 378.72 (251.61 to 451.84) | 0.6 (0.4 to 0.72) |  | 679.34 (446.24 to 843.39) | 0.6 (0.39 to 0.74) |
| Poland | 409.13 (380.88 to 436.2) | 1.07 (1 to 1.14) |  | 1241.35 (1113.19 to 1362.28) | 3.25 (2.91 to 3.56) |
| Portugal | 206.25 (182.29 to 232.5) | 2.03 (1.8 to 2.29) |  | 400.16 (342.03 to 462.14) | 3.77 (3.22 to 4.36) |
| Puerto Rico | 60.58 (51.78 to 70.63) | 1.68 (1.43 to 1.96) |  | 69.71 (56.45 to 84.33) | 2.12 (1.71 to 2.56) |
| Qatar | 1.4 (0.78 to 2.04) | 0.32 (0.18 to 0.46) |  | 6.43 (3.43 to 9.88) | 0.22 (0.12 to 0.33) |
| Republic of Korea | 390.41 (288.97 to 592.81) | 0.88 (0.65 to 1.34) |  | 626.09 (306.26 to 864.48) | 1.21 (0.59 to 1.68) |
| Republic of Moldova | 31.08 (27.1 to 35.02) | 0.7 (0.61 to 0.79) |  | 44.9 (38.61 to 51.72) | 1.25 (1.07 to 1.44) |
| Romania | 294.18 (232.65 to 367.1) | 1.26 (1 to 1.57) |  | 449.61 (355.28 to 553.16) | 2.37 (1.88 to 2.92) |
| Russian Federation | 1705.99 (1299.22 to 1929.57) | 1.13 (0.86 to 1.28) |  | 2979.82 (2691.77 to 3245.96) | 2.06 (1.86 to 2.24) |
| Rwanda | 150.71 (98.69 to 243.52) | 2.1 (1.37 to 3.39) |  | 178.72 (107.54 to 296) | 1.35 (0.81 to 2.23) |
| Saint Kitts and Nevis | 0.35 (0.29 to 0.42) | 0.83 (0.69 to 1) |  | 0.53 (0.44 to 0.65) | 0.91 (0.74 to 1.1) |
| Saint Lucia | 1.62 (1.43 to 1.85) | 1.19 (1.05 to 1.36) |  | 2.8 (2.29 to 3.38) | 1.58 (1.29 to 1.9) |
| Saint Vincent and the Grenadines | 1.05 (0.94 to 1.19) | 0.96 (0.86 to 1.09) |  | 2.42 (2.08 to 2.8) | 2.12 (1.82 to 2.46) |
| Samoa | 1.01 (0.24 to 2.74) | 0.6 (0.14 to 1.62) |  | 1.42 (0.3 to 4.13) | 0.66 (0.14 to 1.93) |
| San Marino | 0.66 (0.46 to 1.02) | 2.8 (1.95 to 4.28) |  | 0.73 (0.43 to 1.21) | 2.23 (1.3 to 3.71) |
| Sao Tome and Principe | 0.69 (0.42 to 1.16) | 0.57 (0.35 to 0.96) |  | 0.78 (0.51 to 1.29) | 0.36 (0.24 to 0.6) |
| Saudi Arabia | 103.21 (65.74 to 149.72) | 0.65 (0.41 to 0.94) |  | 274.17 (171.31 to 448.37) | 0.73 (0.45 to 1.19) |
| Senegal | 43.1 (25.84 to 70.06) | 0.56 (0.34 to 0.92) |  | 61.21 (37.91 to 100.54) | 0.39 (0.24 to 0.63) |
| Serbia | 100.9 (68.29 to 136.54) | 1.05 (0.71 to 1.42) |  | 126.78 (77.08 to 176.86) | 1.42 (0.86 to 1.98) |
| Seychelles | 0.51 (0.37 to 0.75) | 0.69 (0.5 to 1.02) |  | 0.75 (0.54 to 1.11) | 0.71 (0.51 to 1.05) |
| Sierra Leone | 29.55 (17.81 to 47.52) | 0.71 (0.43 to 1.14) |  | 41.82 (26.61 to 64.93) | 0.47 (0.3 to 0.73) |
| Singapore | 45.37 (40.11 to 51.44) | 1.49 (1.32 to 1.69) |  | 118.46 (98.91 to 142.51) | 2.07 (1.73 to 2.49) |
| Slovakia | 78.94 (56.6 to 110.51) | 1.49 (1.07 to 2.09) |  | 83.62 (57.35 to 115.69) | 1.54 (1.06 to 2.13) |
| Slovenia | 31.28 (26.23 to 36.66) | 1.59 (1.33 to 1.86) |  | 58.88 (47.18 to 74.18) | 2.84 (2.28 to 3.58) |
| Solomon Islands | 0.38 (0.18 to 0.62) | 0.11 (0.05 to 0.18) |  | 0.94 (0.54 to 1.49) | 0.14 (0.08 to 0.22) |
| Somalia | 105.13 (60.09 to 185.77) | 1.32 (0.76 to 2.34) |  | 249.22 (145.99 to 431.9) | 1.15 (0.68 to 2) |
| South Africa | 254.78 (167.29 to 316.07) | 0.69 (0.45 to 0.85) |  | 553.88 (327.24 to 687.47) | 0.97 (0.58 to 1.21) |
| South Sudan | 86.98 (51.32 to 156.12) | 1.48 (0.87 to 2.66) |  | 166.42 (99 to 281.72) | 1.72 (1.02 to 2.91) |
| Spain | 855.63 (791.5 to 922.81) | 2.21 (2.04 to 2.38) |  | 1665.72 (1415.56 to 1918.8) | 3.66 (3.11 to 4.21) |
| Sri Lanka | 98.96 (74.4 to 144.83) | 0.58 (0.43 to 0.85) |  | 141.83 (86.79 to 211.66) | 0.64 (0.39 to 0.95) |
| Sudan | 262.9 (165.06 to 393.9) | 1.31 (0.82 to 1.97) |  | 297.17 (183.38 to 461.17) | 0.68 (0.42 to 1.06) |
| Suriname | 4.41 (3.14 to 6.47) | 1.14 (0.81 to 1.67) |  | 7.68 (5.23 to 11.08) | 1.33 (0.9 to 1.91) |
| Sweden | 282.87 (252.9 to 313.42) | 3.29 (2.94 to 3.65) |  | 454.75 (380.51 to 534.53) | 4.38 (3.67 to 5.15) |
| Switzerland | 184.58 (160.23 to 212.95) | 2.69 (2.33 to 3.1) |  | 326.23 (268.64 to 391.92) | 3.66 (3.01 to 4.39) |
| Syrian Arab Republic | 85.98 (57.28 to 117.21) | 0.68 (0.45 to 0.92) |  | 115.4 (69.58 to 166.57) | 0.82 (0.5 to 1.19) |
| Taiwan (Province of China) | 233.06 (215.33 to 252.88) | 1.14 (1.06 to 1.24) |  | 393.4 (327.35 to 458.63) | 1.66 (1.38 to 1.94) |
| Tajikistan | 21.05 (13.71 to 30.91) | 0.39 (0.26 to 0.58) |  | 32.14 (20.21 to 48.48) | 0.32 (0.2 to 0.48) |
| Thailand | 336.48 (220.96 to 442.33) | 0.59 (0.39 to 0.78) |  | 598.66 (427.08 to 884.52) | 0.9 (0.64 to 1.33) |
| Timor-Leste | 3.93 (2.52 to 5.87) | 0.5 (0.32 to 0.75) |  | 6.14 (4.15 to 9.22) | 0.44 (0.3 to 0.66) |
| Togo | 16.39 (9.79 to 25.86) | 0.45 (0.27 to 0.71) |  | 36.24 (22.3 to 56.59) | 0.43 (0.27 to 0.68) |
| Tokelau | 0 (0 to 0) | 0.2 (0.11 to 0.3) |  | 0 (0 to 0.01) | 0.25 (0.16 to 0.41) |
| Tonga | 0.1 (0.06 to 0.15) | 0.1 (0.06 to 0.15) |  | 0.12 (0.07 to 0.19) | 0.11 (0.07 to 0.18) |
| Trinidad and Tobago | 14.66 (13.13 to 16.65) | 1.22 (1.09 to 1.38) |  | 25.66 (19.41 to 32.75) | 1.84 (1.39 to 2.35) |
| Tunisia | 71.91 (50.69 to 110.46) | 0.86 (0.61 to 1.32) |  | 124.19 (82.56 to 193.21) | 1.05 (0.7 to 1.63) |
| Turkey | 724.87 (518.01 to 1020.19) | 1.26 (0.9 to 1.78) |  | 1010.84 (716.99 to 1427.09) | 1.21 (0.86 to 1.71) |
| Turkmenistan | 15.71 (10.86 to 23.05) | 0.42 (0.29 to 0.62) |  | 35.81 (24.21 to 54.16) | 0.69 (0.47 to 1.05) |
| Tuvalu | 0.02 (0.01 to 0.03) | 0.21 (0.12 to 0.3) |  | 0.02 (0.01 to 0.03) | 0.18 (0.11 to 0.26) |
| Uganda | 347.4 (244.38 to 496.17) | 2.01 (1.41 to 2.87) |  | 690.88 (445.2 to 1119.31) | 1.6 (1.03 to 2.58) |
| Ukraine | 801.46 (671.94 to 955.53) | 1.52 (1.27 to 1.81) |  | 806.26 (592.79 to 1064.29) | 1.87 (1.38 to 2.47) |
| United Arab Emirates | 13.92 (9.48 to 20.34) | 0.74 (0.51 to 1.09) |  | 52.05 (36.12 to 71.98) | 0.54 (0.37 to 0.75) |
| United Kingdom | 1431.44 (1378.33 to 1478.65) | 2.5 (2.41 to 2.58) |  | 2584.43 (2405.25 to 2730.44) | 3.81 (3.55 to 4.02) |
| United Republic of Tanzania | 411.23 (260.75 to 674.27) | 1.59 (1.01 to 2.61) |  | 729.07 (447.04 to 1199.29) | 1.25 (0.76 to 2.05) |
| United States of America | 7884.45 (7526.29 to 8184.14) | 3.1 (2.96 to 3.22) |  | 12631.13 (11737.64 to 13277.6) | 3.8 (3.53 to 3.99) |
| United States Virgin Islands | 1.39 (0.98 to 2.1) | 1.31 (0.92 to 1.98) |  | 1.08 (0.75 to 1.62) | 1.26 (0.87 to 1.89) |
| Uruguay | 58.12 (49 to 69.31) | 1.85 (1.56 to 2.21) |  | 89.08 (74.19 to 106.8) | 2.62 (2.18 to 3.14) |
| Uzbekistan | 26.68 (18.68 to 36.91) | 0.13 (0.09 to 0.18) |  | 168.93 (121.88 to 225.16) | 0.49 (0.36 to 0.66) |
| Vanuatu | 0.15 (0.08 to 0.24) | 0.1 (0.05 to 0.16) |  | 0.38 (0.22 to 0.57) | 0.12 (0.07 to 0.18) |
| Venezuela (Bolivarian Republic of) | 124.06 (100.37 to 144.31) | 0.66 (0.53 to 0.77) |  | 342.95 (253.94 to 445.21) | 1.29 (0.95 to 1.67) |
| Viet Nam | 455.62 (317.98 to 685.35) | 0.67 (0.47 to 1) |  | 1019.92 (679.98 to 1638.76) | 1.02 (0.68 to 1.63) |
| Yemen | 122.91 (74.09 to 175.8) | 0.9 (0.54 to 1.29) |  | 200.88 (117.36 to 289.27) | 0.6 (0.35 to 0.86) |
| Zambia | 132.48 (87.3 to 218.98) | 1.67 (1.1 to 2.76) |  | 255.98 (141.33 to 482.18) | 1.31 (0.72 to 2.47) |
| Zimbabwe | 69.94 (48.58 to 104.79) | 0.68 (0.47 to 1.01) |  | 199.96 (126.31 to 319.14) | 1.28 (0.81 to 2.05) |

ASIR, age-standard incidence rate. UI, uncertainty interval.
