# Supplementary material for: Global, regional, and national burden of soft tissue and other extraosseous sarcomas, 1990–2021: A Systematic analysis for the global burden of disease study 2021
Source: PLoS One. 2026 Mar 9;21(3):e0342986. doi: 10.1371/journal.pone.0342986 (PMC12970919; doi:10.1371/journal.pone.0342986)
Supplement: S2 Appendix — Age-standardized prevalence (per 100,000 population) and prevalent cases in 204 countries in 1990 and 2021. (DOCX) [file pone.0342986.s002.docx]

Appendix Table 2. Age-standardized prevalence rate (per 100,000 population) and prevalent cases in 204 countries in 1990 and 2021

| Location | Prevalence in 1990 | |  | Prevalence in 2021 | |
| --- | --- | --- | --- | --- | --- |
|  | Counts (95% UI) | ASPR (per 100,000) |  | Counts (95% UI) | ASPR (per 100,000) |
| Afghanistan | 858.18 (487.76 to 1254.84) | 8.63 (4.91 to 12.62) |  | 1600.63 (922.92 to 2412.39) | 5.13 (7.73 to 2.96) |
| Albania | 124.06 (86.89 to 165.12) | 3.75 (2.63 to 5) |  | 110.75 (68.52 to 167.59) | 4.15 (6.28 to 2.57) |
| Algeria | 989.08 (715.86 to 1369.76) | 3.91 (2.83 to 5.42) |  | 1357.81 (933.39 to 2106.17) | 3.07 (4.77 to 2.11) |
| American Samoa | 0.3 (0.14 to 0.65) | 0.61 (0.28 to 1.34) |  | 0.65 (0.36 to 1.23) | 1.3 (2.47 to 0.73) |
| Andorra | 4.88 (3.1 to 7.23) | 8.97 (5.71 to 13.31) |  | 7.69 (4.4 to 11.51) | 8.98 (13.44 to 5.14) |
| Angola | 612.77 (346.4 to 1079.58) | 5.96 (3.37 to 10.51) |  | 1235.39 (738.18 to 1979.92) | 3.78 (6.05 to 2.26) |
| Antigua and Barbuda | 3.02 (2.69 to 3.39) | 5.02 (4.47 to 5.63) |  | 5.81 (5.37 to 6.38) | 6.5 (7.14 to 6.01) |
| Argentina | 2086.87 (1788.51 to 2419.4) | 6.3 (5.4 to 7.31) |  | 3271.17 (2823.55 to 3745.78) | 7.19 (8.23 to 6.21) |
| Armenia | 92.5 (62.19 to 130.66) | 2.7 (1.82 to 3.82) |  | 340.68 (241.51 to 473.29) | 11.37 (15.8 to 8.06) |
| Australia | 2016.63 (1861.87 to 2165.08) | 11.96 (11.04 to 12.84) |  | 4980.97 (4312.02 to 5690.25) | 19.31 (22.06 to 16.72) |
| Austria | 981.8 (910.76 to 1059.89) | 12.64 (11.72 to 13.64) |  | 1638.77 (1416.48 to 1883.41) | 18.24 (20.97 to 15.77) |
| Azerbaijan | 114.43 (69.29 to 174.15) | 1.56 (0.95 to 2.38) |  | 180.02 (98.54 to 296.36) | 1.71 (2.82 to 0.94) |
| Bahamas | 15.35 (13.39 to 17.46) | 5.98 (5.22 to 6.8) |  | 30.45 (24.01 to 38.76) | 7.85 (9.99 to 6.19) |
| Bahrain | 12.94 (8.66 to 18) | 2.55 (1.71 to 3.55) |  | 39.75 (23.83 to 55.97) | 2.6 (3.66 to 1.56) |
| Bangladesh | 4531.59 (2956.64 to 6887.76) | 4.15 (2.71 to 6.31) |  | 5267.42 (3033.06 to 9686.49) | 3.2 (5.88 to 1.84) |
| Barbados | 28.71 (25.69 to 31.94) | 11.33 (10.14 to 12.6) |  | 47.99 (38.21 to 60.39) | 16.05 (20.2 to 12.78) |
| Belarus | 907.31 (780.69 to 1050.05) | 8.69 (7.47 to 10.05) |  | 960.84 (751.86 to 1187.33) | 10.3 (12.73 to 8.06) |
| Belgium | 1081.16 (939.16 to 1237.14) | 10.83 (9.41 to 12.4) |  | 2275.44 (1934.1 to 2617.89) | 19.84 (22.83 to 16.86) |
| Belize | 4.54 (4.03 to 5.14) | 2.43 (2.15 to 2.75) |  | 10.45 (9.16 to 11.95) | 2.44 (2.78 to 2.14) |
| Benin | 142.55 (78.8 to 233.4) | 2.94 (1.62 to 4.81) |  | 293.87 (179.33 to 513.03) | 2.18 (3.8 to 1.33) |
| Bermuda | 8.02 (6.59 to 9.95) | 13.5 (11.09 to 16.75) |  | 13.79 (11.01 to 17.96) | 21.7 (28.27 to 17.32) |
| Bhutan | 24.04 (15.95 to 35.58) | 3.82 (2.53 to 5.65) |  | 24.06 (12.25 to 47.33) | 3.18 (6.25 to 1.62) |
| Bolivia (Plurinational State of) | 390.09 (265.59 to 550.58) | 6.11 (4.16 to 8.63) |  | 575.25 (383.38 to 820.3) | 4.88 (6.95 to 3.25) |
| Bosnia and Herzegovina | 145.06 (93.33 to 212.06) | 3.22 (2.07 to 4.71) |  | 157.41 (101.78 to 229.98) | 4.77 (6.96 to 3.08) |
| Botswana | 53.5 (34.12 to 76.68) | 4.06 (2.59 to 5.81) |  | 120.95 (77.84 to 179.85) | 5.05 (7.51 to 3.25) |
| Brazil | 5501.96 (5006.1 to 6086.88) | 3.7 (3.37 to 4.1) |  | 15392.63 (14206.8 to 16634.27) | 6.99 (7.55 to 6.45) |
| Brunei Darussalam | 24.73 (16.44 to 38.57) | 9.54 (6.34 to 14.88) |  | 40.11 (28.74 to 57.41) | 8.89 (12.73 to 6.37) |
| Bulgaria | 285.56 (211.94 to 367.17) | 3.29 (2.44 to 4.23) |  | 477.15 (349.23 to 634.02) | 7.03 (9.34 to 5.15) |
| Burkina Faso | 343.37 (210.4 to 553.13) | 3.6 (2.21 to 5.81) |  | 598.42 (382.09 to 964.77) | 2.63 (4.24 to 1.68) |
| Burundi | 560.19 (362.41 to 884.71) | 10.09 (6.53 to 15.93) |  | 738.18 (488.74 to 1150.78) | 5.58 (8.7 to 3.7) |
| Cabo Verde | 4.95 (3.06 to 8.64) | 1.4 (0.86 to 2.44) |  | 10.27 (6.5 to 16.57) | 1.84 (2.96 to 1.16) |
| Cambodia | 385.39 (267.1 to 540.67) | 3.75 (2.6 to 5.26) |  | 649.84 (433.09 to 977.77) | 3.81 (5.74 to 2.54) |
| Cameroon | 291.27 (179.95 to 460.54) | 2.79 (1.72 to 4.41) |  | 840.51 (514.26 to 1336.41) | 2.64 (4.21 to 1.62) |
| Canada | 3569.55 (3257.11 to 3893.08) | 13.1 (11.95 to 14.29) |  | 7229.96 (6316.84 to 8408.74) | 19.3 (22.44 to 16.86) |
| Central African Republic | 145.32 (92.36 to 240.31) | 5.32 (3.38 to 8.8) |  | 278.37 (173.15 to 448.1) | 5.08 (8.17 to 3.16) |
| Chad | 172.32 (100.94 to 294.84) | 2.86 (1.67 to 4.89) |  | 496.72 (306.76 to 810.12) | 2.8 (4.56 to 1.73) |
| Chile | 736.8 (625.72 to 863.75) | 5.55 (4.71 to 6.5) |  | 1807.83 (1503.22 to 2137.58) | 9.62 (11.37 to 8) |
| China | 29307.22 (20529.25 to 37986.49) | 2.49 (1.75 to 3.23) |  | 44376.96 (30777.96 to 62717.51) | 3.12 (4.41 to 2.16) |
| Colombia | 1401.96 (1226 to 1599.33) | 4.31 (3.77 to 4.92) |  | 4149 (3418.67 to 5046.16) | 8.46 (10.29 to 6.97) |
| Comoros | 40.55 (24.99 to 65.39) | 8.77 (5.4 to 14.14) |  | 57.64 (34.13 to 96.62) | 7.74 (12.98 to 4.59) |
| Congo | 105.08 (68.06 to 171.95) | 4.38 (2.83 to 7.16) |  | 220.54 (142 to 346.03) | 4.09 (6.42 to 2.63) |
| Cook Islands | 0.13 (0.07 to 0.2) | 0.66 (0.36 to 1.07) |  | 0.16 (0.09 to 0.25) | 0.88 (1.39 to 0.5) |
| Costa Rica | 212.4 (179.68 to 252.4) | 6.98 (5.91 to 8.3) |  | 532.79 (435.85 to 638.78) | 11.22 (13.45 to 9.18) |
| Croatia | 364.1 (293.31 to 439.97) | 7.49 (6.03 to 9.05) |  | 549.76 (429.48 to 681.75) | 13.06 (16.2 to 10.2) |
| Cuba | 836.89 (706.2 to 1010.91) | 7.72 (6.51 to 9.32) |  | 1046.68 (845.74 to 1276.39) | 9.29 (11.33 to 7.5) |
| Cyprus | 74.4 (54.09 to 102.32) | 9.56 (6.95 to 13.15) |  | 128.46 (78.87 to 173.16) | 9.46 (12.75 to 5.81) |
| Czechia | 946.19 (812.23 to 1112.6) | 9.19 (7.89 to 10.81) |  | 1579.66 (1203.99 to 2014.7) | 14.86 (18.95 to 11.32) |
| Saint Helena | 248.91 (156.92 to 372.73) | 2.04 (1.29 to 3.06) |  | 576.31 (314.02 to 918.52) | 2.07 (3.3 to 1.13) |
| Democratic People's Republic of Korea | 670.13 (421.51 to 1031.9) | 3.25 (2.05 to 5.01) |  | 1213.5 (798.16 to 1873.31) | 4.6 (7.1 to 3.02) |
| Democratic Republic of the Congo | 1705.72 (1027.28 to 2705.62) | 4.47 (2.69 to 7.09) |  | 2941.16 (1903.07 to 4731.45) | 3.27 (5.26 to 2.11) |
| Denmark | 614.54 (536.75 to 697.93) | 11.95 (10.44 to 13.57) |  | 938.16 (791.44 to 1090.76) | 16.03 (18.64 to 13.52) |
| Djibouti | 21.79 (12.51 to 37.02) | 5.26 (3.02 to 8.94) |  | 87.81 (51.71 to 157.61) | 6.98 (12.52 to 4.11) |
| Dominica | 3.5 (2.46 to 5.09) | 4.84 (3.4 to 7.03) |  | 4.5 (3.11 to 6.28) | 6.7 (9.37 to 4.64) |
| Dominican Republic | 201.42 (135.4 to 280.16) | 2.82 (1.89 to 3.92) |  | 344.09 (184.53 to 526.96) | 3.12 (4.78 to 1.68) |
| Ecuador | 269.56 (226.93 to 316.73) | 2.7 (2.27 to 3.17) |  | 1157.46 (912.04 to 1467.9) | 6.41 (8.13 to 5.05) |
| Egypt | 1179.38 (773.83 to 1602.55) | 2.13 (1.4 to 2.9) |  | 1325.51 (855.8 to 2228.49) | 1.25 (2.11 to 0.81) |
| El Salvador | 185.95 (131.99 to 245.73) | 3.5 (2.49 to 4.63) |  | 306.68 (181.38 to 402.2) | 4.75 (6.24 to 2.81) |
| Equatorial Guinea | 20.72 (12.6 to 34.4) | 4.9 (2.98 to 8.14) |  | 49.41 (27.6 to 85.41) | 3.27 (5.65 to 1.82) |
| Eritrea | 269.81 (168.97 to 458.26) | 7.92 (4.96 to 13.45) |  | 515.07 (301.65 to 874.83) | 7.81 (13.26 to 4.57) |
| Estonia | 77.3 (61.48 to 98.38) | 4.93 (3.92 to 6.27) |  | 136.74 (103.7 to 176.67) | 10.43 (13.48 to 7.91) |
| Eswatini | 35.37 (25.13 to 52.07) | 4.39 (3.12 to 6.46) |  | 80.6 (45.81 to 120.55) | 6.98 (10.43 to 3.97) |
| Ethiopia | 7618.47 (5077.18 to 11805.67) | 15.07 (10.04 to 23.35) |  | 8388.61 (5769.97 to 13289.6) | 7.7 (12.2 to 5.3) |
| Fiji | 13.4 (6.63 to 26.22) | 1.77 (0.87 to 3.46) |  | 17.83 (7.99 to 37.71) | 1.93 (4.08 to 0.86) |
| Finland | 624 (546.27 to 701.78) | 12.46 (10.9 to 14.01) |  | 1143.27 (958.73 to 1337.73) | 20.65 (24.16 to 17.32) |
| France | 6641.2 (6040.07 to 7131.33) | 11.5 (10.46 to 12.34) |  | 13119.14 (10869.62 to 15592.15) | 19.76 (23.49 to 16.37) |
| Gabon | 42.25 (28.02 to 67.32) | 4.3 (2.85 to 6.84) |  | 77.7 (44.86 to 129.89) | 4.28 (7.15 to 2.47) |
| Gambia | 27.27 (17.09 to 41.67) | 2.78 (1.74 to 4.25) |  | 60.3 (37.25 to 91.56) | 2.52 (3.82 to 1.56) |
| Georgia | 13.06 (9.37 to 18.25) | 0.24 (0.17 to 0.33) |  | 441.23 (314.21 to 593.22) | 12.23 (16.45 to 8.71) |
| Germany | 9183.04 (8083.93 to 10248.35) | 11.49 (10.11 to 12.82) |  | 19974.83 (17480.47 to 22211.96) | 23.4 (26.02 to 20.48) |
| Ghana | 317.8 (202.05 to 498.35) | 2.12 (1.35 to 3.33) |  | 734.6 (475.53 to 1155.2) | 2.15 (3.37 to 1.39) |
| Greece | 758.58 (709.82 to 810.21) | 7.3 (6.83 to 7.8) |  | 1754.91 (1595.46 to 1916.85) | 17.25 (18.84 to 15.68) |
| Greenland | 5.48 (3.23 to 7.21) | 9.86 (5.81 to 12.98) |  | 3.87 (2.32 to 5.24) | 6.91 (9.34 to 4.13) |
| Grenada | 5.43 (4.19 to 6.78) | 6.24 (4.82 to 7.79) |  | 9.33 (7.66 to 11.44) | 9.09 (11.14 to 7.46) |
| Guam | 1.7 (1.1 to 2.29) | 1.25 (0.8 to 1.67) |  | 1.82 (1.3 to 3.07) | 1.14 (1.93 to 0.82) |
| Guatemala | 153.24 (120.57 to 224.17) | 1.83 (1.44 to 2.67) |  | 397.84 (334.51 to 472.1) | 2.52 (2.99 to 2.12) |
| Guinea | 313.61 (180.15 to 519.5) | 5.23 (3.01 to 8.67) |  | 444.87 (266.8 to 741.85) | 3.31 (5.52 to 1.99) |
| Guinea-Bissau | 38.91 (23.36 to 63.68) | 3.86 (2.32 to 6.32) |  | 53.86 (34.37 to 82.2) | 2.61 (3.98 to 1.67) |
| Guyana | 1.56 (1.23 to 1.96) | 0.2 (0.16 to 0.25) |  | 32.75 (23.57 to 44.5) | 4.28 (5.82 to 3.08) |
| Haiti | 730.63 (442.05 to 1086.14) | 11.45 (6.93 to 17.02) |  | 1101.95 (666.15 to 1634.28) | 8.57 (12.7 to 5.18) |
| Honduras | 201.85 (141.1 to 295.3) | 4.29 (3 to 6.27) |  | 491.25 (318.04 to 724.31) | 4.86 (7.16 to 3.15) |
| Hungary | 1078.38 (946.41 to 1211.52) | 10.37 (9.1 to 11.65) |  | 1688.22 (1337.44 to 2156.18) | 17.59 (22.47 to 13.94) |
| Iceland | 25.07 (22.57 to 27.7) | 9.87 (8.89 to 10.91) |  | 59.86 (51.31 to 69.81) | 17.08 (19.92 to 14.64) |
| India | 31897.2 (18399.78 to 40464.76) | 3.74 (2.16 to 4.74) |  | 47688.92 (32820.05 to 62595.98) | 3.37 (4.43 to 2.32) |
| Indonesia | 4631.03 (3325.93 to 6523.62) | 2.5 (1.8 to 3.53) |  | 7824.36 (5743.6 to 12086.12) | 2.81 (4.33 to 2.06) |
| Iran (Islamic Republic of) | 3230.47 (2480.85 to 4981.13) | 5.66 (4.34 to 8.72) |  | 3936.53 (3068.47 to 5948.13) | 4.61 (6.97 to 3.59) |
| Iraq | 566.33 (387.6 to 819.37) | 3.07 (2.1 to 4.45) |  | 1038.18 (677.16 to 1481.77) | 2.52 (3.59 to 1.64) |
| Ireland | 399.51 (359.14 to 444.81) | 11.09 (9.97 to 12.35) |  | 732.97 (608.81 to 854.52) | 14.83 (17.29 to 12.32) |
| Israel | 561.15 (477.91 to 652.96) | 11.31 (9.63 to 13.16) |  | 1421.85 (1207.35 to 1664.79) | 14.82 (17.35 to 12.58) |
| Italy | 4986.17 (4703.14 to 5272.67) | 8.78 (8.28 to 9.28) |  | 11600.43 (10282.17 to 12795.38) | 19.4 (21.39 to 17.19) |
| Jamaica | 137.1 (114.47 to 167.08) | 5.8 (4.84 to 7.06) |  | 231.72 (170.94 to 306.13) | 8.28 (10.93 to 6.11) |
| Japan | 8028.36 (7723.61 to 8296.87) | 6.38 (6.14 to 6.59) |  | 14877.59 (13346.04 to 16049.81) | 11.65 (12.57 to 10.45) |
| Jordan | 104.53 (71.01 to 145.19) | 2.8 (1.9 to 3.89) |  | 247.27 (166.41 to 374.84) | 2.01 (3.04 to 1.35) |
| Kazakhstan | 982.66 (762.99 to 1294.98) | 5.99 (4.65 to 7.9) |  | 1421.77 (1049.82 to 1894.48) | 7.5 (9.99 to 5.54) |
| Kenya | 1006.15 (698.35 to 1317.86) | 4.35 (3.02 to 5.69) |  | 2323.06 (1665.17 to 3162.74) | 4.64 (6.32 to 3.33) |
| Kiribati | 1.2 (0.76 to 1.81) | 1.62 (1.02 to 2.43) |  | 1.62 (0.98 to 2.55) | 1.33 (2.1 to 0.81) |
| Kuwait | 65.8 (54.93 to 78.22) | 3.83 (3.2 to 4.55) |  | 103.96 (82.97 to 130.1) | 2.24 (2.8 to 1.78) |
| Kyrgyzstan | 191.58 (145.06 to 256.94) | 4.29 (3.25 to 5.76) |  | 316.76 (231.35 to 424.25) | 4.62 (6.18 to 3.37) |
| Lao People's Democratic Republic | 152.81 (93.58 to 233.25) | 3.66 (2.24 to 5.59) |  | 222.58 (146.24 to 347.81) | 3.02 (4.71 to 1.98) |
| Latvia | 236.52 (181.3 to 305.68) | 8.9 (6.82 to 11.5) |  | 255.11 (192.43 to 334.3) | 13.64 (17.87 to 10.29) |
| Lebanon | 209.54 (146.7 to 313.43) | 7 (4.9 to 10.48) |  | 306.7 (209.33 to 473.41) | 5.54 (8.54 to 3.78) |
| Lesotho | 53.92 (36.16 to 79.41) | 3.52 (2.36 to 5.18) |  | 131.74 (86.96 to 186.59) | 7.03 (9.95 to 4.64) |
| Liberia | 92.23 (55.02 to 153.08) | 3.75 (2.24 to 6.22) |  | 118.04 (73.84 to 186.55) | 2.16 (3.42 to 1.35) |
| Libya | 326.02 (190.36 to 562.29) | 7.73 (4.52 to 13.34) |  | 816.86 (484.54 to 1379.14) | 11.89 (20.07 to 7.05) |
| Lithuania | 222.52 (175.15 to 283.77) | 6.06 (4.77 to 7.72) |  | 385.49 (294.13 to 482.25) | 14.13 (17.68 to 10.78) |
| Luxembourg | 46.01 (43.2 to 49.02) | 12.07 (11.33 to 12.86) |  | 99.95 (87.05 to 113.45) | 15.51 (17.61 to 13.51) |
| Madagascar | 787.4 (495.51 to 1326.39) | 6.62 (4.16 to 11.15) |  | 1576.44 (967.99 to 2551.83) | 5.52 (8.94 to 3.39) |
| Malawi | 984.02 (644.65 to 1419.94) | 10.04 (6.57 to 14.48) |  | 1220.31 (769.05 to 1789.15) | 6.27 (9.2 to 3.95) |
| Malaysia | 546.06 (397.77 to 814.95) | 3.09 (2.25 to 4.61) |  | 1222.59 (892.63 to 1790.38) | 3.84 (5.63 to 2.81) |
| Maldives | 4.46 (2.67 to 6.27) | 2.01 (1.2 to 2.82) |  | 4.83 (3.11 to 6.79) | 0.93 (1.31 to 0.6) |
| Mali | 226.41 (121.22 to 380.05) | 2.61 (1.4 to 4.39) |  | 346.88 (169.45 to 577.73) | 1.44 (2.4 to 0.7) |
| Malta | 39.49 (35.15 to 44.24) | 10.65 (9.48 to 11.94) |  | 96.69 (79.22 to 117.23) | 21.86 (26.51 to 17.91) |
| Marshall Islands | 0.26 (0.15 to 0.38) | 0.58 (0.33 to 0.83) |  | 0.51 (0.3 to 0.76) | 0.91 (1.36 to 0.54) |
| Mauritania | 47.05 (28.94 to 77.26) | 2.29 (1.41 to 3.76) |  | 80.23 (49.7 to 126.76) | 1.83 (2.88 to 1.13) |
| Mauritius | 18.18 (16.52 to 19.86) | 1.66 (1.51 to 1.81) |  | 77.26 (69.24 to 84.41) | 6.07 (6.64 to 5.44) |
| Mexico | 3444.65 (3194.62 to 3744.54) | 4.03 (3.74 to 4.39) |  | 9597.1 (8490.32 to 10643.53) | 7.42 (8.23 to 6.57) |
| Micronesia (Federated States of) | 0.69 (0.38 to 1.06) | 0.67 (0.37 to 1.03) |  | 0.92 (0.53 to 1.42) | 0.9 (1.38 to 0.52) |
| Monaco | 0.7 (0.22 to 1.24) | 2.31 (0.72 to 4.09) |  | 1.02 (0.33 to 1.7) | 2.7 (4.5 to 0.87) |
| Mongolia | 47.47 (26.3 to 87.6) | 2.2 (1.22 to 4.06) |  | 103.85 (68.06 to 143.66) | 3.11 (4.31 to 2.04) |
| Montenegro | 13.39 (6.86 to 18.05) | 2.14 (1.1 to 2.88) |  | 15.79 (6.85 to 23.05) | 2.55 (3.73 to 1.11) |
| Morocco | 619.73 (421.62 to 880.8) | 2.44 (1.66 to 3.47) |  | 923.86 (563.71 to 1326.23) | 2.49 (3.57 to 1.52) |
| Mozambique | 1271.16 (804.33 to 2059.56) | 9.51 (6.02 to 15.42) |  | 2169.01 (1277.31 to 3477.51) | 6.98 (11.19 to 4.11) |
| Myanmar | 1734.27 (1111.39 to 2635.71) | 4.29 (2.75 to 6.52) |  | 1860.28 (1258.26 to 2856.06) | 3.3 (5.06 to 2.23) |
| Namibia | 57.55 (41.48 to 84.47) | 4.1 (2.95 to 6.02) |  | 134.87 (85.22 to 222.43) | 5.55 (9.15 to 3.51) |
| Nauru | 0.1 (0.05 to 0.15) | 0.99 (0.52 to 1.47) |  | 0.12 (0.07 to 0.18) | 1.1 (1.64 to 0.64) |
| Nepal | 707.44 (452.31 to 1127.99) | 3.63 (2.32 to 5.79) |  | 957.43 (522.68 to 1806.23) | 3.08 (5.8 to 1.68) |
| Netherlands | 1703.58 (1537.7 to 1890.26) | 11.42 (10.31 to 12.67) |  | 2569.15 (2125.05 to 3007.14) | 14.93 (17.47 to 12.35) |
| New Zealand | 354.49 (306.01 to 404.49) | 10.37 (8.95 to 11.84) |  | 651.81 (554.03 to 759.59) | 12.61 (14.69 to 10.72) |
| Nicaragua | 129.71 (100.01 to 181.55) | 3.34 (2.57 to 4.67) |  | 233.25 (153.45 to 310.25) | 3.5 (4.65 to 2.3) |
| Niger | 380.7 (206.9 to 619.07) | 4.74 (2.58 to 7.71) |  | 540.1 (318.07 to 894.76) | 2.16 (3.57 to 1.27) |
| Nigeria | 5373.32 (3514.8 to 9408.28) | 5.97 (3.9 to 10.45) |  | 9607.19 (6412.34 to 15132.9) | 4.16 (6.55 to 2.77) |
| Niue | 0.02 (0.01 to 0.03) | 0.88 (0.48 to 1.41) |  | 0.02 (0.01 to 0.04) | 1.37 (2.2 to 0.86) |
| North Macedonia | 89.72 (68.15 to 121.41) | 4.5 (3.42 to 6.09) |  | 104.74 (64.08 to 138.46) | 4.81 (6.36 to 2.94) |
| Northern Mariana Islands | 0.05 (0.02 to 0.09) | 0.11 (0.04 to 0.2) |  | 0.07 (0.03 to 0.11) | 0.14 (0.22 to 0.06) |
| Norway | 407.75 (380.88 to 433.98) | 9.6 (8.97 to 10.22) |  | 982.89 (886.13 to 1093.08) | 18.14 (20.17 to 16.36) |
| Oman | 35.37 (23.37 to 53.21) | 1.78 (1.18 to 2.68) |  | 62.79 (41.59 to 93.67) | 1.33 (1.99 to 0.88) |
| Pakistan | 6767.26 (4892.29 to 10127.34) | 6.09 (4.4 to 9.11) |  | 13703.54 (8915.5 to 23931.72) | 5.82 (10.16 to 3.78) |
| Palau | 0.03 (0.01 to 0.06) | 0.22 (0.08 to 0.4) |  | 0.05 (0.02 to 0.08) | 0.26 (0.42 to 0.12) |
| Palestine | 29.98 (17.74 to 44.12) | 1.46 (0.87 to 2.16) |  | 58.15 (31.89 to 82.41) | 1.13 (1.6 to 0.62) |
| Panama | 90.44 (81.48 to 100.24) | 3.79 (3.41 to 4.2) |  | 267.06 (216.53 to 320.89) | 6.22 (7.48 to 5.04) |
| Papua New Guinea | 28.65 (14.02 to 45.73) | 0.7 (0.34 to 1.11) |  | 64.93 (39.73 to 100.1) | 0.62 (0.96 to 0.38) |
| Paraguay | 129.67 (88.12 to 183.47) | 3.21 (2.18 to 4.54) |  | 327.96 (210.1 to 473.75) | 4.57 (6.61 to 2.93) |
| Peru | 1237.01 (749.26 to 1720.52) | 5.72 (3.46 to 7.95) |  | 1543.28 (1021.5 to 2259.85) | 4.25 (6.23 to 2.82) |
| Philippines | 2041.15 (1354.7 to 2442.34) | 3.24 (2.15 to 3.88) |  | 3510.4 (2309.05 to 4358.16) | 3.1 (3.85 to 2.04) |
| Poland | 2016.88 (1878.58 to 2156.43) | 5.28 (4.92 to 5.65) |  | 6007.77 (5368.92 to 6591.95) | 15.71 (17.24 to 14.04) |
| Portugal | 1038.79 (921 to 1173.33) | 10.25 (9.09 to 11.57) |  | 1948.14 (1681.52 to 2265.78) | 18.37 (21.36 to 15.85) |
| Puerto Rico | 310.79 (264.62 to 365.33) | 8.6 (7.33 to 10.11) |  | 346.74 (281.04 to 420.32) | 10.53 (12.76 to 8.53) |
| Qatar | 7.57 (4.2 to 11.06) | 1.7 (0.94 to 2.49) |  | 35.41 (18.79 to 54.73) | 1.19 (1.84 to 0.63) |
| Republic of Korea | 2065.73 (1517.59 to 3139.86) | 4.67 (3.43 to 7.1) |  | 3171.24 (1549.4 to 4371.38) | 6.15 (8.48 to 3) |
| Republic of Moldova | 155.59 (135.46 to 175.75) | 3.5 (3.05 to 3.95) |  | 218.99 (187.96 to 252.73) | 6.09 (7.03 to 5.23) |
| Romania | 1479.28 (1178.66 to 1857.81) | 6.33 (5.04 to 7.95) |  | 2154.14 (1699.94 to 2639.09) | 11.37 (13.94 to 8.98) |
| Russian Federation | 8542.25 (6469.36 to 9676.52) | 5.66 (4.28 to 6.41) |  | 14589.34 (13193.35 to 15913.98) | 10.07 (10.99 to 9.11) |
| Rwanda | 781.13 (508.08 to 1268.32) | 10.87 (7.07 to 17.64) |  | 925.64 (556.22 to 1522.8) | 6.98 (11.48 to 4.19) |
| Saint Kitts and Nevis | 1.71 (1.4 to 2.07) | 4.11 (3.38 to 4.99) |  | 2.7 (2.2 to 3.29) | 4.6 (5.61 to 3.75) |
| Saint Lucia | 8.42 (7.4 to 9.59) | 6.17 (5.42 to 7.02) |  | 14.23 (11.65 to 17.2) | 8.01 (9.69 to 6.56) |
| Saint Vincent and the Grenadines | 5.51 (4.91 to 6.25) | 5.04 (4.48 to 5.71) |  | 12.3 (10.51 to 14.32) | 10.78 (12.55 to 9.22) |
| Samoa | 5.09 (1.22 to 13.75) | 3.01 (0.72 to 8.14) |  | 7.25 (1.5 to 21.18) | 3.39 (9.91 to 0.7) |
| San Marino | 3.33 (2.32 to 5.03) | 14 (9.76 to 21.19) |  | 3.54 (2.04 to 5.89) | 10.82 (17.99 to 6.24) |
| Sao Tome and Principe | 3.74 (2.24 to 6.28) | 3.08 (1.84 to 5.17) |  | 3.97 (2.57 to 6.79) | 1.83 (3.13 to 1.19) |
| Saudi Arabia | 547.24 (351.42 to 798.41) | 3.45 (2.22 to 5.04) |  | 1468.55 (905.56 to 2414.98) | 3.89 (6.41 to 2.4) |
| Senegal | 234 (138.13 to 381.98) | 3.07 (1.81 to 5) |  | 315.81 (195.82 to 523.56) | 1.99 (3.3 to 1.23) |
| Serbia | 498.06 (337.15 to 681.98) | 5.17 (3.5 to 7.08) |  | 587.79 (355.37 to 821.77) | 6.59 (9.21 to 3.98) |
| Seychelles | 2.49 (1.79 to 3.71) | 3.42 (2.46 to 5.09) |  | 3.71 (2.67 to 5.54) | 3.52 (5.26 to 2.53) |
| Sierra Leone | 160.22 (95.9 to 257.83) | 3.86 (2.31 to 6.21) |  | 222.14 (139.86 to 351.44) | 2.51 (3.96 to 1.58) |
| Singapore | 238.02 (210.75 to 269.26) | 7.81 (6.92 to 8.84) |  | 616.44 (513.24 to 741.1) | 10.76 (12.94 to 8.96) |
| Slovakia | 387.14 (279.26 to 545.23) | 7.33 (5.29 to 10.32) |  | 407.45 (278.52 to 564.58) | 7.5 (10.4 to 5.13) |
| Slovenia | 154.2 (130.62 to 180.66) | 7.81 (6.62 to 9.15) |  | 278.93 (222.6 to 351.49) | 13.48 (16.98 to 10.76) |
| Solomon Islands | 1.97 (0.89 to 3.24) | 0.58 (0.26 to 0.96) |  | 4.83 (2.79 to 7.68) | 0.71 (1.12 to 0.41) |
| Somalia | 549.86 (310.59 to 974.76) | 6.93 (3.91 to 12.28) |  | 1296.33 (744.36 to 2266.22) | 6 (10.49 to 3.45) |
| South Africa | 1352.03 (890.93 to 1679.14) | 3.65 (2.41 to 4.54) |  | 2775.05 (1639.78 to 3440.17) | 4.88 (6.05 to 2.88) |
| South Sudan | 454.71 (265.17 to 820.52) | 7.74 (4.51 to 13.96) |  | 879.81 (524.76 to 1489.15) | 9.1 (15.4 to 5.43) |
| Spain | 4362.09 (4042.18 to 4694.79) | 11.25 (10.42 to 12.11) |  | 8265.58 (7059.91 to 9535.65) | 18.15 (20.93 to 15.5) |
| Sri Lanka | 511.18 (384.05 to 752.47) | 2.98 (2.24 to 4.39) |  | 714.46 (434.39 to 1074.49) | 3.21 (4.82 to 1.95) |
| Sudan | 1419.33 (889.16 to 2132.2) | 7.09 (4.44 to 10.65) |  | 1593.38 (981.1 to 2478.01) | 3.67 (5.71 to 2.26) |
| Suriname | 23.3 (16.59 to 34.01) | 6.03 (4.29 to 8.79) |  | 39.73 (27.14 to 57.23) | 6.86 (9.88 to 4.69) |
| Sweden | 1391.13 (1245.29 to 1547.74) | 16.2 (14.5 to 18.02) |  | 2220.41 (1846.65 to 2610.77) | 21.4 (25.17 to 17.8) |
| Switzerland | 897.62 (778.98 to 1040.48) | 13.07 (11.34 to 15.15) |  | 1605.53 (1329.12 to 1927.07) | 17.99 (21.6 to 14.9) |
| Syrian Arab Republic | 459.74 (309.8 to 631.18) | 3.62 (2.44 to 4.96) |  | 594.77 (361.18 to 853.31) | 4.24 (6.08 to 2.57) |
| Taiwan (Province of China) | 1215.59 (1122.31 to 1323.77) | 5.96 (5.5 to 6.49) |  | 1969.73 (1640.16 to 2305.33) | 8.33 (9.75 to 6.94) |
| Tajikistan | 112.56 (72.98 to 165.43) | 2.1 (1.36 to 3.08) |  | 167.84 (105.25 to 252.29) | 1.65 (2.48 to 1.04) |
| Thailand | 1758.12 (1161.69 to 2317.11) | 3.1 (2.05 to 4.08) |  | 3051.72 (2167.13 to 4518.19) | 4.58 (6.78 to 3.25) |
| Timor-Leste | 21.32 (13.62 to 31.88) | 2.73 (1.74 to 4.08) |  | 31.5 (21.4 to 47.39) | 2.25 (3.39 to 1.53) |
| Togo | 89.15 (51.86 to 140.62) | 2.44 (1.42 to 3.86) |  | 188.99 (116.1 to 293.99) | 2.26 (3.51 to 1.39) |
| Tokelau | 0.02 (0.01 to 0.02) | 0.97 (0.54 to 1.5) |  | 0.02 (0.01 to 0.03) | 1.3 (2.18 to 0.81) |
| Tonga | 0.52 (0.29 to 0.8) | 0.52 (0.3 to 0.81) |  | 0.61 (0.36 to 0.97) | 0.57 (0.91 to 0.33) |
| Trinidad and Tobago | 75.73 (67.71 to 86.65) | 6.29 (5.62 to 7.19) |  | 131.14 (98.91 to 167.83) | 9.41 (12.05 to 7.1) |
| Tunisia | 379.73 (265.33 to 591.77) | 4.55 (3.18 to 7.09) |  | 627.26 (418.62 to 986.67) | 5.3 (8.33 to 3.53) |
| Turkey | 3810.05 (2758.23 to 5401.44) | 6.63 (4.8 to 9.4) |  | 5217.06 (3682.17 to 7285.71) | 6.24 (8.71 to 4.4) |
| Turkmenistan | 81.49 (55.36 to 121.15) | 2.2 (1.5 to 3.28) |  | 182.76 (122.83 to 276.71) | 3.54 (5.36 to 2.38) |
| Tuvalu | 0.1 (0.05 to 0.15) | 1.06 (0.58 to 1.53) |  | 0.11 (0.07 to 0.16) | 0.91 (1.33 to 0.54) |
| Uganda | 1820.31 (1271.91 to 2598.69) | 10.53 (7.36 to 15.03) |  | 3650.66 (2333.93 to 5942.1) | 8.43 (13.72 to 5.39) |
| Ukraine | 3997.87 (3351.31 to 4783.77) | 7.58 (6.36 to 9.08) |  | 4023.35 (2926.89 to 5329.37) | 9.34 (12.37 to 6.79) |
| United Arab Emirates | 75.3 (51.36 to 110.84) | 4.03 (2.75 to 5.92) |  | 281.66 (194.48 to 387.51) | 2.92 (4.02 to 2.02) |
| United Kingdom | 7062.89 (6816.83 to 7307.41) | 12.33 (11.9 to 12.75) |  | 12678.73 (11883.58 to 13365.72) | 18.69 (19.7 to 17.51) |
| United Republic of Tanzania | 2184.8 (1362.51 to 3552.49) | 8.46 (5.27 to 13.75) |  | 3831.21 (2333.73 to 6322.86) | 6.55 (10.82 to 3.99) |
| United States of America | 39958.75 (38391.34 to 41407.66) | 15.73 (15.11 to 16.3) |  | 62942.42 (58808.58 to 65975.25) | 18.92 (19.83 to 17.68) |
| United States Virgin Islands | 7.27 (5.14 to 11.07) | 6.86 (4.84 to 10.44) |  | 5.29 (3.66 to 8.05) | 6.15 (9.38 to 4.26) |
| Uruguay | 286.71 (241.75 to 339.55) | 9.13 (7.7 to 10.82) |  | 433.66 (363.54 to 518.63) | 12.73 (15.23 to 10.67) |
| Uzbekistan | 136.75 (96.64 to 189.95) | 0.65 (0.46 to 0.91) |  | 854.11 (618.45 to 1147.75) | 2.5 (3.35 to 1.81) |
| Vanuatu | 0.79 (0.41 to 1.26) | 0.52 (0.27 to 0.83) |  | 1.92 (1.11 to 2.94) | 0.61 (0.94 to 0.35) |
| Venezuela (Bolivarian Republic of) | 652.5 (526.99 to 762.81) | 3.47 (2.8 to 4.06) |  | 1747.46 (1288.9 to 2277.35) | 6.56 (8.55 to 4.84) |
| Viet Nam | 2320.15 (1598.9 to 3490.56) | 3.4 (2.34 to 5.12) |  | 5068.68 (3316.72 to 8115.77) | 5.06 (8.09 to 3.31) |
| Yemen | 654.65 (396.16 to 941.62) | 4.8 (2.91 to 6.91) |  | 1054.83 (619.17 to 1522.89) | 3.14 (4.53 to 1.84) |
| Zambia | 704.77 (454.3 to 1167.97) | 8.88 (5.72 to 14.72) |  | 1337.78 (729.67 to 2556.62) | 6.86 (13.1 to 3.74) |
| Zimbabwe | 358.7 (249.27 to 532.04) | 3.47 (2.41 to 5.14) |  | 1024.62 (646.27 to 1621.79) | 6.57 (10.4 to 4.14) |

ASPR, age-standard prevalence rate. UI, uncertainty interval.
