# Supplementary material for: Global, regional, and national burden of soft tissue and other extraosseous sarcomas, 1990–2021: A Systematic analysis for the global burden of disease study 2021
Source: PLoS One. 2026 Mar 9;21(3):e0342986. doi: 10.1371/journal.pone.0342986 (PMC12970919; doi:10.1371/journal.pone.0342986)
Supplement: S3 Appendix — Age-standardized mortality rate (per 100,000 population) and death cases in 204 countries in 1990 and 2021. (DOCX) [file pone.0342986.s003.docx]

Appendix Table 3. Age-standardized mortality rate (per 100,000 population) and deaths cases in 204 countries in 1990 and 2021

| Location | Deaths in 1990 | |  | Deaths in 2021 | |
| --- | --- | --- | --- | --- | --- |
|  | Counts (95% UI) | ASMR (per 100,000) |  | Counts (95% UI) | ASMR (per 100,000) |
| Afghanistan | 137.84 (81.96 to 201.29) | 1.39 (0.82 to 2.02) |  | 191.27 (112.66 to 277.66) | 0.61 (0.36 to 0.89) |
| Albania | 12.2 (8.65 to 16.65) | 0.37 (0.26 to 0.5) |  | 12.18 (7.66 to 18.01) | 0.46 (0.29 to 0.67) |
| Algeria | 103.38 (71.86 to 139.43) | 0.41 (0.28 to 0.55) |  | 135.01 (95.48 to 199.56) | 0.31 (0.22 to 0.45) |
| American Samoa | 0.04 (0.02 to 0.08) | 0.07 (0.04 to 0.16) |  | 0.08 (0.05 to 0.15) | 0.16 (0.09 to 0.3) |
| Andorra | 0.4 (0.26 to 0.58) | 0.73 (0.47 to 1.08) |  | 0.63 (0.36 to 0.94) | 0.73 (0.42 to 1.1) |
| Angola | 75.81 (45.61 to 128.68) | 0.74 (0.44 to 1.25) |  | 142.62 (86.77 to 222.77) | 0.44 (0.27 to 0.68) |
| Antigua and Barbuda | 0.36 (0.32 to 0.39) | 0.6 (0.54 to 0.65) |  | 0.62 (0.57 to 0.67) | 0.69 (0.64 to 0.75) |
| Argentina | 255.91 (223.62 to 294.72) | 0.77 (0.68 to 0.89) |  | 353.92 (309.5 to 400.14) | 0.78 (0.68 to 0.88) |
| Armenia | 10.74 (7.45 to 14.9) | 0.31 (0.22 to 0.44) |  | 39.69 (28.44 to 54.66) | 1.32 (0.95 to 1.82) |
| Australia | 183.32 (170.89 to 195.9) | 1.09 (1.01 to 1.16) |  | 404.24 (349.19 to 459.17) | 1.57 (1.35 to 1.78) |
| Austria | 96.75 (89.09 to 104.28) | 1.25 (1.15 to 1.34) |  | 140.77 (120.65 to 161.47) | 1.57 (1.34 to 1.8) |
| Azerbaijan | 13.22 (7.97 to 20.53) | 0.18 (0.11 to 0.28) |  | 20.4 (11.38 to 33.73) | 0.19 (0.11 to 0.32) |
| Bahamas | 1.62 (1.43 to 1.8) | 0.63 (0.56 to 0.7) |  | 3.14 (2.5 to 3.9) | 0.81 (0.65 to 1.01) |
| Bahrain | 1.36 (0.93 to 1.86) | 0.27 (0.18 to 0.37) |  | 3.15 (1.95 to 4.4) | 0.21 (0.13 to 0.29) |
| Bangladesh | 580.54 (403.81 to 883.02) | 0.53 (0.37 to 0.81) |  | 646.03 (376.02 to 1173.17) | 0.39 (0.23 to 0.71) |
| Barbados | 3.47 (3.15 to 3.75) | 1.37 (1.24 to 1.48) |  | 5.53 (4.41 to 6.79) | 1.85 (1.47 to 2.27) |
| Belarus | 109.66 (95.39 to 125.33) | 1.05 (0.91 to 1.2) |  | 99.87 (78.55 to 124.5) | 1.07 (0.84 to 1.34) |
| Belgium | 102.45 (89.33 to 116.74) | 1.03 (0.9 to 1.17) |  | 195.96 (166.07 to 224.11) | 1.71 (1.45 to 1.95) |
| Belize | 0.45 (0.39 to 0.5) | 0.24 (0.21 to 0.27) |  | 1.04 (0.91 to 1.18) | 0.24 (0.21 to 0.27) |
| Benin | 16.54 (9.81 to 26.34) | 0.34 (0.2 to 0.54) |  | 32.12 (20.49 to 52.1) | 0.24 (0.15 to 0.39) |
| Bermuda | 0.9 (0.74 to 1.11) | 1.51 (1.24 to 1.86) |  | 1.34 (1.07 to 1.73) | 2.11 (1.68 to 2.72) |
| Bhutan | 3.08 (2.11 to 4.51) | 0.49 (0.33 to 0.72) |  | 3.2 (1.7 to 5.96) | 0.42 (0.23 to 0.79) |
| Bolivia (Plurinational State of) | 54.89 (39.02 to 72.92) | 0.86 (0.61 to 1.14) |  | 77.3 (51.66 to 109.26) | 0.66 (0.44 to 0.93) |
| Bosnia and Herzegovina | 16.14 (10.68 to 23.27) | 0.36 (0.24 to 0.52) |  | 17.66 (11.55 to 25.82) | 0.53 (0.35 to 0.78) |
| Botswana | 7.1 (4.59 to 10.17) | 0.54 (0.35 to 0.77) |  | 14.91 (9.79 to 22.3) | 0.62 (0.41 to 0.93) |
| Brazil | 652.22 (612.45 to 690.93) | 0.44 (0.41 to 0.47) |  | 1684.95 (1553.1 to 1794.58) | 0.76 (0.7 to 0.81) |
| Brunei Darussalam | 2.44 (1.67 to 3.75) | 0.94 (0.65 to 1.45) |  | 3.76 (2.72 to 5.34) | 0.83 (0.6 to 1.18) |
| Bulgaria | 32.72 (24.81 to 41.33) | 0.38 (0.29 to 0.48) |  | 56.79 (42.01 to 75.06) | 0.84 (0.62 to 1.11) |
| Burkina Faso | 40.65 (26.85 to 63.48) | 0.43 (0.28 to 0.67) |  | 69.28 (45.57 to 107.41) | 0.3 (0.2 to 0.47) |
| Burundi | 79.04 (52.83 to 122.54) | 1.42 (0.95 to 2.21) |  | 97.89 (65.87 to 155.33) | 0.74 (0.5 to 1.17) |
| Cabo Verde | 0.61 (0.4 to 1.04) | 0.17 (0.11 to 0.29) |  | 1.29 (0.86 to 1.99) | 0.23 (0.15 to 0.36) |
| Cambodia | 52.1 (37.96 to 71.76) | 0.51 (0.37 to 0.7) |  | 83.71 (56.44 to 122.58) | 0.49 (0.33 to 0.72) |
| Cameroon | 34.78 (23.14 to 54.23) | 0.33 (0.22 to 0.52) |  | 95.33 (58.44 to 146.1) | 0.3 (0.18 to 0.46) |
| Canada | 305.2 (278.74 to 330.84) | 1.12 (1.02 to 1.21) |  | 568.61 (495.37 to 660.26) | 1.52 (1.32 to 1.76) |
| Central African Republic | 21.12 (14.14 to 33.9) | 0.77 (0.52 to 1.24) |  | 39.94 (25.23 to 63.42) | 0.73 (0.46 to 1.16) |
| Chad | 21.12 (13.57 to 33.89) | 0.35 (0.23 to 0.56) |  | 57.13 (37.62 to 91.71) | 0.32 (0.21 to 0.52) |
| Chile | 81.98 (70.08 to 95.57) | 0.62 (0.53 to 0.72) |  | 169.29 (139.75 to 201.43) | 0.9 (0.74 to 1.07) |
| China | 3779.85 (2624.85 to 4875.04) | 0.32 (0.22 to 0.41) |  | 4634.82 (3208.18 to 6509.71) | 0.33 (0.23 to 0.46) |
| Colombia | 153.78 (137.83 to 168.73) | 0.47 (0.42 to 0.52) |  | 413.84 (337.59 to 496.06) | 0.84 (0.69 to 1.01) |
| Comoros | 5.43 (3.41 to 8.83) | 1.17 (0.74 to 1.91) |  | 7.87 (4.81 to 13.18) | 1.06 (0.65 to 1.77) |
| Congo | 14.97 (10.03 to 23.8) | 0.62 (0.42 to 0.99) |  | 27.42 (17.86 to 42.62) | 0.51 (0.33 to 0.79) |
| Cook Islands | 0.01 (0.01 to 0.02) | 0.08 (0.04 to 0.12) |  | 0.02 (0.01 to 0.02) | 0.09 (0.05 to 0.14) |
| Costa Rica | 22.87 (19.46 to 26.38) | 0.75 (0.64 to 0.87) |  | 51.83 (42.8 to 61.37) | 1.09 (0.9 to 1.29) |
| Croatia | 38.18 (30.87 to 46.06) | 0.79 (0.63 to 0.95) |  | 55.4 (43.88 to 68.68) | 1.32 (1.04 to 1.63) |
| Cuba | 98.84 (84.81 to 117.36) | 0.91 (0.78 to 1.08) |  | 108.97 (89.37 to 131.96) | 0.97 (0.79 to 1.17) |
| Cyprus | 8.62 (6.38 to 11.66) | 1.11 (0.82 to 1.5) |  | 11.11 (7.04 to 14.81) | 0.82 (0.52 to 1.09) |
| Czechia | 111.49 (95.93 to 130.27) | 1.08 (0.93 to 1.27) |  | 151.81 (115.65 to 195.02) | 1.43 (1.09 to 1.83) |
| Saint Helena | 28.9 (19.47 to 40.89) | 0.24 (0.16 to 0.34) |  | 65.13 (35.5 to 98.57) | 0.23 (0.13 to 0.35) |
| Democratic People's Republic of Korea | 86.91 (57.38 to 134.39) | 0.42 (0.28 to 0.65) |  | 148.5 (99.29 to 230.04) | 0.56 (0.38 to 0.87) |
| Democratic Republic of the Congo | 215.37 (136.86 to 337.58) | 0.56 (0.36 to 0.88) |  | 370.71 (234.89 to 592.94) | 0.41 (0.26 to 0.66) |
| Denmark | 68.71 (60.61 to 77.68) | 1.34 (1.18 to 1.51) |  | 83.38 (70.03 to 96.49) | 1.42 (1.2 to 1.65) |
| Djibouti | 2.7 (1.62 to 4.58) | 0.65 (0.39 to 1.11) |  | 11.3 (6.77 to 19.94) | 0.9 (0.54 to 1.58) |
| Dominica | 0.47 (0.34 to 0.69) | 0.65 (0.47 to 0.95) |  | 0.55 (0.38 to 0.76) | 0.82 (0.57 to 1.13) |
| Dominican Republic | 20.15 (13.84 to 27.04) | 0.28 (0.19 to 0.38) |  | 37.09 (19.67 to 54.86) | 0.34 (0.18 to 0.5) |
| Ecuador | 34.11 (30.15 to 38.53) | 0.34 (0.3 to 0.39) |  | 132.35 (104.8 to 166.17) | 0.73 (0.58 to 0.92) |
| Egypt | 141.91 (91.35 to 188.03) | 0.26 (0.17 to 0.34) |  | 140.83 (94.11 to 226.55) | 0.13 (0.09 to 0.21) |
| El Salvador | 21.79 (15.38 to 28.54) | 0.41 (0.29 to 0.54) |  | 32.18 (19.17 to 41.89) | 0.5 (0.3 to 0.65) |
| Equatorial Guinea | 2.96 (1.91 to 4.85) | 0.7 (0.45 to 1.15) |  | 4.99 (2.88 to 8.4) | 0.33 (0.19 to 0.56) |
| Eritrea | 37.9 (24.83 to 62.33) | 1.11 (0.73 to 1.83) |  | 69.13 (41.68 to 112.81) | 1.05 (0.63 to 1.71) |
| Estonia | 9.77 (7.91 to 12.18) | 0.62 (0.5 to 0.78) |  | 14.78 (11.23 to 18.63) | 1.13 (0.86 to 1.42) |
| Eswatini | 4.46 (3.2 to 6.4) | 0.55 (0.4 to 0.79) |  | 10.23 (5.88 to 14.96) | 0.89 (0.51 to 1.3) |
| Ethiopia | 1111.5 (793.21 to 1689.36) | 2.2 (1.57 to 3.34) |  | 1021.73 (712.18 to 1609.74) | 0.94 (0.65 to 1.48) |
| Fiji | 1.72 (0.88 to 3.28) | 0.23 (0.12 to 0.43) |  | 2.3 (1.06 to 4.93) | 0.25 (0.11 to 0.53) |
| Finland | 68.13 (60.24 to 76.92) | 1.36 (1.2 to 1.54) |  | 100.46 (84.69 to 117.06) | 1.81 (1.53 to 2.11) |
| France | 652.68 (599.64 to 701.57) | 1.13 (1.04 to 1.21) |  | 1141.04 (945.51 to 1363.33) | 1.72 (1.42 to 2.05) |
| Gabon | 6.19 (4.13 to 9.65) | 0.63 (0.42 to 0.98) |  | 9.37 (5.64 to 15.64) | 0.52 (0.31 to 0.86) |
| Gambia | 3.01 (1.96 to 4.55) | 0.31 (0.2 to 0.46) |  | 7.33 (4.56 to 10.96) | 0.31 (0.19 to 0.46) |
| Georgia | 1.52 (1.1 to 2.14) | 0.03 (0.02 to 0.04) |  | 56.27 (40.38 to 75.33) | 1.56 (1.12 to 2.09) |
| Germany | 951.05 (832.87 to 1057.74) | 1.19 (1.04 to 1.32) |  | 1768.14 (1540.55 to 1953.28) | 2.07 (1.8 to 2.29) |
| Ghana | 37.58 (24.86 to 57.24) | 0.25 (0.17 to 0.38) |  | 88.97 (58.74 to 134.86) | 0.26 (0.17 to 0.39) |
| Greece | 70.72 (66.63 to 75.11) | 0.68 (0.64 to 0.72) |  | 165.8 (149.48 to 180.24) | 1.63 (1.47 to 1.77) |
| Greenland | 0.6 (0.34 to 0.78) | 1.08 (0.61 to 1.4) |  | 0.39 (0.23 to 0.54) | 0.7 (0.41 to 0.96) |
| Grenada | 0.7 (0.56 to 0.84) | 0.8 (0.65 to 0.97) |  | 1.06 (0.88 to 1.29) | 1.03 (0.86 to 1.25) |
| Guam | 0.17 (0.11 to 0.23) | 0.13 (0.08 to 0.17) |  | 0.18 (0.13 to 0.3) | 0.12 (0.08 to 0.19) |
| Guatemala | 19.32 (16.21 to 26.04) | 0.23 (0.19 to 0.31) |  | 45.06 (38.27 to 52.35) | 0.29 (0.24 to 0.33) |
| Guinea | 37.47 (23.47 to 61.57) | 0.63 (0.39 to 1.03) |  | 54.8 (35.07 to 87.08) | 0.41 (0.26 to 0.65) |
| Guinea-Bissau | 5.12 (3.4 to 8.07) | 0.51 (0.34 to 0.8) |  | 7.1 (4.72 to 10.89) | 0.34 (0.23 to 0.53) |
| Guyana | 0.19 (0.16 to 0.24) | 0.02 (0.02 to 0.03) |  | 3.9 (2.81 to 5.22) | 0.51 (0.37 to 0.68) |
| Haiti | 92.1 (59.13 to 129.63) | 1.44 (0.93 to 2.03) |  | 132.59 (83.43 to 191.17) | 1.03 (0.65 to 1.49) |
| Honduras | 24.36 (17.54 to 33.55) | 0.52 (0.37 to 0.71) |  | 66.66 (43.59 to 95.29) | 0.66 (0.43 to 0.94) |
| Hungary | 126.42 (111.07 to 141.23) | 1.22 (1.07 to 1.36) |  | 167.88 (132.8 to 213.3) | 1.75 (1.38 to 2.22) |
| Iceland | 2.24 (2.02 to 2.44) | 0.88 (0.8 to 0.96) |  | 4.52 (3.91 to 5.23) | 1.29 (1.12 to 1.49) |
| India | 4465.64 (2526.34 to 5584.82) | 0.52 (0.3 to 0.65) |  | 6479.16 (4411.27 to 8404.16) | 0.46 (0.31 to 0.59) |
| Indonesia | 583.78 (427.13 to 840.59) | 0.32 (0.23 to 0.45) |  | 992.98 (732.17 to 1515.31) | 0.36 (0.26 to 0.54) |
| Iran (Islamic Republic of) | 316.09 (248.14 to 465.94) | 0.55 (0.43 to 0.82) |  | 368.29 (286.28 to 528.63) | 0.43 (0.34 to 0.62) |
| Iraq | 59.03 (40.94 to 81.6) | 0.32 (0.22 to 0.44) |  | 95.99 (61.66 to 136.37) | 0.23 (0.15 to 0.33) |
| Ireland | 38.25 (34.65 to 42.43) | 1.06 (0.96 to 1.18) |  | 57.36 (47.98 to 66.71) | 1.16 (0.97 to 1.35) |
| Israel | 53.1 (45.53 to 61.54) | 1.07 (0.92 to 1.24) |  | 119.92 (101.06 to 139.69) | 1.25 (1.05 to 1.46) |
| Italy | 441.39 (413.85 to 465.45) | 0.78 (0.73 to 0.82) |  | 997.52 (866.91 to 1099.8) | 1.67 (1.45 to 1.84) |
| Jamaica | 14.73 (12.39 to 17.38) | 0.62 (0.52 to 0.73) |  | 25.79 (19.21 to 33.69) | 0.92 (0.69 to 1.2) |
| Japan | 667.51 (634.25 to 687.73) | 0.53 (0.5 to 0.55) |  | 1400.98 (1210.74 to 1516.28) | 1.1 (0.95 to 1.19) |
| Jordan | 10.31 (6.87 to 14.15) | 0.28 (0.18 to 0.38) |  | 20.31 (13.74 to 30.39) | 0.16 (0.11 to 0.25) |
| Kazakhstan | 117.2 (89.94 to 152.39) | 0.71 (0.55 to 0.93) |  | 148.02 (107.08 to 199.84) | 0.78 (0.56 to 1.05) |
| Kenya | 121.81 (85.02 to 157.59) | 0.53 (0.37 to 0.68) |  | 297.76 (217.23 to 404.78) | 0.59 (0.43 to 0.81) |
| Kiribati | 0.18 (0.12 to 0.26) | 0.24 (0.16 to 0.34) |  | 0.24 (0.15 to 0.37) | 0.19 (0.12 to 0.31) |
| Kuwait | 5.54 (4.69 to 6.55) | 0.32 (0.27 to 0.38) |  | 6.95 (5.6 to 8.7) | 0.15 (0.12 to 0.19) |
| Kyrgyzstan | 22.99 (18.02 to 29.97) | 0.52 (0.4 to 0.67) |  | 31.95 (23.98 to 42.31) | 0.47 (0.35 to 0.62) |
| Lao People's Democratic Republic | 22.1 (14.24 to 32.38) | 0.53 (0.34 to 0.78) |  | 28.91 (19.17 to 44.62) | 0.39 (0.26 to 0.6) |
| Latvia | 29.56 (23.26 to 37.82) | 1.11 (0.88 to 1.42) |  | 29.55 (22.61 to 38.54) | 1.58 (1.21 to 2.06) |
| Lebanon | 23.9 (16.63 to 35.22) | 0.8 (0.56 to 1.18) |  | 28.97 (20.1 to 44.87) | 0.52 (0.36 to 0.81) |
| Lesotho | 8.08 (5.55 to 11.62) | 0.53 (0.36 to 0.76) |  | 19.79 (13.11 to 28.56) | 1.06 (0.7 to 1.52) |
| Liberia | 10.74 (7.02 to 17.49) | 0.44 (0.29 to 0.71) |  | 13.41 (8.56 to 20.59) | 0.25 (0.16 to 0.38) |
| Libya | 33.8 (21.19 to 56.41) | 0.8 (0.5 to 1.34) |  | 76.08 (47.58 to 124.15) | 1.11 (0.69 to 1.81) |
| Lithuania | 26.71 (21.05 to 33.77) | 0.73 (0.57 to 0.92) |  | 44.51 (34.27 to 56.02) | 1.63 (1.26 to 2.05) |
| Luxembourg | 4.45 (4.19 to 4.71) | 1.17 (1.1 to 1.24) |  | 8.46 (7.4 to 9.48) | 1.31 (1.15 to 1.47) |
| Madagascar | 102.15 (67.57 to 166.32) | 0.86 (0.57 to 1.4) |  | 193.3 (126.14 to 314.36) | 0.68 (0.44 to 1.1) |
| Malawi | 119.57 (81.48 to 165.19) | 1.22 (0.83 to 1.68) |  | 152.35 (100.15 to 215.72) | 0.78 (0.51 to 1.11) |
| Malaysia | 68.93 (50.58 to 100.14) | 0.39 (0.29 to 0.57) |  | 135.27 (100.25 to 199.38) | 0.43 (0.32 to 0.63) |
| Maldives | 0.51 (0.31 to 0.7) | 0.23 (0.14 to 0.32) |  | 0.45 (0.3 to 0.65) | 0.09 (0.06 to 0.13) |
| Mali | 26.14 (14.81 to 40.71) | 0.3 (0.17 to 0.47) |  | 39.09 (19.15 to 63.27) | 0.16 (0.08 to 0.26) |
| Malta | 3.78 (3.39 to 4.24) | 1.02 (0.92 to 1.14) |  | 8.27 (6.76 to 9.94) | 1.87 (1.53 to 2.25) |
| Marshall Islands | 0.04 (0.02 to 0.05) | 0.08 (0.05 to 0.12) |  | 0.07 (0.04 to 0.1) | 0.12 (0.07 to 0.18) |
| Mauritania | 6.33 (4.31 to 10.14) | 0.31 (0.21 to 0.49) |  | 9.39 (6.11 to 14.65) | 0.21 (0.14 to 0.33) |
| Mauritius | 2.1 (1.94 to 2.26) | 0.19 (0.18 to 0.21) |  | 8.72 (7.86 to 9.4) | 0.69 (0.62 to 0.74) |
| Mexico | 382.82 (371.96 to 395.74) | 0.45 (0.44 to 0.46) |  | 1021.08 (903.05 to 1132.48) | 0.79 (0.7 to 0.88) |
| Micronesia (Federated States of) | 0.1 (0.06 to 0.15) | 0.1 (0.05 to 0.15) |  | 0.12 (0.07 to 0.18) | 0.12 (0.07 to 0.18) |
| Monaco | 0.07 (0.02 to 0.12) | 0.23 (0.07 to 0.41) |  | 0.09 (0.03 to 0.15) | 0.24 (0.08 to 0.4) |
| Mongolia | 6.08 (3.39 to 10.99) | 0.28 (0.16 to 0.51) |  | 11.74 (7.75 to 16.46) | 0.35 (0.23 to 0.49) |
| Montenegro | 1.18 (0.62 to 1.59) | 0.19 (0.1 to 0.25) |  | 1.6 (0.71 to 2.31) | 0.26 (0.11 to 0.37) |
| Morocco | 76.5 (49.28 to 104.25) | 0.3 (0.19 to 0.41) |  | 113.39 (64.78 to 158.54) | 0.3 (0.17 to 0.43) |
| Mozambique | 161.98 (109.47 to 253.83) | 1.21 (0.82 to 1.9) |  | 286 (174.01 to 457.38) | 0.92 (0.56 to 1.47) |
| Myanmar | 235.82 (158.92 to 355.29) | 0.58 (0.39 to 0.88) |  | 237.6 (165.24 to 356.16) | 0.42 (0.29 to 0.63) |
| Namibia | 7.96 (5.86 to 11.4) | 0.57 (0.42 to 0.81) |  | 16.44 (10.69 to 26.26) | 0.68 (0.44 to 1.08) |
| Nauru | 0.01 (0.01 to 0.02) | 0.13 (0.07 to 0.19) |  | 0.01 (0.01 to 0.02) | 0.14 (0.08 to 0.19) |
| Nepal | 94.08 (62.93 to 144.52) | 0.48 (0.32 to 0.74) |  | 129.76 (74.52 to 237.86) | 0.42 (0.24 to 0.76) |
| Netherlands | 156.34 (140.57 to 173.09) | 1.05 (0.94 to 1.16) |  | 222.76 (186.87 to 259.56) | 1.29 (1.09 to 1.51) |
| New Zealand | 32.32 (27.86 to 37.06) | 0.95 (0.82 to 1.08) |  | 55.03 (47.11 to 63.61) | 1.06 (0.91 to 1.23) |
| Nicaragua | 12.88 (10.13 to 17.66) | 0.33 (0.26 to 0.45) |  | 24.2 (16 to 32.4) | 0.36 (0.24 to 0.49) |
| Niger | 41.69 (24.48 to 66.25) | 0.52 (0.3 to 0.82) |  | 61.33 (37.71 to 96.98) | 0.24 (0.15 to 0.39) |
| Nigeria | 638.95 (429.12 to 1102.44) | 0.71 (0.48 to 1.22) |  | 996.6 (682.59 to 1536.07) | 0.43 (0.3 to 0.66) |
| Niue | 0 (0 to 0) | 0.13 (0.07 to 0.21) |  | 0 (0 to 0) | 0.15 (0.1 to 0.23) |
| North Macedonia | 9.89 (7.64 to 13.46) | 0.5 (0.38 to 0.68) |  | 11.61 (7.29 to 15.2) | 0.53 (0.34 to 0.7) |
| Northern Mariana Islands | 0 (0 to 0.01) | 0.01 (0 to 0.02) |  | 0.01 (0 to 0.01) | 0.01 (0.01 to 0.02) |
| Norway | 43.13 (40.17 to 45.82) | 1.02 (0.95 to 1.08) |  | 82.12 (73.6 to 91.42) | 1.52 (1.36 to 1.69) |
| Oman | 3.6 (2.35 to 5.22) | 0.18 (0.12 to 0.26) |  | 5.03 (3.37 to 7.34) | 0.11 (0.07 to 0.16) |
| Pakistan | 929.37 (694.03 to 1406.98) | 0.84 (0.62 to 1.27) |  | 1674.2 (1132.4 to 2901.62) | 0.71 (0.48 to 1.23) |
| Palau | 0 (0 to 0.01) | 0.03 (0.01 to 0.05) |  | 0.01 (0 to 0.01) | 0.03 (0.01 to 0.05) |
| Palestine | 3.14 (1.89 to 4.41) | 0.15 (0.09 to 0.22) |  | 5.11 (2.88 to 7.06) | 0.1 (0.06 to 0.14) |
| Panama | 9.85 (9.08 to 10.68) | 0.41 (0.38 to 0.45) |  | 26.72 (21.48 to 32.33) | 0.62 (0.5 to 0.75) |
| Papua New Guinea | 3.68 (1.85 to 5.79) | 0.09 (0.05 to 0.14) |  | 7.91 (4.75 to 12.66) | 0.08 (0.05 to 0.12) |
| Paraguay | 14.55 (10.32 to 19.88) | 0.36 (0.26 to 0.49) |  | 36.01 (23.47 to 50.71) | 0.5 (0.33 to 0.71) |
| Peru | 146.74 (88.73 to 198.58) | 0.68 (0.41 to 0.92) |  | 161.97 (108.91 to 234.43) | 0.45 (0.3 to 0.65) |
| Philippines | 216.5 (142.13 to 261) | 0.34 (0.23 to 0.41) |  | 416.02 (275.92 to 520.23) | 0.37 (0.24 to 0.46) |
| Poland | 238.64 (224.93 to 251.49) | 0.63 (0.59 to 0.66) |  | 618.44 (558.73 to 674.24) | 1.62 (1.46 to 1.76) |
| Portugal | 106.81 (94.39 to 119.53) | 1.05 (0.93 to 1.18) |  | 179.29 (152.58 to 206.54) | 1.69 (1.44 to 1.95) |
| Puerto Rico | 32.85 (28.2 to 37.76) | 0.91 (0.78 to 1.05) |  | 35.04 (28.04 to 42.03) | 1.06 (0.85 to 1.28) |
| Qatar | 0.68 (0.39 to 0.96) | 0.15 (0.09 to 0.22) |  | 2.3 (1.25 to 3.53) | 0.08 (0.04 to 0.12) |
| Republic of Korea | 195.81 (148.54 to 288.96) | 0.44 (0.34 to 0.65) |  | 258 (127.81 to 361.15) | 0.5 (0.25 to 0.7) |
| Republic of Moldova | 18.12 (16.09 to 20.31) | 0.41 (0.36 to 0.46) |  | 24.64 (21.35 to 28.47) | 0.69 (0.59 to 0.79) |
| Romania | 174.55 (138.29 to 216.85) | 0.75 (0.59 to 0.93) |  | 232.24 (182.88 to 285.92) | 1.23 (0.97 to 1.51) |
| Russian Federation | 953.7 (743.33 to 1068.95) | 0.63 (0.49 to 0.71) |  | 1503.7 (1354.44 to 1645.81) | 1.04 (0.94 to 1.14) |
| Rwanda | 110.6 (74.81 to 171.3) | 1.54 (1.04 to 2.38) |  | 118.5 (73.88 to 197.91) | 0.89 (0.56 to 1.49) |
| Saint Kitts and Nevis | 0.24 (0.21 to 0.28) | 0.59 (0.51 to 0.68) |  | 0.31 (0.26 to 0.36) | 0.53 (0.44 to 0.62) |
| Saint Lucia | 0.99 (0.89 to 1.1) | 0.72 (0.65 to 0.8) |  | 1.59 (1.3 to 1.9) | 0.9 (0.73 to 1.07) |
| Saint Vincent and the Grenadines | 0.62 (0.56 to 0.68) | 0.57 (0.51 to 0.62) |  | 1.42 (1.23 to 1.63) | 1.25 (1.08 to 1.42) |
| Samoa | 0.68 (0.17 to 1.88) | 0.4 (0.1 to 1.11) |  | 0.85 (0.19 to 2.46) | 0.4 (0.09 to 1.15) |
| San Marino | 0.29 (0.2 to 0.46) | 1.24 (0.86 to 1.93) |  | 0.31 (0.19 to 0.5) | 0.95 (0.57 to 1.54) |
| Sao Tome and Principe | 0.42 (0.27 to 0.69) | 0.35 (0.23 to 0.57) |  | 0.48 (0.33 to 0.73) | 0.22 (0.15 to 0.33) |
| Saudi Arabia | 60.55 (39.01 to 85.06) | 0.38 (0.25 to 0.54) |  | 119.19 (77.28 to 193.35) | 0.32 (0.2 to 0.51) |
| Senegal | 26.63 (16.9 to 42.1) | 0.35 (0.22 to 0.55) |  | 39.77 (26.02 to 62.54) | 0.25 (0.16 to 0.39) |
| Serbia | 58.74 (40.07 to 80.84) | 0.61 (0.42 to 0.84) |  | 65.82 (40.76 to 91.24) | 0.74 (0.46 to 1.02) |
| Seychelles | 0.34 (0.25 to 0.49) | 0.47 (0.35 to 0.68) |  | 0.44 (0.32 to 0.65) | 0.42 (0.31 to 0.62) |
| Sierra Leone | 18.29 (11.56 to 28.73) | 0.44 (0.28 to 0.69) |  | 25.57 (16.62 to 39.03) | 0.29 (0.19 to 0.44) |
| Singapore | 21.66 (19.19 to 24.67) | 0.71 (0.63 to 0.81) |  | 47.36 (39.56 to 57.06) | 0.83 (0.69 to 1) |
| Slovakia | 44.2 (31.95 to 61.11) | 0.84 (0.6 to 1.16) |  | 41.02 (28.67 to 57.46) | 0.76 (0.53 to 1.06) |
| Slovenia | 15.93 (13.31 to 18.7) | 0.81 (0.67 to 0.95) |  | 26.27 (21.02 to 33.06) | 1.27 (1.02 to 1.6) |
| Solomon Islands | 0.29 (0.13 to 0.46) | 0.08 (0.04 to 0.14) |  | 0.64 (0.37 to 1.01) | 0.09 (0.05 to 0.15) |
| Somalia | 76.03 (45.5 to 127.96) | 0.96 (0.57 to 1.61) |  | 181.89 (109.58 to 309.58) | 0.84 (0.51 to 1.43) |
| South Africa | 148.99 (96.95 to 186.5) | 0.4 (0.26 to 0.5) |  | 328.07 (195.29 to 401.08) | 0.58 (0.34 to 0.71) |
| South Sudan | 60.06 (36.79 to 104.11) | 1.02 (0.63 to 1.77) |  | 103.35 (62.34 to 174.12) | 1.07 (0.64 to 1.8) |
| Spain | 395.9 (365.69 to 427.48) | 1.02 (0.94 to 1.1) |  | 682.09 (581.29 to 788.44) | 1.5 (1.28 to 1.73) |
| Sri Lanka | 60.25 (44.84 to 86.37) | 0.35 (0.26 to 0.5) |  | 76.47 (47.56 to 113.55) | 0.34 (0.21 to 0.51) |
| Sudan | 161.93 (99.66 to 235.18) | 0.81 (0.5 to 1.17) |  | 158.02 (96.98 to 240.01) | 0.36 (0.22 to 0.55) |
| Suriname | 2.75 (2.02 to 4.02) | 0.71 (0.52 to 1.04) |  | 4.65 (3.12 to 6.86) | 0.8 (0.54 to 1.18) |
| Sweden | 132.79 (118.5 to 147.61) | 1.55 (1.38 to 1.72) |  | 193.5 (161.82 to 226.97) | 1.87 (1.56 to 2.19) |
| Switzerland | 87.23 (75.95 to 100.34) | 1.27 (1.11 to 1.46) |  | 133.67 (108.98 to 159.88) | 1.5 (1.22 to 1.79) |
| Syrian Arab Republic | 48.07 (31.46 to 64.26) | 0.38 (0.25 to 0.51) |  | 59.53 (34.72 to 86.92) | 0.42 (0.25 to 0.62) |
| Taiwan (Province of China) | 114.13 (105.26 to 122.7) | 0.56 (0.52 to 0.6) |  | 179.76 (151.49 to 210.41) | 0.76 (0.64 to 0.89) |
| Tajikistan | 12.29 (8.29 to 18.08) | 0.23 (0.15 to 0.34) |  | 18.58 (11.88 to 27.72) | 0.18 (0.12 to 0.27) |
| Thailand | 195.8 (127.83 to 256.98) | 0.34 (0.23 to 0.45) |  | 310.36 (219 to 459.87) | 0.47 (0.33 to 0.69) |
| Timor-Leste | 2.52 (1.67 to 3.66) | 0.32 (0.21 to 0.47) |  | 4.05 (2.76 to 6.17) | 0.29 (0.2 to 0.44) |
| Togo | 9.96 (6.46 to 15.56) | 0.27 (0.18 to 0.43) |  | 22.95 (14.64 to 34.77) | 0.27 (0.17 to 0.42) |
| Tokelau | 0 (0 to 0) | 0.14 (0.08 to 0.22) |  | 0 (0 to 0) | 0.14 (0.09 to 0.21) |
| Tonga | 0.07 (0.04 to 0.1) | 0.07 (0.04 to 0.1) |  | 0.07 (0.04 to 0.11) | 0.07 (0.04 to 0.11) |
| Trinidad and Tobago | 8.91 (8.15 to 9.77) | 0.74 (0.68 to 0.81) |  | 14.48 (10.99 to 18.16) | 1.04 (0.79 to 1.3) |
| Tunisia | 39.6 (28.48 to 58.96) | 0.47 (0.34 to 0.71) |  | 63.99 (42.03 to 97.87) | 0.54 (0.35 to 0.83) |
| Turkey | 437.77 (320.11 to 608.58) | 0.76 (0.56 to 1.06) |  | 494.9 (360.23 to 705.81) | 0.59 (0.43 to 0.84) |
| Turkmenistan | 9.64 (6.9 to 13.31) | 0.26 (0.19 to 0.36) |  | 20.45 (14.09 to 30.65) | 0.4 (0.27 to 0.59) |
| Tuvalu | 0.02 (0.01 to 0.02) | 0.17 (0.09 to 0.23) |  | 0.02 (0.01 to 0.02) | 0.12 (0.07 to 0.18) |
| Uganda | 228.7 (166.67 to 324.63) | 1.32 (0.96 to 1.88) |  | 420.78 (278.78 to 695.96) | 0.97 (0.64 to 1.61) |
| Ukraine | 444.62 (373.4 to 532.3) | 0.84 (0.71 to 1.01) |  | 427.43 (311.72 to 566.3) | 0.99 (0.72 to 1.31) |
| United Arab Emirates | 7.61 (5.32 to 10.83) | 0.41 (0.28 to 0.58) |  | 24.23 (16.95 to 33.61) | 0.25 (0.18 to 0.35) |
| United Kingdom | 695.64 (669.26 to 712.98) | 1.21 (1.17 to 1.24) |  | 1133.62 (1051.22 to 1187.47) | 1.67 (1.55 to 1.75) |
| United Republic of Tanzania | 268.13 (177.81 to 436.2) | 1.04 (0.69 to 1.69) |  | 454.43 (284.79 to 759.42) | 0.78 (0.49 to 1.3) |
| United States of America | 3600.49 (3432.25 to 3699.73) | 1.42 (1.35 to 1.46) |  | 5626.65 (5198.22 to 5862.52) | 1.69 (1.56 to 1.76) |
| United States Virgin Islands | 0.77 (0.56 to 1.16) | 0.73 (0.53 to 1.09) |  | 0.62 (0.43 to 0.92) | 0.72 (0.5 to 1.08) |
| Uruguay | 35.29 (29.91 to 42.12) | 1.12 (0.95 to 1.34) |  | 47.96 (39.65 to 57.9) | 1.41 (1.16 to 1.7) |
| Uzbekistan | 16.28 (11.28 to 22.64) | 0.08 (0.05 to 0.11) |  | 94.75 (69.08 to 123.74) | 0.28 (0.2 to 0.36) |
| Vanuatu | 0.11 (0.06 to 0.17) | 0.07 (0.04 to 0.11) |  | 0.26 (0.15 to 0.39) | 0.08 (0.05 to 0.13) |
| Venezuela (Bolivarian Republic of) | 74.87 (61.57 to 84.56) | 0.4 (0.33 to 0.45) |  | 193.56 (144.68 to 247.27) | 0.73 (0.54 to 0.93) |
| Viet Nam | 300.82 (214.72 to 446.12) | 0.44 (0.31 to 0.65) |  | 573.81 (384.31 to 906.54) | 0.57 (0.38 to 0.9) |
| Yemen | 78.8 (46 to 112.92) | 0.58 (0.34 to 0.83) |  | 120.3 (68.57 to 172.11) | 0.36 (0.2 to 0.51) |
| Zambia | 88.69 (59.95 to 141.16) | 1.12 (0.76 to 1.78) |  | 160.47 (94.07 to 294.57) | 0.82 (0.48 to 1.51) |
| Zimbabwe | 46.75 (33.01 to 70.26) | 0.45 (0.32 to 0.68) |  | 134.16 (87.01 to 210.72) | 0.86 (0.56 to 1.35) |

ASMR, age-standard mortality rate. UI, uncertainty interval.
