# Supplementary material for: Global, regional, and national burden of soft tissue and other extraosseous sarcomas, 1990–2021: A Systematic analysis for the global burden of disease study 2021
Source: PLoS One. 2026 Mar 9;21(3):e0342986. doi: 10.1371/journal.pone.0342986 (PMC12970919; doi:10.1371/journal.pone.0342986)
Supplement: S4 Appendix — Age-standardized DALYs rate (per 100,000 person-years) and DALYs counts in 204 countries in 1990 and 2021. (DOCX) [file pone.0342986.s004.docx]

Appendix Table 4. Age-standardized DALYs rate (per 100,000 population) and DALYs counts in 204 countries in 1990 and 2021

| Location | DALYs in 1990 | |  | DALYs in 2021 | |
| --- | --- | --- | --- | --- | --- |
|  | Counts (95% UI) | ASDR (per 100,000) |  | Counts (95% UI) | ASDR (per 100,000) |
| Afghanistan | 6130.08 (3587.48 to 8910.04) | 61.65 (36.08 to 89.61) |  | 9700.1 (5669.36 to 14211.34) | 31.07 (18.16 to 45.52) |
| Albania | 602.45 (417.46 to 809.29) | 18.23 (12.63 to 24.49) |  | 330.65 (204.18 to 488.85) | 12.39 (7.65 to 18.32) |
| Algeria | 5292.77 (3900.59 to 7380.65) | 20.93 (15.42 to 29.19) |  | 4934.44 (3344.92 to 7743.5) | 11.16 (7.57 to 17.52) |
| American Samoa | 1.47 (0.68 to 3.14) | 3.04 (1.41 to 6.47) |  | 2.75 (1.54 to 5.18) | 5.53 (3.1 to 10.42) |
| Andorra | 13.96 (8.81 to 20.52) | 25.68 (16.2 to 37.75) |  | 17.14 (9.62 to 26.07) | 20.03 (11.24 to 30.46) |
| Angola | 4872.09 (2659.76 to 8491.43) | 47.41 (25.88 to 82.63) |  | 7420.64 (4390.32 to 11850.57) | 22.69 (13.42 to 36.23) |
| Antigua and Barbuda | 13.27 (12.01 to 14.68) | 22.03 (19.94 to 24.38) |  | 20.86 (19.19 to 22.86) | 23.33 (21.46 to 25.57) |
| Argentina | 9306.74 (8010.09 to 10803.62) | 28.11 (24.19 to 32.63) |  | 11573.59 (9959.2 to 13294.77) | 25.44 (21.89 to 29.23) |
| Armenia | 399.05 (263.46 to 569.12) | 11.67 (7.7 to 16.64) |  | 1111.02 (782.85 to 1556.16) | 37.09 (26.14 to 51.95) |
| Australia | 5687.78 (5292.9 to 6091.1) | 33.74 (31.4 to 36.13) |  | 10860.69 (9441.85 to 12431.36) | 42.11 (36.61 to 48.2) |
| Austria | 2898.71 (2688.14 to 3119.06) | 37.32 (34.6 to 40.15) |  | 3598.43 (3133.44 to 4095.36) | 40.06 (34.88 to 45.59) |
| Azerbaijan | 589.51 (345.56 to 901.16) | 8.05 (4.72 to 12.3) |  | 725.55 (404.46 to 1190.01) | 6.91 (3.85 to 11.33) |
| Bahamas | 75.77 (67.04 to 84.81) | 29.52 (26.12 to 33.04) |  | 124.01 (98.5 to 156.99) | 31.96 (25.39 to 40.47) |
| Bahrain | 61.88 (41.79 to 85.54) | 12.22 (8.25 to 16.89) |  | 131.67 (78.4 to 185.67) | 8.61 (5.13 to 12.14) |
| Bangladesh | 32131.55 (21016.68 to 49832.29) | 29.45 (19.26 to 45.67) |  | 23388.79 (13365.08 to 42962.18) | 14.21 (8.12 to 26.1) |
| Barbados | 122.01 (109.89 to 133.77) | 48.15 (43.36 to 52.79) |  | 162.98 (127.82 to 204.48) | 54.5 (42.75 to 68.38) |
| Belarus | 3379.7 (2930.83 to 3878.27) | 32.36 (28.06 to 37.13) |  | 2793.56 (2181.31 to 3477.25) | 29.96 (23.39 to 37.29) |
| Belgium | 3202.99 (2759.21 to 3684.59) | 32.1 (27.65 to 36.92) |  | 5133.63 (4405.04 to 5842.68) | 44.76 (38.41 to 50.94) |
| Belize | 24.52 (22.45 to 27.05) | 13.12 (12.01 to 14.47) |  | 46.14 (40.53 to 52.27) | 10.75 (9.45 to 12.18) |
| Benin | 1057.43 (558.55 to 1722.2) | 21.8 (11.52 to 35.5) |  | 1801.94 (1070.68 to 3183.48) | 13.35 (7.93 to 23.58) |
| Bermuda | 30.58 (25.11 to 37.88) | 51.49 (42.28 to 63.8) |  | 34.28 (27.09 to 44.11) | 53.95 (42.64 to 69.42) |
| Bhutan | 167.51 (109.1 to 243.2) | 26.59 (17.32 to 38.6) |  | 108.12 (54.56 to 209.25) | 14.28 (7.21 to 27.65) |
| Bolivia (Plurinational State of) | 2685.22 (1842.92 to 3746.22) | 42.08 (28.88 to 58.71) |  | 2713.74 (1788.68 to 3862.91) | 23 (15.16 to 32.74) |
| Bosnia and Herzegovina | 603.34 (392.37 to 884.99) | 13.41 (8.72 to 19.67) |  | 457.81 (296.73 to 673.96) | 13.86 (8.98 to 20.41) |
| Botswana | 332.72 (212.83 to 478.18) | 25.22 (16.13 to 36.25) |  | 640.98 (414.99 to 957.36) | 26.78 (17.34 to 40) |
| Brazil | 29124.74 (27383.45 to 31076.85) | 19.61 (18.44 to 20.93) |  | 59635.66 (55532.53 to 63486.75) | 27.06 (25.2 to 28.81) |
| Brunei Darussalam | 121.98 (81.39 to 191.09) | 47.05 (31.39 to 73.71) |  | 152.59 (108.69 to 221.19) | 33.82 (24.09 to 49.03) |
| Bulgaria | 1140.12 (849.26 to 1458.7) | 13.13 (9.78 to 16.81) |  | 1525.46 (1116.53 to 2047.86) | 22.48 (16.45 to 30.17) |
| Burkina Faso | 2514.88 (1487.59 to 4049.96) | 26.39 (15.61 to 42.51) |  | 3831.69 (2384.02 to 6255.26) | 16.83 (10.47 to 27.48) |
| Burundi | 4309.38 (2761.88 to 6678.19) | 77.6 (49.73 to 120.25) |  | 4734.44 (3096.59 to 7439.11) | 35.81 (23.42 to 56.27) |
| Cabo Verde | 26.94 (16.15 to 48.76) | 7.62 (4.56 to 13.78) |  | 42.23 (26.34 to 70.43) | 7.55 (4.71 to 12.59) |
| Cambodia | 2730.78 (1913.85 to 3871.55) | 26.58 (18.63 to 37.69) |  | 3194.65 (2158.58 to 4856.01) | 18.74 (12.66 to 28.49) |
| Cameroon | 2024.82 (1235.13 to 3236.56) | 19.4 (11.83 to 31.01) |  | 4890.31 (2974.35 to 7909.11) | 15.39 (9.36 to 24.89) |
| Canada | 9653.49 (8883.89 to 10436.49) | 35.42 (32.6 to 38.29) |  | 15724.24 (13750.81 to 18237.85) | 41.97 (36.7 to 48.67) |
| Central African Republic | 1174.98 (754.43 to 1955.22) | 43.03 (27.63 to 71.6) |  | 2079.16 (1331.86 to 3323.45) | 37.91 (24.29 to 60.6) |
| Chad | 1308.82 (756.45 to 2192.07) | 21.72 (12.55 to 36.37) |  | 3428.05 (2112.69 to 5642.83) | 19.31 (11.9 to 31.79) |
| Chile | 3218.07 (2773.62 to 3749.76) | 24.22 (20.88 to 28.22) |  | 5296.47 (4388.46 to 6268.26) | 28.18 (23.35 to 33.35) |
| China | 150779.15 (106970.1 to 196128.63) | 12.82 (9.09 to 16.67) |  | 123444.93 (85594.41 to 175887.1) | 8.68 (6.02 to 12.36) |
| Colombia | 7289.4 (6514.45 to 8083.23) | 22.43 (20.05 to 24.88) |  | 14244 (11629.68 to 17170.54) | 29.03 (23.7 to 35) |
| Comoros | 283.59 (174.23 to 457) | 61.31 (37.66 to 98.8) |  | 320.03 (190.64 to 536.4) | 42.99 (25.61 to 72.06) |
| Congo | 752.18 (495.47 to 1224.77) | 31.32 (20.63 to 50.99) |  | 1220.45 (790.97 to 1872.85) | 22.64 (14.67 to 34.74) |
| Cook Islands | 0.56 (0.31 to 0.93) | 2.97 (1.65 to 4.9) |  | 0.5 (0.28 to 0.8) | 2.83 (1.57 to 4.49) |
| Costa Rica | 864.24 (728.6 to 1028.59) | 28.41 (23.95 to 33.82) |  | 1717.91 (1411.16 to 2061.77) | 36.18 (29.72 to 43.42) |
| Croatia | 1222.28 (986.42 to 1471.55) | 25.14 (20.29 to 30.27) |  | 1329.22 (1037.64 to 1647.6) | 31.58 (24.65 to 39.15) |
| Cuba | 3217.15 (2717.67 to 3896.92) | 29.66 (25.05 to 35.93) |  | 3184.63 (2590.86 to 3918.52) | 28.26 (22.99 to 34.77) |
| Cyprus | 246.12 (179.99 to 332.41) | 31.63 (23.13 to 42.72) |  | 289.91 (176.47 to 393.02) | 21.35 (13 to 28.95) |
| Czechia | 3352.06 (2874.2 to 3919.59) | 32.56 (27.92 to 38.07) |  | 3851.01 (2942.65 to 4952.22) | 36.22 (27.68 to 46.58) |
| Saint Helena | 1757.98 (1140.93 to 2556.4) | 14.41 (9.35 to 20.96) |  | 3311.13 (1798.8 to 5508.33) | 11.88 (6.46 to 19.77) |
| Democratic People's Republic of Korea | 3345.53 (2124.41 to 5222.06) | 16.25 (10.32 to 25.36) |  | 4721.39 (3071.14 to 7493.95) | 17.89 (11.64 to 28.39) |
| Democratic Republic of the Congo | 12675.94 (7697.35 to 20199.91) | 33.23 (20.18 to 52.95) |  | 17721.02 (11244.55 to 27643.34) | 19.69 (12.49 to 30.71) |
| Denmark | 1810.7 (1587.6 to 2060.81) | 35.2 (30.86 to 40.06) |  | 2105.79 (1774.13 to 2437.13) | 35.99 (30.32 to 41.65) |
| Djibouti | 148.51 (87.25 to 252.64) | 35.85 (21.06 to 60.99) |  | 482.26 (284.37 to 886.41) | 38.31 (22.59 to 70.42) |
| Dominica | 17.32 (12.35 to 24.93) | 23.92 (17.05 to 34.43) |  | 19.68 (13.42 to 27.44) | 29.34 (20.01 to 40.91) |
| Dominican Republic | 1222.59 (838.64 to 1668.43) | 17.1 (11.73 to 23.33) |  | 1559.08 (841.49 to 2399.64) | 14.16 (7.64 to 21.79) |
| Ecuador | 1516.86 (1291.96 to 1781.47) | 15.2 (12.95 to 17.85) |  | 4521.15 (3545.9 to 5758.06) | 25.03 (19.63 to 31.87) |
| Egypt | 6986.28 (4620.72 to 9486.96) | 12.63 (8.35 to 17.15) |  | 5477.34 (3521.77 to 9085.78) | 5.19 (3.33 to 8.6) |
| El Salvador | 1071.78 (753.2 to 1391.06) | 20.2 (14.2 to 26.22) |  | 1182.12 (693.82 to 1557.57) | 18.33 (10.76 to 24.15) |
| Equatorial Guinea | 161.3 (100.67 to 264.47) | 38.15 (23.81 to 62.55) |  | 246.56 (135 to 424.21) | 16.3 (8.93 to 28.05) |
| Eritrea | 2109.2 (1322.57 to 3549.15) | 61.93 (38.83 to 104.2) |  | 3269.83 (1933.57 to 5536.8) | 49.56 (29.31 to 83.92) |
| Estonia | 279.74 (222.21 to 358.99) | 17.83 (14.17 to 22.89) |  | 344.62 (259.94 to 443.9) | 26.29 (19.83 to 33.86) |
| Eswatini | 224.27 (158.45 to 328.13) | 27.81 (19.65 to 40.69) |  | 460.44 (260.91 to 681.41) | 39.86 (22.59 to 58.98) |
| Ethiopia | 61605.02 (41882.44 to 94242.06) | 121.82 (82.82 to 186.36) |  | 48053.68 (32709.19 to 77218.29) | 44.11 (30.03 to 70.88) |
| Fiji | 74.28 (37.69 to 142.27) | 9.79 (4.97 to 18.76) |  | 87.54 (39.66 to 187.5) | 9.47 (4.29 to 20.28) |
| Finland | 1881.77 (1648.4 to 2134.88) | 37.56 (32.9 to 42.61) |  | 2500.76 (2115.45 to 2913.44) | 45.17 (38.21 to 52.63) |
| France | 20293.34 (18483.01 to 21733.56) | 35.13 (31.99 to 37.62) |  | 28858.32 (24389.52 to 34318.41) | 43.47 (36.74 to 51.69) |
| Gabon | 271.66 (178.92 to 432.15) | 27.62 (18.19 to 43.94) |  | 390.71 (226.71 to 658.87) | 21.51 (12.48 to 36.28) |
| Gambia | 186.45 (113.25 to 283.41) | 18.99 (11.54 to 28.87) |  | 340.52 (212.64 to 534.16) | 14.22 (8.88 to 22.31) |
| Georgia | 53.82 (38.67 to 76.11) | 0.97 (0.7 to 1.38) |  | 1571.58 (1125.15 to 2107.41) | 43.57 (31.19 to 58.42) |
| Germany | 28064.1 (24644.67 to 31444.34) | 35.11 (30.83 to 39.33) |  | 44263.48 (39021.38 to 48991.82) | 51.85 (45.71 to 57.39) |
| Ghana | 2129.64 (1308.33 to 3302.14) | 14.22 (8.74 to 22.06) |  | 3962.76 (2543.15 to 6291.33) | 11.57 (7.43 to 18.37) |
| Greece | 2101.66 (1978.99 to 2239.11) | 20.23 (19.05 to 21.55) |  | 4055.4 (3737.36 to 4373.93) | 39.86 (36.73 to 42.99) |
| Greenland | 25.79 (15.07 to 34.04) | 46.42 (27.12 to 61.27) |  | 13.37 (8.14 to 17.94) | 23.83 (14.51 to 31.97) |
| Grenada | 28.56 (22.34 to 35.29) | 32.83 (25.68 to 40.56) |  | 38.27 (31.74 to 47.61) | 37.29 (30.93 to 46.39) |
| Guam | 7.1 (4.61 to 9.78) | 5.19 (3.37 to 7.15) |  | 6.93 (5.01 to 11.66) | 4.36 (3.15 to 7.32) |
| Guatemala | 1031.44 (838.9 to 1484.36) | 12.3 (10.01 to 17.7) |  | 1874.85 (1588.72 to 2186.92) | 11.89 (10.08 to 13.87) |
| Guinea | 2410.16 (1392.21 to 3966.72) | 40.21 (23.23 to 66.18) |  | 2857.89 (1740.57 to 4832.25) | 21.28 (12.96 to 35.98) |
| Guinea-Bissau | 312.22 (189.46 to 505.64) | 31 (18.81 to 50.21) |  | 354.86 (224.51 to 558.15) | 17.19 (10.88 to 27.04) |
| Guyana | 10.09 (8.17 to 12.3) | 1.29 (1.05 to 1.58) |  | 171.28 (121.69 to 233.01) | 22.4 (15.91 to 30.47) |
| Haiti | 5926.74 (3549.29 to 8836.14) | 92.88 (55.62 to 138.48) |  | 7428.73 (4396.71 to 10929.5) | 57.75 (34.18 to 84.96) |
| Honduras | 1282.22 (895 to 1828.23) | 27.22 (19 to 38.81) |  | 2359.53 (1526.63 to 3469.11) | 23.34 (15.1 to 34.31) |
| Hungary | 4145.14 (3659.57 to 4662.03) | 39.88 (35.21 to 44.85) |  | 4643.48 (3691.13 to 5882.95) | 48.38 (38.46 to 61.3) |
| Iceland | 67.55 (60.78 to 74.12) | 26.61 (23.94 to 29.19) |  | 128.59 (109.82 to 149.72) | 36.7 (31.34 to 42.73) |
| India | 211305.23 (120310.26 to 265167.49) | 24.77 (14.1 to 31.08) |  | 221602.82 (151663.38 to 292094.84) | 15.67 (10.72 to 20.65) |
| Indonesia | 28674.09 (20610.35 to 41193.24) | 15.5 (11.14 to 22.27) |  | 36831.83 (27030.47 to 57228.78) | 13.21 (9.69 to 20.52) |
| Iran (Islamic Republic of) | 16803.2 (12834.93 to 25449.23) | 29.43 (22.48 to 44.57) |  | 13010.48 (10004.96 to 20101.88) | 15.24 (11.72 to 23.55) |
| Iraq | 3049.29 (2125.06 to 4384.19) | 16.56 (11.54 to 23.8) |  | 4008.61 (2642.09 to 5645.77) | 9.72 (6.41 to 13.7) |
| Ireland | 1234.3 (1118.83 to 1370.5) | 34.27 (31.06 to 38.05) |  | 1608.8 (1358.25 to 1874.49) | 32.56 (27.49 to 37.93) |
| Israel | 1859.29 (1583.14 to 2175.9) | 37.48 (31.91 to 43.86) |  | 3442.57 (2906.88 to 4036.2) | 35.88 (30.3 to 42.07) |
| Italy | 14450.71 (13701.54 to 15165.71) | 25.44 (24.12 to 26.7) |  | 24567.28 (22029.97 to 26875.53) | 41.07 (36.83 to 44.93) |
| Jamaica | 628.32 (527.3 to 770.26) | 26.56 (22.29 to 32.56) |  | 879.15 (643.44 to 1162.96) | 31.4 (22.98 to 41.54) |
| Japan | 23689.2 (23001.58 to 24338.21) | 18.83 (18.28 to 19.34) |  | 31445.84 (28846.53 to 33285.64) | 24.63 (22.59 to 26.07) |
| Jordan | 517.7 (354.21 to 725.5) | 13.86 (9.48 to 19.42) |  | 865.65 (585.08 to 1321.74) | 7.02 (4.75 to 10.72) |
| Kazakhstan | 4575.25 (3537.59 to 5998.37) | 27.91 (21.58 to 36.59) |  | 5216.41 (3866.23 to 7002.8) | 27.52 (20.4 to 36.94) |
| Kenya | 6392.35 (4444.31 to 8416.59) | 27.62 (19.2 to 36.36) |  | 12347.28 (8898.85 to 16985.14) | 24.66 (17.78 to 33.93) |
| Kiribati | 8.71 (5.64 to 13.1) | 11.7 (7.58 to 17.61) |  | 10.09 (6.17 to 15.6) | 8.33 (5.09 to 12.87) |
| Kuwait | 252.25 (210.88 to 302.18) | 14.68 (12.27 to 17.58) |  | 315.06 (249.1 to 397.1) | 6.78 (5.36 to 8.54) |
| Kyrgyzstan | 974.5 (731.98 to 1319.71) | 21.83 (16.4 to 29.56) |  | 1275.87 (929.44 to 1715.42) | 18.59 (13.54 to 24.99) |
| Lao People's Democratic Republic | 1151.56 (700 to 1752.89) | 27.62 (16.79 to 42.04) |  | 1212.84 (790.28 to 1897.86) | 16.44 (10.71 to 25.72) |
| Latvia | 873.96 (671.26 to 1141.98) | 32.88 (25.25 to 42.96) |  | 730.74 (555.75 to 970.33) | 39.07 (29.71 to 51.88) |
| Lebanon | 947.28 (649.09 to 1426.57) | 31.66 (21.69 to 47.68) |  | 921.07 (628.5 to 1421.88) | 16.62 (11.34 to 25.66) |
| Lesotho | 337.71 (231.6 to 484.05) | 22.04 (15.11 to 31.58) |  | 834.19 (547.48 to 1232.52) | 44.5 (29.21 to 65.76) |
| Liberia | 703.57 (420.03 to 1149.87) | 28.59 (17.07 to 46.73) |  | 669.7 (414.69 to 1063.79) | 12.27 (7.6 to 19.49) |
| Libya | 1573.81 (932.75 to 2744.52) | 37.34 (22.13 to 65.11) |  | 3027.58 (1777.94 to 5200.87) | 44.07 (25.88 to 75.7) |
| Lithuania | 798.06 (627.41 to 1017.64) | 21.72 (17.08 to 27.7) |  | 1112.77 (850.61 to 1400.6) | 40.79 (31.18 to 51.34) |
| Luxembourg | 145.17 (136.68 to 154.62) | 38.08 (35.86 to 40.56) |  | 223.06 (197.05 to 250.3) | 34.62 (30.58 to 38.85) |
| Madagascar | 5659.15 (3555.97 to 9394.54) | 47.55 (29.88 to 78.94) |  | 9664.71 (6115.59 to 15731.1) | 33.84 (21.42 to 55.09) |
| Malawi | 7481.12 (4966 to 10899.11) | 76.29 (50.64 to 111.15) |  | 7356.54 (4557.04 to 10937.08) | 37.83 (23.43 to 56.24) |
| Malaysia | 2769.27 (1993.09 to 4071.08) | 15.67 (11.28 to 23.04) |  | 4541.68 (3368.74 to 6678.6) | 14.28 (10.59 to 20.99) |
| Maldives | 26.83 (16.25 to 37.69) | 12.07 (7.31 to 16.96) |  | 17.34 (11.23 to 24.68) | 3.35 (2.17 to 4.77) |
| Mali | 1717.93 (953.94 to 2901.48) | 19.83 (11.01 to 33.49) |  | 2173.73 (1023.7 to 3801.49) | 9.02 (4.25 to 15.77) |
| Malta | 124.28 (111.05 to 138.69) | 33.54 (29.97 to 37.42) |  | 219.56 (179.61 to 268.32) | 49.65 (40.62 to 60.68) |
| Marshall Islands | 1.64 (0.94 to 2.41) | 3.62 (2.07 to 5.31) |  | 2.82 (1.68 to 4.28) | 5.01 (2.99 to 7.61) |
| Mauritania | 322.37 (197.03 to 538.16) | 15.69 (9.59 to 26.19) |  | 396.14 (245.52 to 656.46) | 9.01 (5.58 to 14.93) |
| Mauritius | 83.3 (77.38 to 89.69) | 7.6 (7.06 to 8.18) |  | 282.78 (255.15 to 304.94) | 22.23 (20.06 to 23.97) |
| Mexico | 18773.31 (18086.85 to 19570.87) | 21.99 (21.18 to 22.92) |  | 37835.5 (33615.01 to 41897.94) | 29.27 (26 to 32.41) |
| Micronesia (Federated States of) | 4.37 (2.44 to 6.66) | 4.22 (2.36 to 6.44) |  | 4.75 (2.76 to 7.17) | 4.63 (2.69 to 6.99) |
| Monaco | 1.82 (0.56 to 3.23) | 5.99 (1.85 to 10.63) |  | 2.25 (0.71 to 3.78) | 5.95 (1.89 to 9.97) |
| Mongolia | 308.05 (169.45 to 563.35) | 14.28 (7.85 to 26.11) |  | 467.97 (309.01 to 651.8) | 14.03 (9.26 to 19.54) |
| Montenegro | 45.6 (23.29 to 61.73) | 7.28 (3.72 to 9.86) |  | 43.59 (18.96 to 63.88) | 7.05 (3.07 to 10.34) |
| Morocco | 3603.92 (2446.85 to 5173.35) | 14.21 (9.65 to 20.4) |  | 3797.71 (2336.31 to 5562.2) | 10.22 (6.28 to 14.96) |
| Mozambique | 9718.78 (6121.05 to 15265.69) | 72.75 (45.82 to 114.26) |  | 14135.88 (8328.52 to 23126.05) | 45.49 (26.8 to 74.43) |
| Myanmar | 12197.83 (7935.4 to 18516.47) | 30.16 (19.62 to 45.79) |  | 9195.57 (6209.12 to 13986.24) | 16.3 (11.01 to 24.79) |
| Namibia | 360.3 (261.59 to 520.45) | 25.66 (18.63 to 37.07) |  | 675.1 (433.5 to 1112.27) | 27.77 (17.83 to 45.75) |
| Nauru | 0.62 (0.33 to 0.95) | 6.03 (3.23 to 9.32) |  | 0.64 (0.37 to 0.93) | 5.81 (3.38 to 8.45) |
| Nepal | 5027.63 (3162.21 to 7861.95) | 25.82 (16.24 to 40.38) |  | 4604.15 (2554.13 to 8544.16) | 14.79 (8.2 to 27.45) |
| Netherlands | 4683.36 (4238.8 to 5182.06) | 31.39 (28.41 to 34.73) |  | 5308.37 (4428.88 to 6162.15) | 30.84 (25.73 to 35.8) |
| New Zealand | 1101.16 (957.2 to 1256.49) | 32.22 (28.01 to 36.77) |  | 1557.49 (1334.31 to 1801.49) | 30.13 (25.81 to 34.85) |
| Nicaragua | 742.89 (575.89 to 1011.37) | 19.11 (14.81 to 26.02) |  | 937.85 (614.33 to 1245.98) | 14.06 (9.21 to 18.68) |
| Niger | 3059.69 (1675.7 to 4866.41) | 38.09 (20.86 to 60.59) |  | 3594.97 (2135.3 to 5816.93) | 14.36 (8.53 to 23.23) |
| Nigeria | 39729.12 (26186.17 to 68978.09) | 44.12 (29.08 to 76.61) |  | 58325.3 (38331.36 to 91854.94) | 25.23 (16.58 to 39.74) |
| Niue | 0.1 (0.05 to 0.16) | 4.21 (2.28 to 6.88) |  | 0.1 (0.06 to 0.16) | 5.86 (3.68 to 9.46) |
| North Macedonia | 394.81 (300.6 to 525.11) | 19.82 (15.09 to 26.36) |  | 322.83 (196.81 to 430.59) | 14.83 (9.04 to 19.78) |
| Northern Mariana Islands | 0.22 (0.07 to 0.4) | 0.5 (0.16 to 0.88) |  | 0.26 (0.11 to 0.41) | 0.54 (0.23 to 0.84) |
| Norway | 1150.77 (1084.59 to 1215.33) | 27.1 (25.54 to 28.62) |  | 2123.69 (1927.81 to 2362.83) | 39.2 (35.58 to 43.61) |
| Oman | 174.33 (115.39 to 262.31) | 8.79 (5.81 to 13.22) |  | 213.99 (141.76 to 319.82) | 4.55 (3.01 to 6.8) |
| Pakistan | 44780.68 (32336.19 to 67752.71) | 40.3 (29.1 to 60.97) |  | 76773.8 (50939.82 to 131672.58) | 32.59 (21.63 to 55.9) |
| Palau | 0.16 (0.06 to 0.3) | 1.07 (0.38 to 1.96) |  | 0.18 (0.08 to 0.3) | 1.01 (0.47 to 1.63) |
| Palestine | 155.5 (92.05 to 230.55) | 7.6 (4.5 to 11.26) |  | 225.42 (123.11 to 320.47) | 4.39 (2.4 to 6.24) |
| Panama | 422.92 (389.41 to 459.85) | 17.7 (16.3 to 19.25) |  | 936.01 (762.82 to 1137.36) | 21.81 (17.77 to 26.5) |
| Papua New Guinea | 182.06 (86.68 to 293.01) | 4.44 (2.11 to 7.14) |  | 378.55 (231.21 to 582.4) | 3.62 (2.21 to 5.57) |
| Paraguay | 666.7 (457.02 to 950) | 16.49 (11.3 to 23.5) |  | 1316.88 (839.36 to 1905.54) | 18.37 (11.71 to 26.58) |
| Peru | 7132.72 (4332.66 to 9844.81) | 32.97 (20.03 to 45.51) |  | 5404.22 (3552.8 to 7947.65) | 14.9 (9.8 to 21.91) |
| Philippines | 11586.64 (7689.13 to 14297.52) | 18.39 (12.2 to 22.69) |  | 16746.5 (11144.8 to 20648.3) | 14.79 (9.84 to 18.23) |
| Poland | 8295.58 (7842.21 to 8733.29) | 21.73 (20.54 to 22.88) |  | 16882.95 (15358.76 to 18387.9) | 44.15 (40.17 to 48.09) |
| Portugal | 3647.18 (3257.41 to 4098.62) | 35.98 (32.13 to 40.43) |  | 4464.74 (3908.85 to 5235.24) | 42.09 (36.85 to 49.35) |
| Puerto Rico | 1216.23 (1041.27 to 1413.03) | 33.67 (28.83 to 39.12) |  | 958.9 (769.61 to 1165.85) | 29.11 (23.36 to 35.39) |
| Qatar | 33.88 (18.89 to 49.02) | 7.62 (4.25 to 11.02) |  | 108.57 (57.6 to 170.95) | 3.65 (1.93 to 5.74) |
| Republic of Korea | 8871 (6545.49 to 13358.73) | 20.05 (14.79 to 30.19) |  | 7285.47 (3570.61 to 9930.83) | 14.13 (6.92 to 19.26) |
| Republic of Moldova | 682.29 (598.06 to 766.7) | 15.34 (13.45 to 17.24) |  | 721.39 (621.45 to 833.8) | 20.08 (17.29 to 23.2) |
| Romania | 6480.32 (5164.02 to 8163.77) | 27.72 (22.09 to 34.92) |  | 6410.61 (5101.64 to 7878.74) | 33.85 (26.94 to 41.6) |
| Russian Federation | 34086.34 (25899.33 to 38502.96) | 22.58 (17.15 to 25.5) |  | 44469.67 (40466.05 to 48612.95) | 30.7 (27.94 to 33.56) |
| Rwanda | 6118.93 (3994.66 to 9754.56) | 85.11 (55.56 to 135.68) |  | 5264.19 (3201.46 to 8889.13) | 39.67 (24.13 to 66.99) |
| Saint Kitts and Nevis | 9.54 (8.03 to 11.35) | 23 (19.36 to 27.37) |  | 10.74 (8.76 to 13) | 18.32 (14.94 to 22.18) |
| Saint Lucia | 44.28 (39.72 to 49.17) | 32.42 (29.08 to 36) |  | 55.86 (45.75 to 67.04) | 31.47 (25.77 to 37.76) |
| Saint Vincent and the Grenadines | 28.9 (25.91 to 32.04) | 26.39 (23.66 to 29.26) |  | 52.97 (45.4 to 61.35) | 46.44 (39.8 to 53.78) |
| Samoa | 27.61 (6.49 to 76.29) | 16.34 (3.84 to 45.16) |  | 33.1 (6.85 to 97.77) | 15.49 (3.21 to 45.76) |
| San Marino | 8.58 (6.03 to 12.93) | 36.12 (25.37 to 54.43) |  | 7.59 (4.37 to 12.47) | 23.17 (13.35 to 38.07) |
| Sao Tome and Principe | 24.76 (14.61 to 42.32) | 20.41 (12.05 to 34.89) |  | 18.93 (12.14 to 32.56) | 8.74 (5.61 to 15.03) |
| Saudi Arabia | 2961.27 (1954.01 to 4301.78) | 18.68 (12.32 to 27.13) |  | 5208.65 (3190.44 to 8757.23) | 13.81 (8.46 to 23.23) |
| Senegal | 1667.48 (966.87 to 2767.75) | 21.85 (12.67 to 36.26) |  | 1746 (1103.71 to 2996.15) | 11.01 (6.96 to 18.89) |
| Serbia | 2003.64 (1365.1 to 2745.31) | 20.81 (14.18 to 28.51) |  | 1641.68 (1010.14 to 2264.56) | 18.41 (11.33 to 25.39) |
| Seychelles | 11.89 (8.78 to 17.63) | 16.32 (12.06 to 24.2) |  | 14.06 (10.35 to 21.04) | 13.34 (9.81 to 19.96) |
| Sierra Leone | 1190.65 (702.7 to 1921.02) | 28.68 (16.93 to 46.27) |  | 1361.79 (862.57 to 2182.62) | 15.36 (9.73 to 24.61) |
| Singapore | 870.14 (772.11 to 987.51) | 28.56 (25.34 to 32.41) |  | 1470.5 (1235.93 to 1764) | 25.68 (21.58 to 30.8) |
| Slovakia | 1469.41 (1055.39 to 2083.35) | 27.81 (19.98 to 39.43) |  | 1124.11 (768.55 to 1573.64) | 20.7 (14.16 to 28.98) |
| Slovenia | 498.91 (421.99 to 580.2) | 25.28 (21.38 to 29.4) |  | 603 (488.09 to 764.54) | 29.14 (23.58 to 36.94) |
| Solomon Islands | 13.08 (6.11 to 21.56) | 3.86 (1.8 to 6.36) |  | 27.62 (15.85 to 43.42) | 4.04 (2.32 to 6.35) |
| Somalia | 4330.85 (2403.05 to 7457.06) | 54.55 (30.27 to 93.93) |  | 9265.39 (5388.67 to 16161.01) | 42.88 (24.94 to 74.8) |
| South Africa | 7561.23 (4954.77 to 9342.04) | 20.43 (13.39 to 25.24) |  | 13109.63 (7724.89 to 16085.56) | 23.06 (13.59 to 28.29) |
| South Sudan | 3294.88 (1900.07 to 5703.61) | 56.06 (32.33 to 97.04) |  | 5518.99 (3367.6 to 9265.85) | 57.06 (34.82 to 95.8) |
| Spain | 13165.75 (12315.92 to 14098.1) | 33.95 (31.76 to 36.35) |  | 17889.15 (15444.15 to 20467.78) | 39.27 (33.91 to 44.94) |
| Sri Lanka | 2563.06 (1896.1 to 3689.53) | 14.96 (11.07 to 21.54) |  | 2399.81 (1455.89 to 3627.92) | 10.78 (6.54 to 16.29) |
| Sudan | 9548.69 (6062.96 to 14294.06) | 47.69 (30.28 to 71.39) |  | 7803.18 (4766.04 to 12205.53) | 17.97 (10.98 to 28.11) |
| Suriname | 133.29 (95.38 to 195.59) | 34.47 (24.66 to 50.58) |  | 186.2 (126.07 to 270.01) | 32.14 (21.76 to 46.61) |
| Sweden | 3741.32 (3336.75 to 4185.29) | 43.57 (38.85 to 48.74) |  | 4583.18 (3817.22 to 5413.69) | 44.18 (36.8 to 52.19) |
| Switzerland | 2340.25 (2027.15 to 2705.21) | 34.08 (29.52 to 39.4) |  | 3326.59 (2772.33 to 3958.07) | 37.28 (31.07 to 44.36) |
| Syrian Arab Republic | 2462.2 (1692.5 to 3352.41) | 19.36 (13.31 to 26.36) |  | 2100.67 (1271.6 to 3037.87) | 14.97 (9.06 to 21.65) |
| Taiwan (Province of China) | 4512.29 (4178.06 to 4883.91) | 22.13 (20.49 to 23.95) |  | 5142.29 (4368.24 to 6066.29) | 21.76 (18.48 to 25.66) |
| Tajikistan | 640.51 (412.73 to 943.73) | 11.93 (7.69 to 17.58) |  | 841.7 (523.63 to 1261.37) | 8.28 (5.15 to 12.42) |
| Thailand | 8407.63 (5657.16 to 11194.79) | 14.81 (9.97 to 19.72) |  | 9972.53 (7036.15 to 14564.32) | 14.96 (10.55 to 21.84) |
| Timor-Leste | 151.92 (94.51 to 227.81) | 19.45 (12.1 to 29.16) |  | 166.22 (112.54 to 255.23) | 11.89 (8.05 to 18.26) |
| Togo | 624.51 (365.35 to 1004.56) | 17.13 (10.02 to 27.55) |  | 1078.36 (668.01 to 1712.15) | 12.88 (7.98 to 20.45) |
| Tokelau | 0.09 (0.05 to 0.13) | 5.43 (3.09 to 8.43) |  | 0.08 (0.05 to 0.14) | 5.8 (3.6 to 10.46) |
| Tonga | 2.6 (1.51 to 4.02) | 2.63 (1.53 to 4.07) |  | 2.62 (1.55 to 4.24) | 2.47 (1.46 to 3.99) |
| Trinidad and Tobago | 390.35 (352.57 to 432.15) | 32.4 (29.26 to 35.87) |  | 521.04 (396.79 to 653.26) | 37.4 (28.48 to 46.89) |
| Tunisia | 1788.19 (1234.99 to 2820.67) | 21.42 (14.79 to 33.78) |  | 2045.98 (1342.95 to 3264.6) | 17.28 (11.34 to 27.57) |
| Turkey | 20331.85 (14877.16 to 28771.33) | 35.38 (25.89 to 50.06) |  | 16784.04 (11847.08 to 23233.43) | 20.07 (14.17 to 27.79) |
| Turkmenistan | 428.87 (287.11 to 647.52) | 11.59 (7.76 to 17.5) |  | 788.98 (534.21 to 1189.21) | 15.3 (10.36 to 23.05) |
| Tuvalu | 0.64 (0.35 to 0.94) | 6.74 (3.68 to 9.87) |  | 0.55 (0.33 to 0.79) | 4.42 (2.63 to 6.36) |
| Uganda | 12996.48 (9037.58 to 18373.43) | 75.16 (52.27 to 106.26) |  | 21468.67 (14079.59 to 35530.44) | 49.56 (32.51 to 82.03) |
| Ukraine | 15129.97 (12754.43 to 18126.28) | 28.7 (24.2 to 34.39) |  | 13532.61 (9691.01 to 18095.14) | 31.41 (22.5 to 42.01) |
| United Arab Emirates | 385.25 (271.79 to 552.19) | 20.59 (14.53 to 29.52) |  | 1063.34 (737.59 to 1478.62) | 11.04 (7.66 to 15.35) |
| United Kingdom | 21267.57 (20716.96 to 21765.4) | 37.12 (36.16 to 37.99) |  | 29910.28 (28521.87 to 31109.28) | 44.08 (42.04 to 45.85) |
| United Republic of Tanzania | 15125.03 (9437.72 to 24563.94) | 58.54 (36.53 to 95.07) |  | 22081.94 (13199.17 to 36993.98) | 37.78 (22.58 to 63.29) |
| United States of America | 117807.96 (114102.8 to 120666.16) | 46.36 (44.91 to 47.49) |  | 157622.62 (150052.53 to 163595.92) | 47.38 (45.11 to 49.18) |
| United States Virgin Islands | 32.76 (23.57 to 48.88) | 30.89 (22.23 to 46.09) |  | 18.71 (12.86 to 28.71) | 21.78 (14.97 to 33.42) |
| Uruguay | 1146.27 (978.72 to 1360.9) | 36.51 (31.18 to 43.35) |  | 1373.07 (1160.4 to 1640.07) | 40.32 (34.07 to 48.16) |
| Uzbekistan | 685.48 (482.43 to 954.54) | 3.27 (2.3 to 4.55) |  | 3807.04 (2708.73 to 5032.28) | 11.12 (7.91 to 14.7) |
| Vanuatu | 4.87 (2.58 to 7.61) | 3.2 (1.69 to 5) |  | 10.71 (6.3 to 16.3) | 3.42 (2.01 to 5.21) |
| Venezuela (Bolivarian Republic of) | 3353.66 (2748.21 to 3798.58) | 17.83 (14.61 to 20.2) |  | 6830.89 (5054.32 to 8962.07) | 25.65 (18.98 to 33.66) |
| Viet Nam | 12192.2 (8355.83 to 18209.34) | 17.87 (12.25 to 26.69) |  | 18456.96 (12048.25 to 30153.8) | 18.41 (12.02 to 30.07) |
| Yemen | 4319.26 (2634.95 to 6252.4) | 31.68 (19.33 to 45.86) |  | 5420.63 (3296.48 to 7769.99) | 16.11 (9.8 to 23.1) |
| Zambia | 5269.77 (3406.9 to 8555.2) | 66.4 (42.93 to 107.8) |  | 7841.09 (4386.41 to 14833.52) | 40.18 (22.48 to 76.01) |
| Zimbabwe | 2097.51 (1466.94 to 3114.57) | 20.28 (14.18 to 30.11) |  | 6189.26 (3932.17 to 9966.98) | 39.69 (25.21 to 63.91) |

ASDR, age-standard DALYs rate. DALYs, disability-adjusted life years. UI, uncertainty interval.
